# Two novel qualitative transcriptional signatures robustly applicable to non‐research‐oriented colorectal cancer samples with low‐quality RNA

**DOI:** 10.1111/jcmm.16467

**Published:** 2021-03-14

**Authors:** Jun Cheng, Yating Guo, Guoxian Guan, Haiyan Huang, Fengle Jiang, Jun He, Junling Wu, Zheng Guo, Xing Liu, Lu Ao

**Affiliations:** ^1^ Affiliated Foshan Maternity and Child Healthcare Hospital Southern Medical University (Foshan Maternity & Child Healthcare Hospital) Foshan China; ^2^ Department of Bioinformatics Fujian Key Laboratory of Medical Bioinformatics School of Basic Medical Sciences Fujian Medical University Fuzhou China; ^3^ Key Laboratory of Ministry of Education for Gastrointestinal Cancer Fujian Medical University Fuzhou China; ^4^ Department of Colorectal Surgery The Affiliated Union Hospital of Fujian Medical University Fuzhou China

**Keywords:** colorectal cancer, low‐quality RNA, non‐research‐oriented clinical samples, relative expression orderings, transcriptional signature

## Abstract

Currently, due to the low quality of RNA caused by degradation or low abundance, the accuracy of gene expression measurements by transcriptome sequencing (RNA‐seq) is very challenging for non‐research‐oriented clinical samples, majority of which are preserved in hospitals or tissue banks worldwide with complete pathological information and follow‐up data. Molecular signatures consisting of several genes are rarely applied to such samples. To utilize these resources effectively, 45 stage II non‐research‐oriented samples which were formalin‐fixed paraffin‐embedded (FFPE) colorectal carcinoma samples (CRC) using RNA‐seq have been analysed. Our results showed that although gene expression measurements were significantly affected, most cancer features, based on the relative expression orderings (REOs) of gene pairs, were well preserved. We then developed two REO‐based signatures, which consisted of 136 gene pairs for early diagnosis of CRC, and 4500 gene pairs for predicting post‐surgery relapse risk of stage II and III CRC. The performance of our signatures, which included hundreds or thousands of gene pairs, was more robust for non‐research‐oriented clinical samples, compared to that of two published concise REO‐based signatures. In conclusion, REO‐based signatures with relatively more gene pairs could be robustly applied to non‐research‐oriented CRC samples.

## 1 INTRODUCTION

With technological advancement and reduced cost, transcriptome sequencing (RNA‐seq) has become the primary technology for gene expression measurements.^1^, ^2^ This platform generally requires input of pre‐selected high‐quality RNA samples, designated as research‐oriented clinical samples, to obtain reliable research results. For example, adequate amounts of RNA extracted from fresh‐frozen (FF) samples containing at least 60% or 70% of the tumour nuclei,^3^ and high RNA integrity (RIN) scores, such as RIN > 6 or RIN > 7,^4^ are required. However, realistically, millions of samples obtained in hospitals and tissue banks worldwide are considered to be non‐research‐oriented clinical samples with low‐quality RNA,^5^ characterized by RNA degradation or fragmentation,^6^ low amounts of RNA,^7^ low tumour purities,^8^ or above multiple features simultaneously. Although amplification technology can be used for these samples containing low amounts of RNA, it may introduce amplification bias.^9^ Thus, for these non‐research‐oriented clinical samples, the accuracy of gene expression measurements would be significantly challenged. As a result, the transcriptional signatures based on risk scores that are summarized from the expression levels of signature genes are rarely applied to these non‐research‐oriented samples. Rather, these samples are primarily limited to pathological or immunohistochemical analysis^10^; however, these samples contain valuable pathological information and follow‐up data,^11^ which are precious resources in disease‐related research. Therefore, it is imperative that transcriptional signatures should be developed that are applicable to non‐research‐oriented clinical samples with low‐quality RNA.

Colorectal carcinoma (CRC) is one of the most common malignant tumours with high morbidity and mortality,^12^ which is mainly transformed from acquired pre‐cancerous lesions. Inflammatory bowel disease (IBD), including ulcerative colitis (UC) and Crohn's disease (CD), is a main type of pre‐cancerous colorectal lesions, which could result in dysplasia, eventually develops and progresses to CRC.^13^ Some studies have been reported that long‐term exposure to chronic inflammation is the primary risk factor for CRC pathogenesis.^14^ Meanwhile, some patients with stage II and III CRC after surgery treatment commonly have relapse risk. Currently, it is essential to timely discriminate early CRC patients from patients with inflammation and accurately predict the recurrence risk for stages II and III CRC patients after surgery.^15^, ^16^ However, established non‐invasive tests, such as the guaiac‐based faecal occult blood test and faecal haemoglobin, usually lack proper sensitivity and specificity for early diagnosis. Carcinoembryonic antigens, CA125 and CA19.9, which have already been applied into clinical practice, are not highly promising diagnostic or prognostic targets for personalized medicine.^17^ Therefore, there is a critical need to develop highly robust and reliable biomarkers for diagnosis and prognosis of CRC patients.

Specific methods, such as TSP (top scoring pairs),^18^ *k*‐TSP^19^ and other adjusted methods,^20^ that take advantage of the qualitative transcriptional features of genes, which are based on the relative expression orderings (REOs) of gene pairs within sample, have been proposed to develop disease‐related signatures. Our previous work has demonstrated that most of the REO patterns of gene pairs were insensitive to samples with degraded RNA, low amounts of RNA or varying tumour purities.^7^, ^10^, ^21^ Hence, to facilitate clinical translational application, some concise REO‐based signatures with several or dozens of gene pairs have been developed in our previous studies, including seven gene pairs for early diagnosis of CRC,^16^ 44 gene pairs for predicting post‐surgery relapse risk of stage II and III CRC^22^ and so on.^23^ Nevertheless, gene expression measurements could be widely and significantly affected by the low‐quality non‐research‐oriented clinical samples. If the expression measurements of one or several signature genes are severely influenced, and even become zero, the performance of these concise REO‐based signatures with several gene pairs may be seriously weakened or even rendered unfeasible. In consideration of the rapid development and decreasing cost of high‐throughput sequencing technology, we proposed that the REO‐based signatures should include relatively more gene pairs, potentially even hundreds or thousands of gene pairs, to obtain robust performance for the non‐research‐oriented clinical samples with low‐quality RNA.

To this end, herein we analysed 45 stage II CRC non‐research‐oriented samples that were formalin‐fixed paraffin‐embedded (FFPE) samples without location pre‐selection or pre‐purification of tumour cells, measured using RNA‐seq, and evaluated the influences of low‐quality samples on their gene expression measurements. For these widely preserved non‐research‐oriented clinical samples, two REO‐based signatures with relatively more gene pairs were developed and robustly applied to diagnosis and recurrence prediction of individuation. It had great value for clinical translational applications.

## 2 MATERIALS AND METHODS

### 2.1 Samples and data measurement

A total of 45 stage II CRC FFPE samples, denoted as CRC45, including 24 non‐relapse and 21 relapse samples, were collected from FFPE tissue blocks which have been preserved at room temperature for about 6 years. The RIN scores, overall alignment rate of sequencing reads and clinical information of the 45 samples are shown in Table S1. Each FFPE tissue block without location pre‐selection or pre‐purification of tumour cells was cut into 6‐10 slides of approximately 5 μm thickness. Then, the slides with frozen preservation were directly sent to the sequencing company. The whole process was about 72 hours. Next, according to the manufacture's protocol, total RNA was isolated from each sample using the RNAprep pure FFPE kit (Tiangen Biotech) and the quality of the RNA was assessed by electrophoresis on an Agilent 2100 Bioanalyzer system (Agilent Technologies). Ribosomal RNA was removed using the Globin‐Zero Gold rRNA Removal kit & directional library, and the stranded RNA‐seq library was constructed using the NEB Next^®^ Ultra™ RNA Library Prep kit. Paired‐end sequencing (2 × 150) was performed on the Illumina HiSeq X Ten system (Illumina). Subsequently, the generated raw RNA‐seq (FASTQ) files were pre‐processed using the Trimmomatic,^24^ and the reads were aligned to the reference genome (GRCh38) using HISAT2.^25^ Finally, number of the Fragments Per Kilobase of transcript per Million fragments mapped (FPKM) values of the genes were calculated. This research has been approved by the Institutional Review Board at Fujian Medical University Union Hospital, and written consent forms were obtained from all participants.

### 2.2 Public data and pre‐processing

All public gene expression profiles measured by RNA‐seq were downloaded from the Gene Expression Omnibus (GEO, http://www.ncbi.nlm.nih.gov/geo/) and The Cancer Genome Atlas (TCGA, http://cancergenome.nih.gov/) database, as described in detail in Table 1. In total, 662 CRC samples, 149 normal samples and 353 IBD samples, including those from patients with UC and CD, were assessed. The FPKM or number of Reads Per Kilobase of transcript per Million reads mapped (RPKM) values were directly downloaded. Next, the Ensembl gene IDs were mapped to the Entrez gene IDs. The Ensembl gene ID which was mapped to zero or multiple Entrez gene IDs were deleted. If multiple Ensembl gene IDs were mapped to a Entrez gene ID, the expression value of the gene was defined as the arithmetic mean of the values of the multiple Ensembl gene IDs. The FPKM values of 13 CRC samples (CRC13) from our previous study were used directly.^16^

**TABLE 1:** Description of datasets used in this study

Data	Sample size		
	Normal	IBD	Cancer
TCGA			
FF			
‐	51	–	–
I	–	–	106
II	–	–	231
III	–	–	176
IV	–	–	90
NA	–	–	22
FFPE			
I‐IV	–	–	19
GSE72819	–	73	–
GSE109142	20	206	–
GSE83687	60	74	–
GSE50760	18	–	18
CRC13	–	–	13
CRC45	–	–	45

Note

NA, CRC samples without the information of stage.

### 2.3 Evaluation of REO‐based cancer features in non‐research‐oriented clinical samples

For a gene pair with two genes, for example gene *i* and gene *j*, the REO was denoted as G*_i_* > G*_j_* (or G*_i_* < G*_j_*), where the expression measurement of gene *i* was higher (or lower) than that of gene *j* within a sample. A gene pair exhibiting the same REO pattern in most samples from one group, for example 95% or 99%, was defined as a stable gene pair. A stable gene pair with an opposite REO pattern between two groups was defined as a stable opposite gene pair. Using a hypergeometric cumulative distribution model, we evaluated whether a specific REO pattern, for example G*_i_* > G*_j_* in one group of samples, was significantly opposited into the pattern G*_i_* < G*_j_* in the other group of samples. A gene pair of which the REO pattern was significantly opposited between two groups was defined as a significant opposite gene pair. A stable or significant opposite gene pair, G*_i_* > G*_j_*, representing the REO pattern in CRC, was defined as an REO‐based cancer feature. The stable and significant opposite gene pairs between stage II FF CRC samples and FF normal samples from TCGA were selected to evaluate the maintenance of the REO‐based cancer features in each non‐research‐oriented clinical sample, respectively. The retention rate was calculated as follows:

(1) ratio=k/m

where *m* was the number of stable or significant opposite gene pairs, and *k* was the number of gene pairs that maintained the cancer features in a non‐research‐oriented sample. Notably, the gene pair was removed if two expression measurements of the gene pair both carried a value of zero. Additionally, the gene pairs containing a gene that was not measured were also removed.

### 2.4 Identification of an REO‐based signature for the early diagnosis of CRC

The stable opposite gene pairs between stage I FF CRC samples and FF normal samples were selected as candidate gene pairs for the REO‐based signature for the early diagnosis of CRC. The gene expression profiles were then transformed into gene expression rank profiles according to their expression levels. All genes were sorted in ascending order, and the rank difference (*RD*) for a gene pair (*i*, *j*) in sample *t* was calculated as:

(2) $$RD_{\mathrm{tij}}=R_{\mathrm{ti}}‐R_{\mathrm{tj}}$$

where *R_ti_* and *R_tj_* were the expression ranks of gene *i* and gene *j* in sample *t*, respectively. Next, the *RD* for each gene pair was calculated in each stage I sample or normal sample, respectively.

(3) $${\text{avgRD}}_{\mathrm{ij}}=\sqrt{\left|\text{mean}\;[{\text{RD}}_{\mathrm{ij}}\left(\text{cancer}\right)]\right|\times \left|\text{mean}\;[{\text{RD}}_{\mathrm{ij}}\left(\text{normal}\right)]\right|}$$

where |mean[RD*_ij_*(cancer)]| and |mean[RD*_ij_*(normal)]| represented the absolute mean RD value of the opposite gene pair (*i*, *j*) in all the samples of stage I CRC and normal groups, respectively. Subsequently, the geometric mean of the absolute mean[RD*_ij_*(cancer)] and the absolute mean [RD*_ij_*(normal)] was calculated to evaluate the opposite degree of the gene pair between two types of samples. The larger the opposite degree of the REO for the gene pair between stage I samples and normal samples, the larger the geometric mean (avgRD). If a gene appeared in multiple gene pairs, a redundancy removal process was performed that only the gene pair with the largest avgRD was retained. Based on the assumption that, for non‐research‐oriented clinical samples, the REO‐based signature should include relatively more gene pairs, such as hundreds of gene pairs, all candidate gene pairs were pooled together as the REO‐based signature for the early diagnosis of CRC. For the REO pattern of CRC, a sample was assigned to the CRC group when the retention rate of gene pairs in the signature was more than a certain cut‐off, for example 60%; otherwise, it was assigned to the non‐cancer group. Similarly, the gene pairs including two genes both with expression values of zero, or the gene pairs containing a non‐measured gene, were excluded.

### 2.5 Development of an REO‐based signature for predicting post‐surgery relapse risk of stages II and III CRC

Compared with a stringent cut‐off of 99% for stable opposite gene pairs, a false discovery rate (FDR) control of 1% for significant opposite gene pairs was relatively loosen. Because the transcriptional differences between stage I and stage IV samples were less than that between normal and CRC, it is difficult to select hundreds or thousands of stable opposite gene pairs to construct the REO‐based signature for predicting post‐surgery relapse risk of CRCs. Thus, in order to obtain sufficient gene pairs with more advantageous discriminating ability for CRC relapse, we selected the significant opposite gene pairs from the differentially expressed genes (DEGs) between stage I and stage IV samples identified by the RankCompV2 algorithm.^26^ The stage I CRC samples without new tumour events and metastasis were analysed. Briefly, the RankCompV2 algorithm firstly identified the significantly stable gene pairs in two distinct groups by using the binomial test with FDR control (<20%), respectively. Next, based on the overlap between the two lists of stable gene pairs, the concordant and opposite REOs were identified between two distinct groups. Finally, DEGs that may disrupt the REOs of genes were identified using the Fisher's exact test with FDR control (<5%). Using a hypergeometric cumulative distribution model, the significant opposite gene pairs with at least one DEG were identified between stage I and stage IV samples and further filtered by 13 paired FFPE and FF samples from TCGA. For a significant opposite gene pair, the same REO pattern should be kept in at least ten paired FF and FFPE samples.

The candidate gene pairs were then sorted in descending order according to their relative coverage difference between two groups, which was calculated as follows:

(4) $$C_{\text{IVI}}=C_{\text{IV}}‐C_{I}$$

where *C*_IV_ and *C*_I_ represent the coverage of a gene pair with the REO pattern (G*_i_* > G*_j_*) in stage IV and stage I samples, respectively. For example, for a gene pair with the REO pattern (G*_i_* > G*_j_*) in *m* of *n* stage IV samples, its coverage will be *m*/*n*. The samples that had expression values of zero for both genes of a gene pair were not counted. *C*_IVI_ represents the relative frequency difference of the gene pair (*i*, *j*) between stage IV and stage I samples.

All candidate gene pairs were classified into several groups representing the candidate signatures. The classification performance of each candidate signature was evaluated by the voting rule that states that a sample was to be classified as high‐risk relapse when the proportion of the same REO pattern (G*_i_* > G*_j_*) was more than the threshold. Considering that the accuracy for stage IV should be as high as possible, the classification threshold was evaluated separately as 50% ± 1%, and the candidate signature with better classification performance and relatively higher robustness was selected as the signature. Finally, a Cox proportional‐hazards regression model was used to evaluate the association between the predictive signature and the disease‐free interval time (DFI) of patients with stage II and III CRC ^27^ and the Schoenfeld residuals test was used to test the proportional hazard assumption in the Cox model. The Kaplan‐Meier method and log‐rank test were used to estimate the survival curves. All statistical analyses were performed by R 3.6.0. The R scripts were provided in Supplementary scripts.

### 2.6 Protein‐protein interactions and functional enrichment analysis

A regulatory protein‐protein interaction (PPI) network for the genes of interest was constructed based on the integrated data from the HSNet (Human Signaling Network, version 6)^28^ and the SIGNOR databases.^29^ Particularly, the Ensembl gene IDs corresponding to the unique Entrez gene IDs of protein‐coding genes were analysed. Functional enrichment analysis for the genes of interest was subsequently performed based on KEGG (the Kyoto Encyclopedia of Genes and Genomes).^30^ The hypergeometric distribution model was used to calculate the enrichment significance of biological pathways, whereas the Benjamini‐Hochberg method was adopted to estimate the FDR.

## 3 RESULTS

### 3.1 Quality evaluation of non‐research‐oriented clinical samples

Here, we firstly evaluated the RNA qualities of the 45 non‐research‐oriented FFPE samples of stage II CRC measured in our laboratory. Compared to the requirement of high‐quality FF samples with RIN scores of 6.0 for RNA‐seq, the RIN scores of total RNA in these non‐research‐oriented clinical samples ranged from 2.1 to 2.7. These results suggest that the RNA of these non‐research‐oriented samples was seriously degraded and fragmented. Meanwhile, in more than half of the 45 non‐research‐oriented FFPE samples, more than 28% of genes had an expression value of zero, and the highest percentage of genes with an expression value of zero in FFPE samples reached 53.90%. In contrast, approximately 10% of the 231 stage II FF samples from TCGA had slightly more than 28% of genes with expression values of zero. These results indicated that the gene expression measurements of these non‐research‐oriented samples were seriously affected.

**FIGURE 1:**
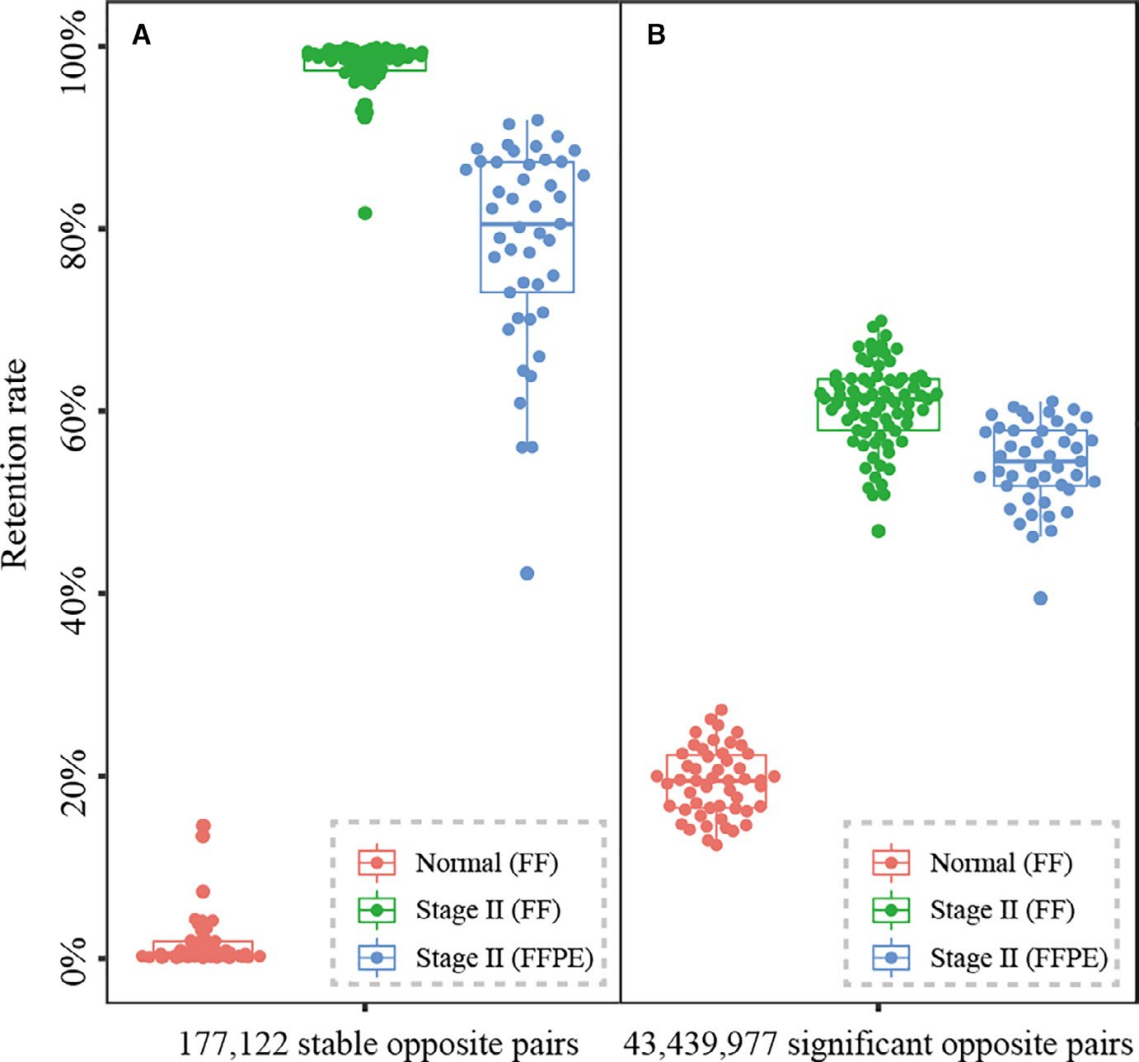
Evaluation of the REO‐based cancer features in non‐research‐oriented clinical samples using the 177 122 stable opposite gene pairs (A) or the 43 439 977 significant opposite gene pairs (B) which were selected between 77 FF tumour and 51 FF normal samples. The retention rates of gene pairs with the specific cancer pattern (G*_i_* > G*_j_*) in the public FF CRCs (green), in‐house FFPE CRCs (blue) and normal FF samples (red) were shown

Next, the REO‐based cancer features in these non‐research‐oriented samples were evaluated through comparison with the stable opposite REO patterns and significant opposite REO patterns between the FF stage II CRC samples and normal samples from TCGA, respectively. To weaken the biased influences of sample size for two groups, we selected significant or stable opposite gene pairs between the first third (77) of the 231 stage II FF samples and 51 normal samples. With a 95% cut‐off for stable opposite gene pairs, 177 122 stable opposite gene pairs were identified between 77 stage II FF CRCs (G*_i_* > G*_j_*) and 51 FF normal samples (G*_i_* < G*_j_*). Taking these REO patterns of stable opposite gene pairs in CRC samples as cancer features, approximately 95% of the stable opposite gene pairs were also retained in the remaining 154 stage II FF CRCs (as shown in Figure S1), and the retention rate was approximately 80% in the 45 non‐research‐oriented clinical samples (as shown in Figure 1A). Similarly, with the control of FDR < 0.05, 43 439 977 significant opposite gene pairs were identified between 77 stage II FF CRCs and 51 FF normal samples. Taking these REO patterns of the significant opposite gene pairs in CRC samples as cancer features, we found that compared to approximately 60% of the retention rate for the 77 and the remaining 154 FF CRC samples, approximately 55% of the significant opposite gene pairs were retained in the non‐research‐oriented clinical samples (as shown in Figures 1B and S1). On the contrary, the retention rate of stable and significant opposite gene pairs (G*_i_* > G*_j_*) in the normal samples were only kept about 5% and 20%, respectively. These results indicated that most of the REO‐based cancer features were well preserved in the non‐research‐oriented clinical samples.

### 3.2 Development of an REO‐based signature for the early diagnosis of CRC

Recently, we have reported a concise REO‐based signature consisting of seven gene pairs for discriminating early CRC from IBD samples, including UC and CD samples.^16^ However, the seven gene pairs were able to only distinguish 31.11% of the 45 non‐research‐oriented CRCs as cancer because the expression measurements of two signature genes were zero in all non‐research‐oriented samples. The results showed that the seven gene pairs were not robust enough against the non‐research‐oriented samples with low‐quality RNA. It is, therefore, necessary to develop a more robust signature for the early diagnosis of CRC.

**FIGURE 2:**
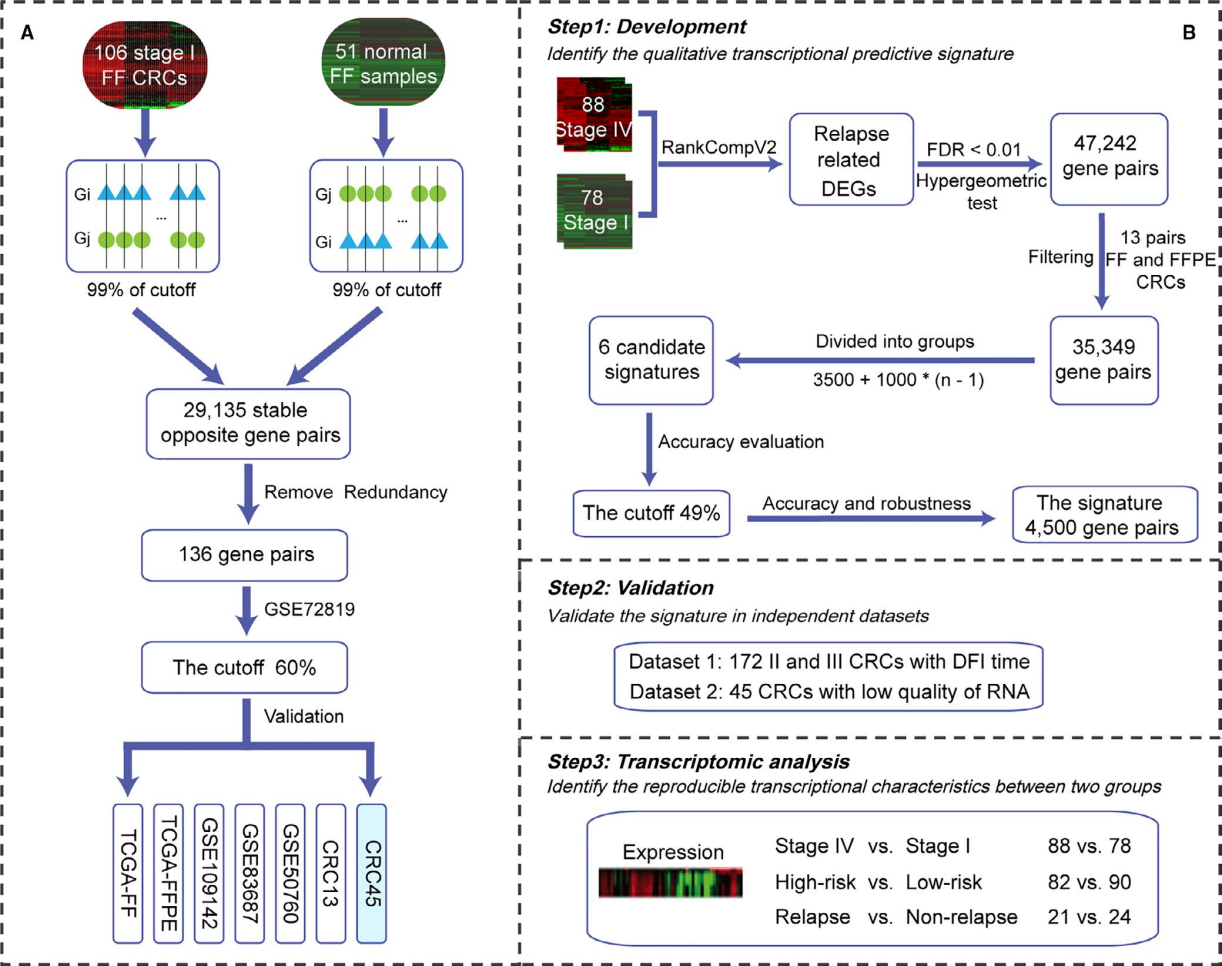
The flow chart for the development of the REO‐based signatures for the early diagnosis of CRC (A) or predicting post‐surgery relapse risk of stage II and III CRC (B), respectively

The development of the early diagnosis signature for CRC was summarized in Figure 2A. First, with a cut‐off of 99%, 29 135 stable opposite gene pairs were identified between the 106 stage I FF CRCs and 51 FF normal samples from TCGA. Second, we narrowed down the number of gene pairs via a redundancy removal process and 136 stable opposite gene pairs were identified as the early diagnosis signature for CRC (see Section 2, Table S2). The cut‐off of the voting rule was set to 60% as the 73 colitis samples in the GSE72819 dataset were all correctly assigned to the non‐cancer group. For a sample, if the retention ratio of the specific cancer pattern (G*_i_* > G*_j_*) was ≥60%, the sample was labelled as CRC; otherwise, it was labelled as non‐cancer (see Section 2). In the training datasets, 106 CRC and 51 normal samples were all correctly assigned to the CRC group and the non‐cancer group, respectively.

The 136 gene pair signature was further validated in multiple public datasets. For the remaining CRC (untrained FF samples) surgical samples from TCGA, 99.42% of the 519 FF CRCs and 89.47% of the 19 FFPE CRCs were correctly classified as cancer. The 13 CRC samples with various tumour purities from the CRC13 dataset were all correctly assigned to the CRC group. For the 206 colitis and 20 normal biopsy samples from the GSE109142 dataset, all were correctly assigned to the non‐cancer group. For the GSE83687 dataset, 97.30% of the 74 IBD surgical samples and 100% of the 60 normal surgical samples were correctly classified into the non‐cancer group. For the GSE50760 dataset, two thirds of the 18 primary cancer surgical samples were classified as cancer samples and 100% of the normal surgical samples were correctly designated as non‐cancer samples. These results demonstrate that our signature can effectively facilitate the early diagnosis of CRC, regardless of whether the samples are obtained via surgery or biopsy, or whether the samples have varying tumour purities.

Next, the 136 gene pair signature was further verified in the 45 non‐research‐oriented samples. Similar results were observed that 95.56% of the 45 non‐research‐oriented samples were correctly classified as cancer. As shown in Table 2, 99.60% of the total 502 non‐cancer samples, and 97.92% of the total 720 cancer samples, were correctly identified. The retention rates of the early diagnosis signature for all analysed samples were shown in Figure S2. Taken together, these results reveal that the REO‐based signature with 136 gene pairs can be robustly applied to non‐research‐oriented clinical samples with low‐quality RNA.

**TABLE 2:** The performance of the early diagnosis signature in multiple datasets

Dataset	Accuracy/sample size		
	Normal	IBD	Cancer
*The performance of the signature in the training datasets*			
TCGA‐1	100% (51/51)	–	100% (106/106)
GSE72819	–	100% (73/73)	–
*The performance of the signature in the validation datasets*			
TCGA‐FF	–	–	99.42% (516/519)
TCGA‐FFPE	–	–	89.47% (17/19)
GSE109142	100% (20/20)	100% (206/206)	–
GSE83687	100% (60/60)	97.30% (72/74)	–
GSE50760	100% (18/18)	–	66.67% (12/18)
CRC13	–	–	100% (13/13)
CRC45	–	–	95.56% (43/45)
Total	99.60% (500/502)		98.19% (707/720)

Note

IBD represents inflammatory bowel diseases samples. TCGA‐1: stage I CRC and normal FF samples; TCGA‐FF: the non‐stage I FF CRC samples; TCGA‐FFPE: the FFPE CRC samples.

### 3.3 Identification of an REO‐based signature for predicting post‐surgery relapse risk of stage II and III CRC

The process for developing an REO‐based signature for predicting the post‐surgery relapse risk of stage II and III CRC was summarized in Figure 2B. First, 619 CRC samples with survival information were selected from TCGA.^27^ Based on the hypothesis that the relapse of stage II and III CRC could be attributed to micro‐metastasis,^31^, ^32^ 644 DEGs were identified between the 88 stage IV samples (the metastatic samples) and 78 stage I samples (the non‐metastatic samples) using the RankcompV2 algorithm. With the control of FDR < 0.01, 47 242 significant opposite gene pairs, including at least one DEG, were identified between stage IV and stage I samples, of which 35 349 gene pairs were kept after further filtering using 13 paired FF and FFPE CRC samples (as shown in Table S3). Next, the sorted significant opposite gene pairs were categorized into six groups for six candidate signatures based on the rule: the *n*th candidate signature consisted of 3500 + 1000 * (*n* − 1) gene pairs. Specifically, the six candidate signatures included 3500, 4500, 5500, 6500, 7500 and 7849 gene pairs, respectively. For example, the first candidate signature was assigned the top 1 to 3500 gene pairs, and the top 3501 to 8000 gene pairs were assigned to the second candidate signature and so on. The accuracy of classification for each candidate signature was evaluated in the training dataset. The cut‐off was set to 49% in comparison with 50% and 51%, as the higher accuracy for stage IV samples. For a given sample, if more than 49% of the gene pairs in the signature contained the specific REO pattern for relapse, the sample was labelled as high‐risk relapse, and vice versa (see Section 2).

Using the cut‐off of 49%, the accuracies of two candidate signatures were found to both be above 80% in stage I and stage IV. The first candidate signature including 3500 gene pairs could correctly classify 82.05% of the stage I samples and 81.82% of the stage IV samples in the training dataset, whereas the second candidate signature including 4500 gene pairs correctly classified 80.77% of the stage I and 81.82% of the stage IV samples. For the sake of robustness, the second candidate signature that included relatively more gene pairs was ultimately chosen as the signature (see Table S4).

**FIGURE 3:**
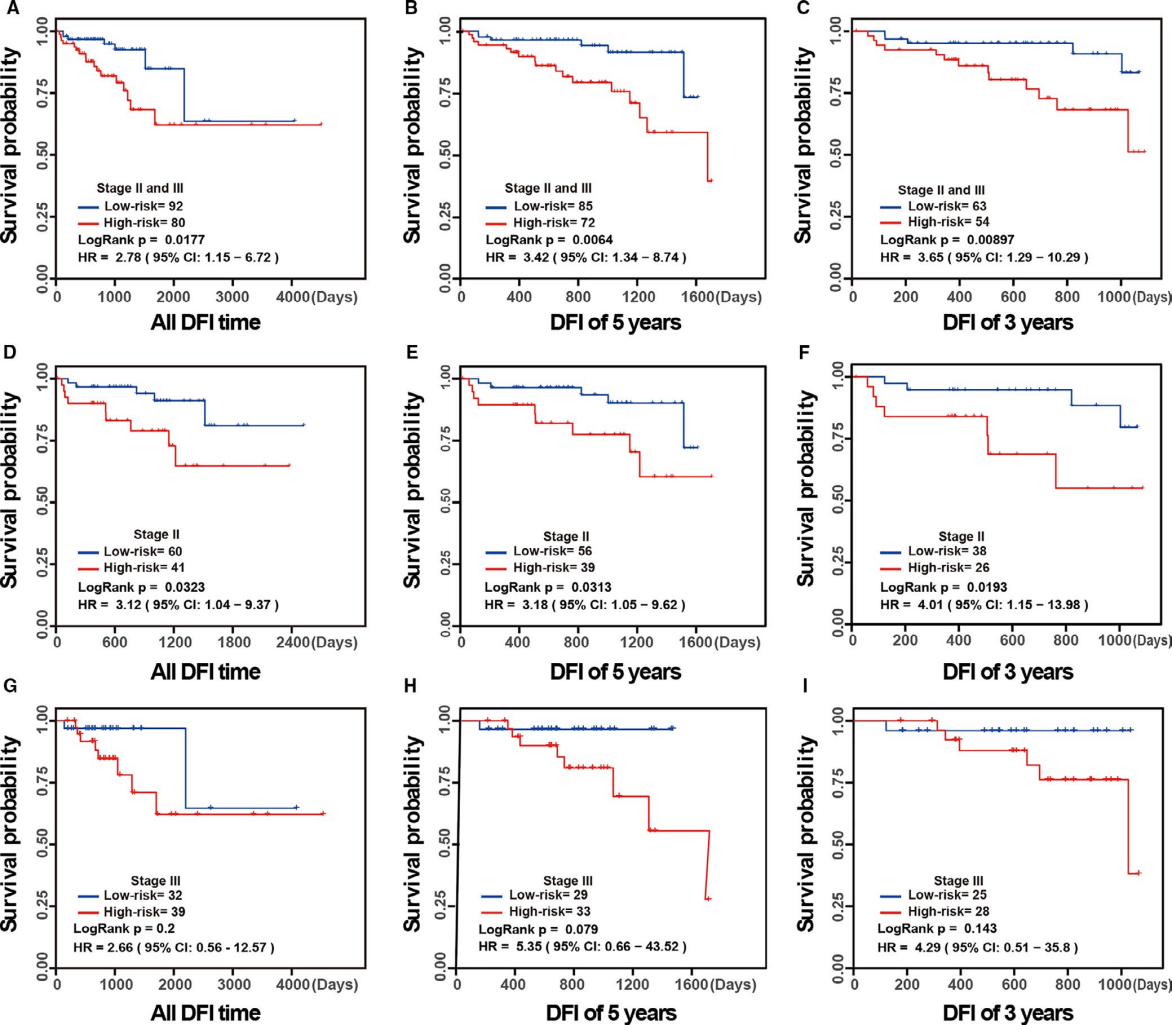
The predictive performance of the 4500 gene pair signature. A‐C, Kaplan‐Meier curves of DFI for patients with stage II and III CRC. D‐F, Kaplan‐Meier curves of DFI for patients with stage II CRC. G‐I, Kaplan‐Meier curves of DFI for patients with stage III CRC

For 172 CRCs with DFI time obtained from TCGA, 80 samples and 92 samples were classified by the signature as high relapse risk and low relapse risk, respectively, of which the former had significantly worse DFI survival than the latter (univariate Cox, HR = 2.78, 95% CI = 1.15‐6.72, log‐rank test *P* = 0.018, Figure 3A). And there was no separate residual for the high relapse risk and the low relapse risk groups in the 172 CRCs from TCGA (the Schoenfeld residuals test*, P* = 0.25). The retention rates for 172 CRC samples were show n in Figure S3. Meanwhile, similar results were also observed within the 5‐year (univariate Cox, HR = 3.42, 95% CI = 1.34‐8.74, log‐rank test *P* = 0.0064, Figure 3B) and 3‐year DFI time (univariate Cox, HR = 3.65, 95% CI = 1.29‐10.29, log‐rank test *P* = 0.0090, Figure 3C). Moreover, our signature was able to robustly predict the post‐surgery relapse risk in stage II CRC (Figure 3D‐F). Unfortunately, for stage III patients, the significant difference of DFI time between high and low relapse risk groups was not observed, as shown in Figure 3G‐I. Actually, one patient with 85 years who had cancer relapse in about four months was classified into the low relapse risk group. After excluding the sample, the DFI time between the low and high relapse risk groups in stage III CRC patients was moderately different (univariate Cox, HR = 5.2, 95% CI = 0.65‐41.71, log‐rank test *P* = 0.083, Figure S4A). Moreover, the 5‐year and 3‐year DFI time was significantly different (Figure S4B,C). Meanwhile, we also performed multivariable cox proportional‐hazards regression analyses for the CRC relapse signature with 4500 gene pairs. Because the MSI information of 37 patients were lost, 135 II‐III colorectal cancer patients, including 27 patients with MSI‐high, 21 patients with MSI‐low and 87 patients with MSI‐stable, were evaluated. After correction for stage, gender, age and MSI, there was a modest difference on DFI time (HR = 2.65, 95% CI = 0.91‐7.69, log‐rank *P* = 0.074, as shown in Table S5). To reduce the impact of sample reduction due to the missing MSI information, multivariable cox proportional‐hazards regression analyses were additionally performed in 172 II‐III colorectal cancer patients. Significant difference on DFI time was observed after correction for stage, gender and age (HR = 2.65, 95% CI = 1.06‐6.62, log‐rank test *P* = 0.037).

**FIGURE 4:**
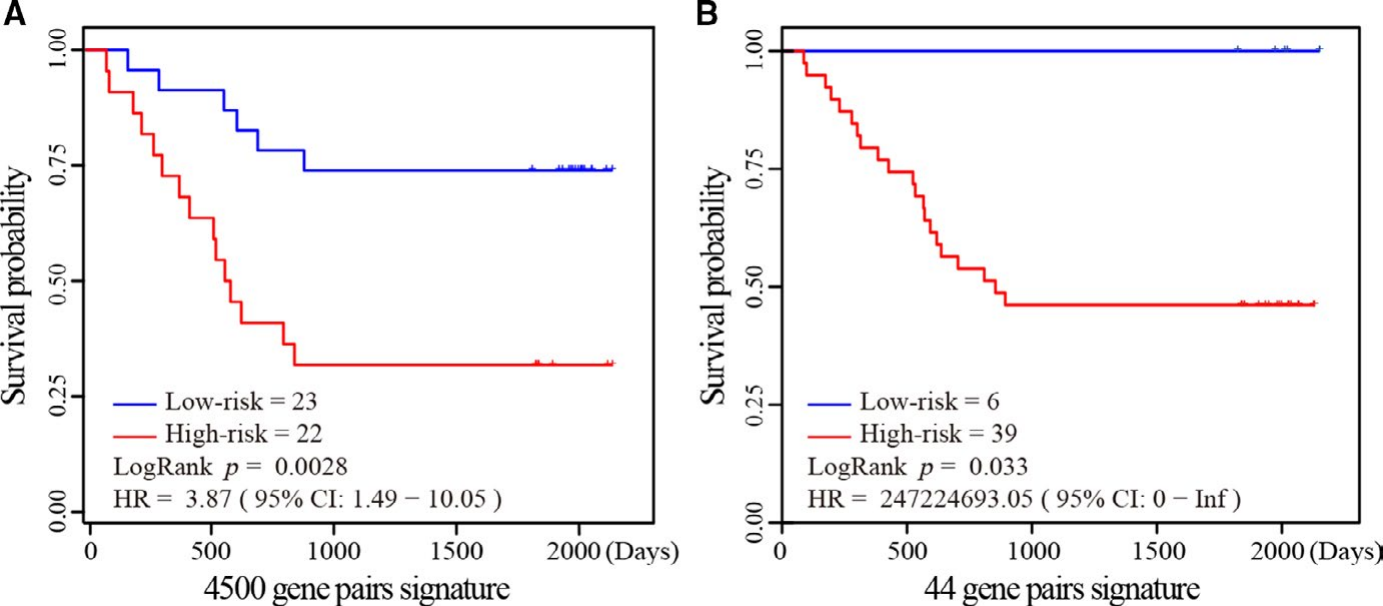
The predictive performances of the 4500 gene pairs signature (A) and 44 gene pairs signature (B) in the non‐research‐oriented clinical samples

For the 45 stage II non‐research‐oriented CRC samples measured in our laboratory, the 23 patients predicted as low relapse risk had significantly better DFI survival than the 22 patients predicted as high relapse risk (univariate Cox, HR = 3.87, 95% CI = 1.49‐10.05, log‐rank test *P* = 0.0028, as shown in Figure 4A). There was no separate residual for the high relapse risk and the low relapse risk groups in the 45 CRCs (the Schoenfeld residuals test*, P* = 0.67). Moreover, there were 70.83% of the 24 non‐relapse samples, and 71.42% of the 21 relapse samples, correctly identified. Compared to the predictive performance of our previous 44 gene pairs, which predicted six patients (25% of non‐relapse samples) as low relapse risk and 39 as high relapse risk (univariate Cox, log‐rank test *P* = 0.033, Figure 4B), the results further demonstrated that our signature with 4500 gene pairs had higher prognosis capacity, and more robust predictive performance in non‐research‐oriented samples.

### 3.4 The potential relapse mechanism of CRC

**FIGURE 5:**
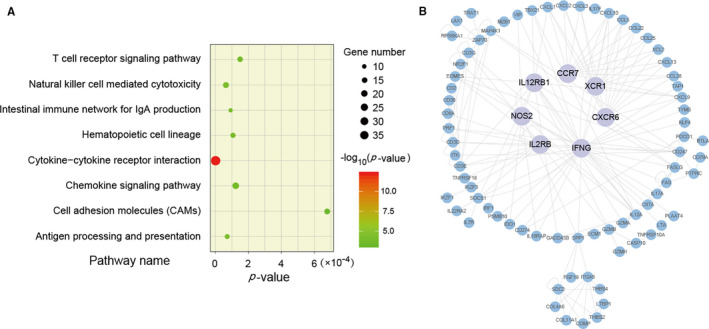
The function analysis of the common DEGs. A, KEGG function enrichment analysis with the 499 DEGs. B, The largest sub‐network in PPI analysis

Using the RankCompV2 algorithm with FDR < 0.05, 3109 DEGs were identified between the 80 high and 92 low relapse risk stage II and III CRCs, whereas 784 DEGs were identified between 88 stage IV and 78 stage I samples. A total of 503 DEGs were overlapped between the two DEG lists, of which 499 DEGs had consistent dysfunction direction. The consistency was 99.20%, which was significantly higher than what was expected by chance (binomial test, *P* < 2.2 × 10^−16^). The functional enrichment analysis of 499 DEGs showed that they were significantly enriched in the immune‐related pathways, including ‘natural killer cell‐mediated cytotoxicity’ and ‘T cell receptor signalling pathway’. (FDR < 0.05, as shown in Figure 5A). Notably, based on the 1811 immune‐related genes downloaded from the ImmPort database, 117 of the 499 DEGs were immune‐related genes. By mapping the 499 DEGs into the integrated data from both the HSNet and SIGNOR databases, a regulatory PPI network including 102 DEGs with 196 edges was constructed, 61.78% of which were immune‐related genes. The largest sub‐network was shown in Figure 5B. We also observed that seven hub DEGs (*IFNG*, *IL2RB*, *IL12RB1*, *CCR7*, *XCR1*, *CXCR6* and *NOS2*) with more than ten PPI interactions were all immune‐related genes.

Furthermore, using the RankCompV2 algorithm (FDR < 0.05), 956 DEGs were detected between 24 non‐relapse and 21 relapse samples measured in our laboratory, of which 42 DEGs overlapped with the above‐mentioned 499 DEGs. The concordance score between the two DEG lists was as high as 85.71% (36 DEGs), which was unlikely to occur by chance (binomial test, *P* < 2.2 × 10^−07^). Meanwhile, the PPI analysis showed that the 18 DEGs directly interacted with 301 other genes (as shown in Figure S5) and these 319 genes were also significantly enriched in immune‐related functional pathways, including ‘T cell receptor signalling pathway’ and ‘B cell receptor signalling pathway’ (Table S6). These results further suggest that the potential relapse mechanism of CRC might be closely related to immune dysfunction.

## 4 DISCUSSION

In this study, we first demonstrated that most of the REO‐based cancer features were preserved in non‐research‐oriented clinical samples, although their gene expression measurements were seriously affected. Second, we developed an REO‐based signature with 136 gene pairs for early diagnosis of CRC and an REO‐based signature with 4500 gene pairs for predicting post‐surgery relapse risk of stage II and III CRC. The results demonstrate that the REO‐based signatures with relatively more gene pairs, rather than several or dozens of gene pairs, could be robustly applied to non‐research‐oriented clinical samples containing low‐quality RNA.

Additionally, we found that CRC relapse could be closely related to immune dysfunction. Previous studies have shown that the immune‐related DEGs, *NOS2*, *CCR7* and *IFNG*, which were hub nodes in Figure 5B, could affect the prognosis of CRC patients. For example, Thomas et al^33^ have shown that *NOS2* is highly expressed in different cancers and may be a powerful prognostic biomarker, and *NOS2* polymorphisms could be used to predict whether metastatic CRC patients may benefit from first‐line chemotherapy.^34^ The expression of *CCR7* in tumour infiltrating CD8+ T cells may lead to a tumour‐specific immune response with potential antitumour activity, leading to a favourable prognosis for metastatic CRC patients.^35^ Moreover, *CCR7* has been suggested as a potential target in cancer therapy as it plays an important role in the metastasis of several cancers.^35^, ^36^ Ganapathi et al^37^ have indicated that low expression of *IFNG* could be the reason for the progression of stage IV CRC.^37^ *IL12RB1* has been reported that its mutation played a causal role in non‐polyposis CRC pre‐disposition.^38^ Furthermore, the other three hub genes, *IL2RB, XCR1* and *CXCR6*, have been reported to be related with the prognosis of other cancers, such as early breast cancer,^39^ salivary adenoid cystic carcinoma,^40^ bladder and hepatocellular cancers.^41^, ^42^

Obviously, the subtle quantitative information associated with gene expression would be missed in REO patterns. However, this genetic information is quite generally error‐prone and sensitive to batch effects^43^, ^44^ and data normalization.^23^ Nevertheless, the REO patterns that take advantage of the qualitative features of genes within samples could be readily applied for individualized clinical applications.^45^ With the rapid development and sharp decrease in costs associated with sequencing technology, signatures with hundreds or thousands of genes could now be feasible in clinical settings. Moreover, including additional genes in the REO‐based signatures could increase robustness against the dysfunction of some signature genes, while weakening the influence of non‐research‐oriented clinical samples with low‐quality RNA, particularly in widely preserved FFPE samples. However, it is certainly necessary to also develop new technologies to extract high‐quality RNA from non‐research‐oriented samples and optimize the protocols or workflow, including the extraction, amplification and labelling methods.^6^, ^46^, ^47^

In contrast with RNA biomarkers in FFPE samples, the DNA biomarkers have a minor risk of degradation. Two DNA‐related biomarkers, Cologuard^48^ and Epi proColon^®^ 2.0 CE,^49^ have been approved by the FDA for colorectal cancer screening. However, they remain too expensive and technically complex.^50^ Some DNA methylation biomarkers that could be directly detected by blood and stool have also been reported.^51^, ^52^, ^53^, ^54^ Additionally, other DNA biomarkers, such as mutations of *KRAS*, *TP53* and *APC*, and hypermethylation of tumour suppressor genes at the promoter regions, have been developed.^55^, ^56^ But until now, there are no recognized prognostic biomarkers in clinical practice for CRC patients.^15^ Therefore, development of robust RNA or DNA biomarkers for clinical application is worthy to further study.

Colorectal adenoma is a major type of pre‐cancerous colorectal lesions. The risk of developing colorectal cancer for patients with adenomas is two to four times higher than those patients without adenomas. However, a lack of adenomas measured by RNA‐seq in the public database caused that no adenoma samples datasets were included to develop the early diagnosis signature. We also noticed that the sample size of non‐research‐oriented clinical samples was small in this study. In our CRC45 dataset, only approximately 70% of the non‐relapse and relapse samples were correctly identified using the 4500 gene pairs signature. Thus, additional non‐research‐oriented samples are needed to further validate and optimize our REO‐based signatures. Another limitation was that only the sequencing data were analysed. The predictive power of our signatures is not high in all datasets across platforms. For instance, in the GSE50760 dataset, one third of the 18 cancer patients were incorrectly identified by the 136 gene pairs signature, which may result from platform differences. In next work, we will pool microarray and sequencing data together to develop REO‐based signatures for the non‐research‐oriented clinical samples, to allow them to readily be applied across different platforms.

In summary, the REO‐based signature with relatively more gene pairs could be robustly applied to the non‐research‐oriented clinical samples containing low‐quality RNA, and it holds significant value for clinical translational applications.

## CONFLICT OF INTEREST

The authors confirm that there are no conflicts of interest.

## AUTHOR CONTRIBUTION

**Jun Cheng:** Conceptualization (equal); Writing‐original draft (lead); Writing‐review & editing (equal). **Yating Guo:** Data curation (equal); Formal analysis (equal); Visualization (equal); Writing‐review & editing (equal). **Guoxian Guan:** Data curation (equal). **Haiyan Huang:** Visualization (equal). **Fengle Jiang:** Formal analysis (equal). **Jun He:** Writing‐original draft (supporting). **Junling Wu:** Visualization (equal). **Zheng Guo:** Conceptualization (equal). **Xing Liu:** Data curation (equal). **Lu Ao:** Conceptualization (equal); Writing‐review & editing (equal).

## Supporting information

Figure S1

Figure S2

Figure S3

Figure S4

Figure S5

Table S1

Table S2

Table S3

Table S4

Table S5

Table S6

Supplementary Material

Supplementary Material

## Data Availability

The in‐house data used and analysed during the current study are available from the corresponding authors upon reasonable request.
