# Supplementary figures and images for: Two novel qualitative transcriptional signatures robustly applicable to non‐research‐oriented colorectal cancer samples with low‐quality RNA
Source: J Cell Mol Med. 2021 Mar 14;25(7):3622–33. doi: 10.1111/jcmm.16467 (PMC8034468; doi:10.1111/jcmm.16467)

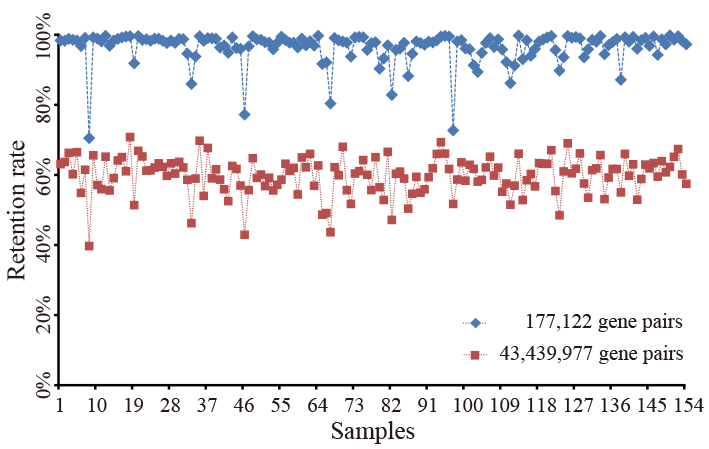

Supplement: Supplementary file 1 — Figure S1 [file JCMM-25-3622-s005.tif]

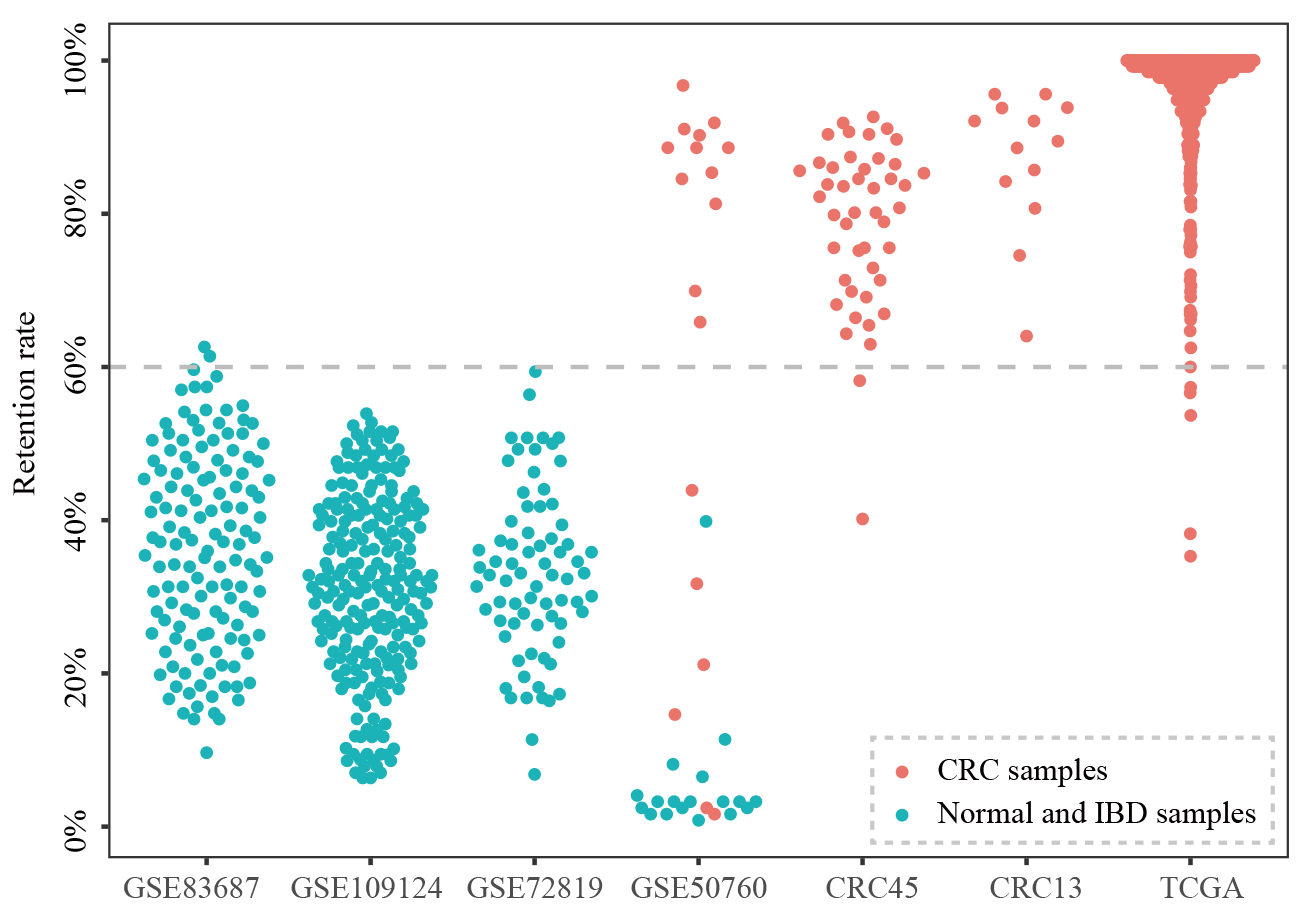

Supplement: Supplementary file 2 — Figure S2 [file JCMM-25-3622-s002.tif]

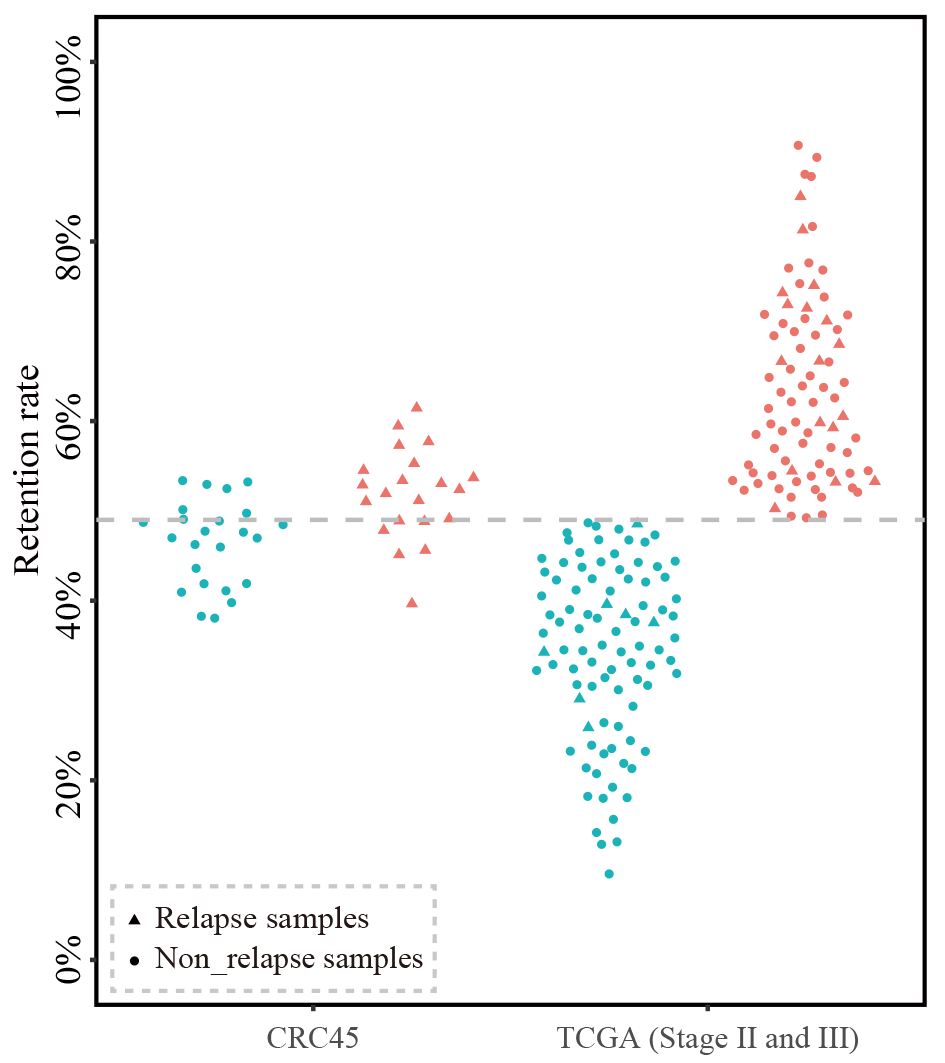

Supplement: Supplementary file 3 — Figure S3 [file JCMM-25-3622-s009.tif]

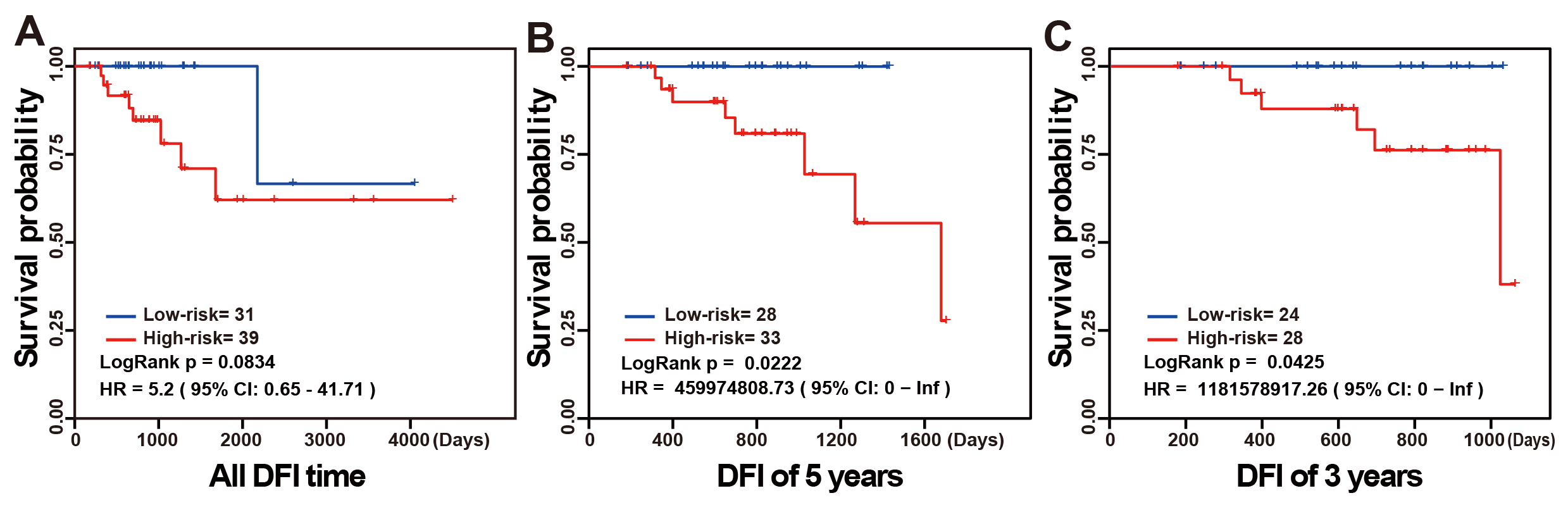

Supplement: Supplementary file 4 — Figure S4 [file JCMM-25-3622-s006.tif]

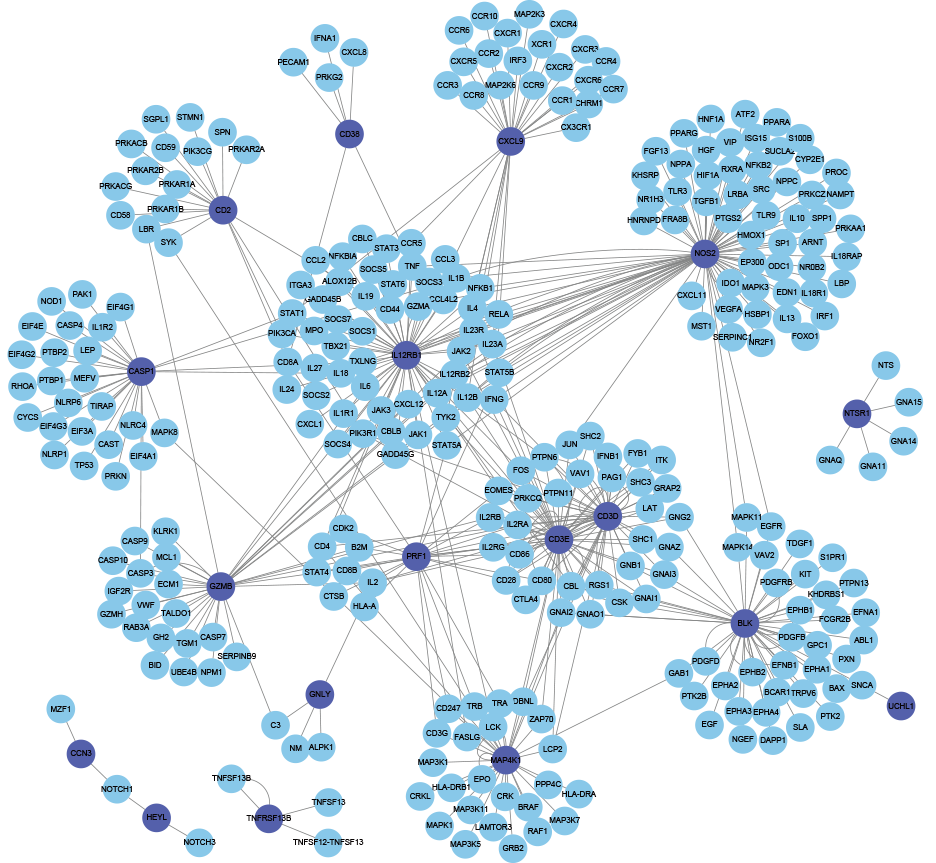

Supplement: Supplementary file 5 — Figure S5 [file JCMM-25-3622-s007.tif]
