# Supplementary material for: Two novel qualitative transcriptional signatures robustly applicable to non‐research‐oriented colorectal cancer samples with low‐quality RNA
Source: J Cell Mol Med. 2021 Mar 14;25(7):3622–33. doi: 10.1111/jcmm.16467 (PMC8034468; doi:10.1111/jcmm.16467)
Supplement: Supplementary file 6 — Table S1 [file JCMM-25-3622-s011.doc]

| Table S1. Quality of the non-research-oriented samples measured in our lab | | | | | |
| --- | --- | --- | --- | --- | --- |
| Sample | Relapse | DFI_time | Overall alignment rate | Start_time | RIN |
| CRC1 | 0 | 2151 | 0.3117 | 2008 | Na |
| CRC2 | 0 | 2150 | 0.3242 | 2008 | Na |
| CRC3 | 0 | 2131 | 0.1632 | 2008 | Na |
| CRC4 | 0 | 1999 | 0.2354 | 2008 | Na |
| CRC5 | 0 | 2021 | 0.3299 | 2008 | 2.3 |
| CRC6 | 0 | 1907 | 0.1928 | 2008 | 2.3 |
| CRC7 | 0 | 1973 | 0.3299 | 2009 | 2.3 |
| CRC8 | 0 | 2126 | 0.7318 | 2010 | 2.4 |
| CRC9 | 0 | 2065 | 0.8358 | 2010 | 2.4 |
| CRC10 | 0 | 2068 | 0.5113 | 2010 | 2.4 |
| CRC11 | 0 | 1823 | 0.6267 | 2010 | 2.3 |
| CRC12 | 0 | 2037 | 0.6768 | 2010 | 2.4 |
| CRC13 | 1 | 566 | 0.4367 | 2010 | 2.4 |
| CRC14 | 0 | 2024 | 0.6494 | 2010 | 2.3 |
| CRC15 | 0 | 2011 | 0.7188 | 2010 | 2.4 |
| CRC16 | 0 | 1999 | 0.815 | 2010 | 2.3 |
| CRC17 | 0 | 1989 | 0.7838 | 2010 | 2.4 |
| CRC18 | 0 | 1981 | 0.4934 | 2010 | 2.4 |
| CRC19 | 0 | 1947 | 0.5161 | 2010 | 2.4 |
| CRC20 | 0 | 1933 | 0.4863 | 2010 | 2.4 |
| CRC21 | 0 | 1850 | 0.6952 | 2010 | 2.2 |
| CRC22 | 0 | 1849 | 0.7716 | 2010 | 2.3 |
| CRC23 | 0 | 1841 | 0.6657 | 2010 | 2.4 |
| CRC24 | 0 | 1837 | 0.6427 | 2010 | 2.4 |
| CRC25 | 1 | 301 | 0.6361 | 2011 | 2.4 |
| CRC26 | 1 | 384 | 0.7561 | 2011 | 2.4 |
| CRC27 | 0 | 2029 | 0.749 | 2011 | 2.4 |
| CRC28 | 1 | 533 | 0.9212 | 2011 | 2.1 |
| CRC29 | 1 | 593 | 0.7173 | 2011 | 2.4 |
| CRC30 | 1 | 87 | 0.7458 | 2011 | 2.4 |
| CRC31 | 1 | 619 | 0.4921 | 2011 | 2.4 |
| CRC32 | 1 | 570 | 0.736 | 2012 | 2.4 |
| CRC33 | 1 | 230 | 0.6027 | 2012 | 2.4 |
| CRC34 | 1 | 196 | 0.9038 | 2012 | 2.4 |
| CRC35 | 1 | 426 | 0.5355 | 2012 | 2.3 |
| CRC36 | 1 | 704 | 0.3835 | 2012 | 2.4 |
| CRC37 | 1 | 637 | 0.2007 | 2012 | 2.4 |
| CRC38 | 1 | 174 | 0.6426 | 2012 | 2.3 |
| CRC39 | 1 | 524 | 0.8819 | 2012 | 2.4 |
| CRC40 | 1 | 809 | 0.732 | 2012 | 2.4 |
| CRC41 | 1 | 893 | 0.7735 | 2013 | 2.4 |
| CRC42 | 1 | 854 | 0.7146 | 2013 | 2.3 |
| CRC43 | 1 | 98 | 0.431 | 2013 | 2.3 |
| CRC44 | 1 | 279 | 0.9221 | 2014 | 2.3 |
| CRC45 | 1 | 314 | 0.6195 | 2014 | 2.4 |

DFI_time refers to disease-free interval time in days from postoperative to recurrence.

Start_time represents the initial follow-up time. Na: represents the loss information.
