# Supplementary material for: Two novel qualitative transcriptional signatures robustly applicable to non‐research‐oriented colorectal cancer samples with low‐quality RNA
Source: J Cell Mol Med. 2021 Mar 14;25(7):3622–33. doi: 10.1111/jcmm.16467 (PMC8034468; doi:10.1111/jcmm.16467)
Supplement: Supplementary file 7 — Table S2 [file JCMM-25-3622-s013.doc]

| Table S2. The early diagnosis signature of CRC with 136 gene pairs | | | |
| --- | --- | --- | --- |
| G*_i_* | G*_j_* | G*_i_* | G*_j_* |
| ACAN | SUSD5 | EHMT2 | TMEM140 |
| AHCY | ZG16 | TOMM34 | GINM1 |
| AMPD2 | EXOC7 | UBE2C | PLAC8 |
| CCND1 | FBLIM1 | ESM1 | SLC30A10 |
| BGN | MFAP4 | ADAMTS6 | LRRC4C |
| BYSL | SYNPO | CCT5 | UGDH |
| CAD | ARHGAP35 | TPX2 | TMEM171 |
| CBFB | KRAS | DDN | LINC02000 |
| CDH3 | GCG | BOP1 | SLC9A1 |
| CKS2 | ATP5PF | ATP11A | STARD13 |
| COL7A1 | RASGRP2 | OSBPL3 | DNAJB4 |
| COL10A1 | SLITRK3 | CPNE7 | CD40LG |
| COL11A1 | FMN2 | SLCO4A1 | ACHE |
| CSE1L | WASL | NOB1 | GABARAP |
| CST1 | OTOP2 | CYP2S1 | CHP2 |
| DARS1 | CHMP3 | NOX4 | SPOCK3 |
| DKC1 | SMIM14 | TNFRSF12A | CLDN23 |
| DNAH5 | CHAT | FGFRL1 | PLCD1 |
| ECT2 | TMOD3 | RNF43 | LINC01133 |
| SLC29A1 | CAV1 | TMEM161A | IGFBP6 |
| ETV4 | PYY | KIF26B | NCAM2 |
| KAT2A | KLC4 | FERMT1 | AHCYL2 |
| GRB7 | PDK2 | TRMT1 | MOB2 |
| GRIN2B | DAO | RCC2 | RETSAT |
| GRIN2D | NPY6R | EXOSC5 | TCTA |
| CXCL1 | CLCA4 | SALL4 | SLCO4C1 |
| CXCL3 | ADTRP | CEMIP | WSCD1 |
| INHBA | FGF7 | CBX8 | PDE4DIP |
| IRAK1 | TNFRSF1A | PPM1H | PDE3B |
| CAPRIN1 | CAST | KIAA1257 | TEX11 |
| MCM7 | GOT1 | TRIB3 | PKIB |
| MDFI | EML1 | GRHL3 | HTR3A |
| MMP7 | CDKN2B-AS1 | SINHCAF | TMBIM4 |
| MMP11 | MRGPRF | TGIF2 | GYPC |
| MYBL2 | PTPRH | STRA6 | NPY2R |
| MYC | SQOR | CNTD2 | NEFH |
| OTX1 | KRT24 | TTYH3 | AGPAT1 |
| PAFAH1B3 | TMEM238L | PRR7 | STBD1 |
| PPA1 | HIGD1A | PDRG1 | BEX4 |
| PVT1 | ANKRD44 | ADAMTS12 | HMCN1 |
| PYCR1 | SLC50A1 | CDCA7 | SIAE |
| RAN | ATP5MC3 | PLEKHN1 | GPR174 |
| RBL1 | FAM126B | WDR75 | NRIP1 |
| RPS2 | ITM2C | AJUBA | CAP2 |
| SIM2 | IGSF10 | SAPCD2 | MINDY1 |
| SOX9 | SLC25A23 | ZNF598 | TOM1L2 |
| SPTBN2 | ITGA8 | ZC3HAV1L | PIK3C3 |
| TCF3 | VAMP2 | MAFG-DT | CACNB1 |
| TEAD4 | MIR22HG | FOXQ1 | TMIGD1 |
| TGFBI | PPIC | RHPN1 | ZFYVE28 |
| CCT3 | ATP5F1A | BTBD16 | CNTNAP3 |
| WNT2 | CMA1 | LARGE2 | DOCK10 |
| WNT7B | LRRC3B | CCDC78 | DMRTA1 |
| LRP8 | LYST | ANKRD13B | LOC103611081 |
| SLC7A5 | CHGA | MFSD12 | STAB1 |
| ENC1 | GPT | IQGAP3 | TLCD2 |
| EIF3B | VPS4B | RNF183 | PNOC |
| SLC5A6 | DHDDS | KRT80 | CADM3 |
| CLDN2 | SYNM | NOTUM | MYOC |
| RRP9 | SMPD1 | KLC3 | MAGEE2 |
| GDF15 | PADI2 | KLHL35 | KCNE2 |
| GTF2IRD1 | TRAPPC12 | WDR62 | TTLL6 |
| NFE2L3 | SLC51B | FAM83H | CPTP |
| TRAP1 | DLAT | VWA2 | RNF152 |
| NOP56 | MRPL35 | NUP43 | FCHO2 |
| RNASEH2A | GON7 | SFTA2 | PHOX2B |
| CCT7 | CALM1 | FAM83H-AS1 | FAM13B |
| CCT4 | NCOA4 | LOC101927480 | ASXL3 |

Note: G*_i_* > G*_j_* represents the CRC feature.
