# Supplementary material for: Two novel qualitative transcriptional signatures robustly applicable to non‐research‐oriented colorectal cancer samples with low‐quality RNA
Source: J Cell Mol Med. 2021 Mar 14;25(7):3622–33. doi: 10.1111/jcmm.16467 (PMC8034468; doi:10.1111/jcmm.16467)
Supplement: Supplementary file 8 — Table S3 [file JCMM-25-3622-s004.doc]

| Table S3. The stage information of 13 paired samples | | |
| --- | --- | --- |
| FF CRC Samples | FFPE CRC samples | Stage |
| TCGA-A6-2674-01A | TCGA-A6-2674-01B | Stage IV |
| TCGA-A6-2684-01A | TCGA-A6-2684-01C | Stage I |
| TCGA-A6-3809-01A | TCGA-A6-3809-01B | Stage IIB |
| TCGA-A6-3810-01A | TCGA-A6-3810-01B | Stage IIA |
| TCGA-A6-5656-01A | TCGA-A6-5656-01B | Stage I |
| TCGA-A6-5659-01A | TCGA-A6-5659-01B | Stage I |
| TCGA-A6-6650-01A | TCGA-A6-6650-01B | Stage IIA |
| TCGA-A6-6780-01A | TCGA-A6-6780-01B | Stage IIA |
| TCGA-A6-6781-01A | TCGA-A6-6781-01B | Stage IIIC |
| TCGA-A6-2672-01A | TCGA-A6-2672-01B | Stage IIIB |
| TCGA-A6-2677-01A | TCGA-A6-2677-01B | Stage IIIC |
| TCGA-A6-5661-01A | TCGA-A6-5661-01B | Stage IIA |
| TCGA-A6-5665-01A | TCGA-A6-5665-01B | Stage IIA |
