# Supplementary material for: Two novel qualitative transcriptional signatures robustly applicable to non‐research‐oriented colorectal cancer samples with low‐quality RNA
Source: J Cell Mol Med. 2021 Mar 14;25(7):3622–33. doi: 10.1111/jcmm.16467 (PMC8034468; doi:10.1111/jcmm.16467)
Supplement: Supplementary file 9 — Table S4 [file JCMM-25-3622-s012.doc]

| Table S4. The signature for predicting post-surgery relapse risk of stage II and III CRC with 4,500 gene pairs. | | | | | |
| --- | --- | --- | --- | --- | --- |
| G*_i_* | G*_j_* | G*_i_* | G*_j_* | G*_i_* | G*_j_* |
| HSPA1A | RNASEH1 | RNF217-AS1 | C2orf66 | COL10A1 | CXCL10 |
| KIF3C | CCL22 | NOG | IGLL1 | GRP | GS1-124K5.4 |
| CCN3 | UBE2D3-AS1 | TCEAL2 | IL9R | NPR3 | WDR78 |
| PTH1R | ANK1 | MRO | CCL25 | LAMP5 | SLC22A4 |
| SNAPC3 | NUDT18 | SLC6A17 | KLRC1 | LAMP5 | CD300LF |
| VEGFC | LAG3 | PCDHB5 | GPR25 | EDARADD | TNIP3 |
| VLDLR | SLAMF1 | PCP4L1 | CD244 | C5orf46 | GPR65 |
| ZSCAN9 | IL2RB | NOG | NR2F2-AS1 | C5orf46 | ARHGAP15 |
| AKT3 | CIITA | NTSR1 | MIR34AHG | BNIP3 | TSPY26P |
| AKT3 | GBP5 | ISM2 | MEFV | CAV2 | CXCR6 |
| PQBP1 | GSR | ACTC1 | LOC100506585 | CPM | CXCR6 |
| SMUG1 | CD3D | ZFHX3 | IL2RB | MAP1B | ICOS |
| CSDC2 | GPR25 | CALB2 | ITGB7 | EMC2 | COQ2 |
| HECA | C2CD4A | CAPS | CD3D | ARMCX2 | CXCR6 |
| AGPAT4 | ICOS | CDH2 | CD180 | RBMX2 | CD3D |
| NDUFA4L2 | TENT5C | COL11A2 | TRG-AS1 | SNORD14E | GNLY |
| NXN | CCL22 | CST2 | CALHM6 | SNORD14E | CD209 |
| ZBTB10 | IL2RB | GRIK2 | RAB44 | SNORD14E | APOBEC3F |
| POGLUT2 | DOK2 | GRP | IFIT1 | DACT3 | LINC00996 |
| ZNF665 | CD1B | GRP | MME | BCHE | HDC |
| TRIM46 | DBH-AS1 | GRP | TLR2 | IGFL1 | C22orf24 |
| TMEM121 | TNFRSF9 | GRP | SCARF1 | HMGA2-AS1 | CHRM3-AS2 |
| MEX3B | UBASH3A | GRP | TNFSF11 | CD72 | SIRPG |
| CCDC50 | NUDT18 | GRP | PRELID3A | HAND2 | DBH-AS1 |
| ZNF320 | IL2RB | GRP | PLCE1 | ARC | ZNF683 |
| PHYHD1 | SLAMF1 | GRP | BCL6B | ZNF114 | IL17A |
| CHSY3 | RASGRP1 | MMP17 | IL12RB1 | C20orf202 | LINC00996 |
| BEX5 | C16orf54 | CCN3 | TNFRSF18 | NALCN | LOC10012809 |
| SERP2 | DNASE1L3 | NPR3 | PCDHGA12 | LINC02657 | IFNG |
| SMIM10 | CTLA4 | NPR3 | IPCEF1 | TCEAL2 | CLECL1 |
| CD2BP2-DT | IL2RB | NTSR1 | WAKMAR2 | ACTC1 | PIK3R2 |
| GRIK2 | CLEC6A | PLXNB3 | INSC | BNIP3 | E2F5 |
| LINC00840 | CHRM3-AS2 | SEPTIN4 | CIITA | CACNB3 | HTT |
| NUDT11 | GBP1P1 | PRELP | ARHGAP44 | CALB2 | EBI3 |
| TUBB2B | GPR25 | PSD | MMP25 | SEPTIN7 | MMP12 |
| IGFL1 | ANKRD35 | SPP1 | GSR | COMP | RAB11FIP4 |
| SAMD12-AS1 | NCR3 | SPP1 | NIPSNAP1 | FUT1 | CCDC134 |
| ADK | PBK | SPP1 | PROCR | GRP | FAAP24 |
| COL9A3 | LAG3 | THBS4 | LILRB1 | GRP | BCLAF3 |
| GRP | LOC339192 | TPM2 | CYB5R3 | GRP | GEN1 |
| HSPA1A | CDCA3 | TPM2 | FDFT1 | NPR3 | PDE6G |
| UBE2E2 | CTLA4 | TRPC1 | ICOS | TAGLN | TCP1 |
| CHST1 | TIGIT | VIP | SLAMF7 | THBS4 | CXCR6 |
| ADGRL2 | CIITA | SSPN | IDO1 | SLC28A2 | DNASE1L3 |
| CHST15 | ITGAL | DEGS1 | MMP12 | NOG | GRIN3A |
| AZIN1 | LAP3 | CHRD | CTLA4 | HAND2 | CFP |
| VASH2 | TRG-AS1 | NOL3 | PARP3 | FAM107A | SIRPG |
| UXS1 | CD2 | ITGBL1 | SORCS2 | NLRP1 | CXCR6 |
| LAYN | GZMM | DLEC1 | GPR25 | KCNE4 | IDO1 |
| GRASP | CXCR6 | FAM107A | CD3G | LAMP5 | SARM1 |
| ZBTB34 | CXCR6 | MMRN1 | ICOS | TAFA5 | IL2RB |
| ALMS1-IT1 | KIR2DL4 | SRGAP2 | CCL22 | ZDHHC5 | GSR |
| CADM3 | CHRM3-AS2 | LAMP5 | TMPPE | C20orf194 | SIRPG |
| CARTPT | LINC02273 | DKK2 | GBP5 | ANGPTL4 | IL1RN |
| GRP | IGFL2 | NOX4 | P2RY10 | COPZ2 | CXCL9 |
| GALNT17 | CD1B | ANGPTL4 | NKG7 | RAB6B | CXCR6 |
| NBPF10 | CHRM3-AS2 | POPDC2 | CXCR6 | BEX4 | MFSD9 |
| ALMS1-IT1 | TRG-AS1 | CCDC8 | CCR7 | C14orf132 | GBP5 |
| LINC01655 | TIFAB | SNORD14E | POLE2 | CCDC90B | COQ2 |
| FABP4 | IFNG | FAM110B | TNFRSF9 | MRPL9 | GSR |
| HOTAIR | LINC00861 | ZNF251 | GZMB | MYCT1 | FOXP3 |
| AGTR1 | KIR2DL4 | AFAP1L1 | FOXP3 | LY6G6C | IL2RB |
| ACTC1 | UBASH3A | CDC26 | COQ2 | ZNF333 | CXCR6 |
| CALB2 | ARHGAP9 | IGFL1 | ZFHX2 | ST6GAL2 | LAMC3 |
| CALB2 | PINK1-AS | IGFL1 | LINC01555 | ST6GAL2 | RIPOR3 |
| FCGR2B | ICOS | SHISA2 | SIT1 | GNG8 | UBASH3A |
| GRP | NR3C2 | PLIN4 | MEI1 | PCP2 | DNASE1L3 |
| GRP | TMEM231 | WASHC1 | CCDC134 | DACT3 | CCR7 |
| NPR3 | LTA | ERICH6-AS1 | UBASH3A | TTC16 | CHRM3-AS2 |
| PPFIA4 | CEACAM3 | LINC02257 | LINC01215 | NSUN6 | C2CD4A |
| LRRC17 | CXCR6 | NOG | ATRIP | AMIGO2 | NT5DC1 |
| MRVI1 | CXCL9 | CACNB3 | CIAO3 | IGFL1 | TNFRSF9 |
| KIFAP3 | BCKDHB | CALB2 | ZNF304 | IGFL1 | ARHGAP22 |
| SUSD5 | NRG1 | CST2 | CCL22 | C5orf46 | TLR8 |
| TMEM79 | CD3D | DDIT3 | XPNPEP1 | C16orf74 | CXCR6 |
| ZNF542P | SIRPG | GRP | ZXDA | PCP4L1 | LRRC4 |
| ZNF707 | FAM83F | GRP | DOC2B | ZNF605 | IDO1 |
| IGFL1 | CFP | GRP | TRPA1 | PCAT6 | C2CD4A |
| IGFL1 | POU2AF1 | GRP | ANO7 | ISM2 | DLG3-AS1 |
| LOC105375624 | CXCR6 | GRP | TM4SF18 | BCHE | TNFRSF13B |
| GRP | RN7SL3 | GRP | C9orf116 | NGF | IFNG |
| SPAG8 | GPR25 | GRP | NBPF9 | NKX3-2 | FEM1A |
| PCP4L1 | TBX21 | GRP | CD99P1 | BMI1 | LEPROTL1 |
| HAND2-AS1 | LOC100128059 | INHBB | FAM219B | CALB2 | SNAI3 |
| MGC16275 | LHX2 | INHBB | EPG5 | CAV2 | CALHM6 |
| NOG | SULT1C2P1 | INHBB | WDR62 | DDIT3 | SPNS2 |
| CALB2 | FEZ1 | ITGA7 | IL2RB | DYNC1I1 | TNIP3 |
| CALB2 | RAB7B | KCNJ5 | IL17A | ECM2 | CD274 |
| GRP | TYRO3 | BCAM | GZMB | EML1 | CXCR6 |
| GRP | PLXDC1 | MC1R | SIRPG | FGF13 | BTLA |
| GRP | CARD6 | CCN3 | ST3GAL3 | GFER | CENPM |
| HSPA1A | IRF1 | NPR1 | CTLA4 | GNG7 | IRF4 |
| CCN3 | ZNF668 | NPR3 | FGF10 | GOLGA2 | IRF1 |
| NPR3 | SLC6A12 | NTSR1 | GPR25 | GRP | CHRNA5 |
| NPR3 | MFSD2B | NTSR1 | CD200R1 | GRP | PSPN |
| OLR1 | CXCR6 | SERPINE1 | MOB3A | GRP | CDC14A |
| RGS3 | C2CD4A | PTH1R | GTSE1-DT | GRP | CDK5R1 |
| RTN2 | SPOCK2 | RDX | MOCOS | GRP | ARHGAP25 |
| STXBP2 | CXCL2 | SGCA | UBASH3A | GRP | SPNS1 |
| TCHH | ZNF831 | SNCG | S1PR4 | GRP | FRMD6 |
| TRPC1 | RASGRP1 | SPP1 | SCO2 | GRP | PTGR2 |
| TRPS1 | UBASH3A | TAGLN | CTSK | HSPA1A | USP9X |
| VIP | IDO1 | TEAD1 | NEIL2 | HSPA1A | LSM1 |
| PCGF2 | CEP55 | TGFA | CD3D | HSPA1A | MCAT |
| DYRK3 | CXCR6 | CLDN5 | DOK2 | HSPA1A | APPL2 |
| SCEL | NRG1 | ZNF154 | TNFSF14 | HSPA1A | CDCA5 |
| NOG | CD226 | SCG2 | GBP5 | CCN3 | FBXL18 |
| ARMCX2 | CALHM6 | H2BC21 | EXOC6 | NPR1 | TBC1D10C |
| CAPN10 | C2CD4A | HERC3 | ERI1 | NPR3 | SLC16A7 |
| KLF12 | CTLA4 | BAIAP3 | IL12RB1 | NTSR1 | KCNN3 |
| KIFAP3 | RP2 | HAND2 | CD22 | SALL2 | TNFRSF9 |
| SLC4A1AP | ME2 | HAND2 | CEACAM4 | SALL2 | TIGIT |
| TMCC1 | C2CD4A | NR1D1 | UBA7 | SLC1A3 | SLAMF1 |
| ARC | GRIN2B | SOX13 | ZC3HAV1L | TPM2 | TRAP1 |
| ARC | ZBTB32 | UTP14C | SLC18B1 | TPM2 | NCBP2AS2 |
| TMEM59L | TPBGL | TMCC2 | TNFSF14 | TNFSF4 | CXCR6 |
| ANGPTL4 | ATPAF2 | LZTS1 | LAG3 | TYRP1 | ITK |
| GPRC5D | CXCR6 | RRAS2 | CD3D | SCG2 | ZBP1 |
| BEX4 | CCNI2 | LIMCH1 | CCL22 | H2BC21 | GNAI3 |
| FNDC4 | IDO1 | VIRMA | COQ2 | CAVIN2 | SLAMF7 |
| SCNM1 | CDC45 | RNF167 | GSR | CHST1 | CIITA |
| CRISPLD1 | CD274 | RAI14 | INTS9 | AOC3 | BIRC3 |
| CPT1C | ICOS | FGFR1OP2 | CD3D | ST3GAL5 | CXCR6 |
| UBE2QL1 | CEACAM3 | ANGPTL4 | CXCL11 | BAIAP3 | CD247 |
| NUDT17 | CXCR6 | ANGPTL4 | CALHM6 | NOG | TOP3B |
| ZNF561-AS1 | CIITA | ZNF692 | COQ2 | NOG | STOX2 |
| HLA-F-AS1 | ICOS | TMEM126B | GTF2E2 | QKI | CCL22 |
| IGFL1 | CD80 | BEX4 | MKS1 | HAND2 | LINC01555 |
| TMEM81 | IDO1 | GALNT16 | TIFAB | CHST3 | CCL22 |
| LOC101929704 | DNASE1L3 | PNMA8B | ALPK2 | RCAN2 | IL2RB |
| DDIT4L | CD1B | PDZD4 | GHRL | SMNDC1 | COQ2 |
| TCEAL2 | CYP4F22 | POLD4 | PARP3 | TDRKH | CD3E |
| HOTAIR | RNU5E-1 | GDPD3 | CD3D | KIFAP3 | RAVER2 |
| H2BC7 | LINC00996 | LY6G6C | PRKAR2B | HEY1 | IDO1 |
| ARL4D | PTPN7 | NPL | IL2RB | HEY2 | CIITA |
| NKX3-2 | LINC00996 | CRISPLD1 | CD3G | LAMP5 | GPR65 |
| CALB2 | GPR171 | NTNG2 | GPR25 | LAMP5 | SNAI3 |
| CAV2 | IL2RB | ZBTB46 | CCR7 | LMOD1 | HLA-DMB |
| ATF6B | GSR | ISM2 | CEACAM3 | TIAM2 | CD3G |
| CSF2RA | CXCR6 | ZNF569 | LINC00996 | CABYR | LINC01555 |
| EPOR | CXCR6 | ZNF570 | IL18R1 | C6orf15 | IL12RB1 |
| GPC1 | GBP1 | LCA5 | MEI1 | ANGPTL4 | NAT1 |
| GRP | POLI | ZNF385C | RASGRP1 | FAM178B | AKAP3 |
| GRP | RABGEF1 | ZNF385C | UBASH3A | YBEY | C2CD4A |
| GRP | XAF1 | STUM | ANK1 | TMCO1 | RNF19B |
| GRP | MAN1C1 | SMIM10L2B | TMEM266 | UFSP2 | COQ2 |
| GRP | TRIM16L | TMPO-AS1 | CXCR6 | TNFRSF19 | CD274 |
| GRP | ZNF805 | ZHX1-C8orf76 | SIRPG | PCDHGA2 | GIMAP5 |
| HSPA1A | KIAA0930 | ZNF528-AS1 | GPR18 | BEX4 | FYCO1 |
| HSPA1A | INTS10 | LINC02257 | NCR1 | BEX4 | NAA40 |
| CCN3 | APBB1IP | GNGT1 | MAL | JPH2 | FFAR2 |
| CCN3 | ALG9 | COL4A2-AS1 | CCR6 | CLSTN2 | NRG1 |
| CCN3 | BRIP1 | BAALC | CHRM3-AS2 | C11orf1 | C4orf19 |
| NPR3 | CCR3 | LINC00958 | RSPH4A | POGLUT2 | CCDC134 |
| NTSR1 | IFNG | HES7 | LGALS17A | C1orf116 | IRF1 |
| NTSR1 | CLEC12A | IGFL1 | PI16 | CCDC102B | RASGRP1 |
| SERPINE1 | APOL2 | NOG | CSNK1G2-AS1 | ST6GAL2 | TNFSF14 |
| SEPTIN5 | CXCR6 | BNIP3 | ARHGAP44 | ZNF587 | C2CD4A |
| PSMC6 | PBK | CA11 | CXCR6 | SNORD14E | CMC1 |
| SLC11A1 | MMP25 | CACNB3 | TNFRSF11A | GNRHR2 | LINC00114 |
| CLDN5 | IL2RB | CDH4 | ZNF831 | R3HDML | IDO1 |
| SCG2 | SIRPG | COL9A3 | CDCA2 | ISM2 | ARHGAP22 |
| H2BC21 | ZNF552 | MAPK14 | IRF1 | SPACA6 | ICOS |
| SUPT3H | CCL22 | DDIT3 | DLAT | SPICE1 | ERI1 |
| PPFIA4 | LOC100507144 | ERG | CD274 | RSPH9 | LINC00114 |
| DGKI | CLNK | GRP | CD36 | TCP11L2 | GFI1 |
| CABP1 | CD1B | GRP | LAMC3 | BEST4 | DNASE1L3 |
| CIR1 | XPO7 | GRP | LYG1 | ERFL | RASGRP1 |
| AKT3 | LAG3 | GRP | FGD2 | MBNL1-AS1 | RASGRP1 |
| CAP2 | CD247 | GRP | TPI1P2 | IBA57-DT | CASS4 |
| SEMA6C | CIITA | GRP | PCBP1-AS1 | SMIM10L2B | FASLG |
| SEMA6C | MMP25 | GRP | NCF1 | SMIM10L2B | DNAJC28 |
| SAMD4A | CD247 | HSPA1A | PSMD6 | MROH3P | GPR25 |
| FRMD4B | CCL22 | HSPA1A | SH2D4A | NOG | ARAP1-AS2 |
| CADM1 | SLAMF1 | IL1RAP | CD3G | CIART | GPR25 |
| HEYL | THEMIS2 | INHBB | PDE4B | MICU3 | CD226 |
| C6orf15 | LOC283922 | INHBB | PDLIM2 | GRP | LTA |
| EMCN | CTLA4 | KCNJ8 | CD8A | PRR29 | CD1B |
| SLC41A3 | LARS2 | KCNQ3 | ZNF831 | WFDC10B | SIRPG |
| ODR4 | APOL6 | LGALS8 | TNFRSF11A | NOG | RSPO1 |
| ADAP2 | CCL22 | CCN3 | TNFRSF17 | CDH2 | CCL25 |
| NIPAL3 | CD3D | CCN3 | ACAP1 | GRP | SLC6A7 |
| IGSF9 | KLHL18 | CCN3 | JAML | GRP | LINC02323 |
| GPBP1 | ATP6V1B2 | NTSR1 | TRHDE | NPR3 | ECE1-AS1 |
| SUGCT | LAG3 | NTSR1 | ENTPD1-AS1 | HCAR1 | FCRL5 |
| MZT2B | GSR | PDE2A | TIGIT | KCNK9 | IFNG |
| C3orf36 | LINC00996 | SMARCE1 | CD2 | LOC102724684 | BFSP2 |
| EGFL8 | LINC00114 | H2BC21 | MOCOS | SNORD53B | CARD17 |
| ZNF528 | SH2D1A | PPFIA4 | KIR2DL4 | TCEAL2 | CLNK |
| TSEN15 | IRF1 | ITGA10 | CHRM3-AS2 | NOG | BLK |
| SLX4IP | IL2RB | ASIC3 | GPR25 | TCEAL2 | LINC02273 |
| UBE2QL1 | TBX21 | AKAP12 | CD8A | SMCO3 | CHRM3-AS2 |
| DIPK1B | CXCR6 | CDIPT | LAP3 | SLITRK3 | CLNK |
| NIFK-AS1 | CXCR6 | ZNF268 | CD247 | ALDOC | GALK1 |
| TPRG1 | IL9R | RPIA | GSR | CAPS | CD3E |
| ZSCAN16-AS1 | CXCR6 | SUZ12 | C2CD4A | RUNX2 | CTLA4 |
| BOLA3-AS1 | LINC00114 | OPN3 | IDO1 | CDH2 | LOC100506585 |
| NOG | IL17A | PPP1R15A | LAP3 | CNN1 | ITGB2 |
| ADARB1 | IL12RB1 | C6orf15 | FAM169A | GSDME | CASS4 |
| ATF2 | CD3D | TPRKB | PBK | DTNA | KCNAB3 |
| EPS15 | COQ2 | PCDH12 | PTPN7 | DTNA | SLC47A1 |
| GAMT | DNASE1L3 | NKAPD1 | CD3D | FLT4 | DNASE1L3 |
| GRP | C4A | TNFRSF19 | HTR7P1 | FLT4 | CIITA |
| GRP | PRKCB | PIP4P2 | WRN | GRP | PCSK5 |
| GRP | REL | PPP4R3A | NOS2 | GRP | MOK |
| GRP | RAPGEF4 | ZNF83 | IDO1 | GRP | SLIT3 |
| GRP | NUDT6 | MBNL3 | EPHA10 | GRP | SIGLEC1 |
| GRP | SORCS2 | NDUFA4L2 | ODF3B | GRP | LINC00525 |
| GRP | PRRT1 | THOC2 | C2CD4A | GRP | PSMG3-AS1 |
| GRP | LINC01547 | ZNF608 | CCL22 | GRP | LEAP2 |
| GRP | DUSP19 | GPR107 | TYMS | GRP | PPM1K |
| INHBB | NFAM1 | KCNK15 | GPR25 | GRP | SERHL2 |
| CCN3 | RTTN | ZFAND1 | CASP1 | GRP | BEST4 |
| CCN3 | SLC22A15 | CSRNP2 | NUDT18 | GRP | MAP3K14-AS1 |
| CCN3 | ZNF407 | HVCN1 | SIRPG | HSPA1A | ACAA1 |
| NPR3 | SPATC1 | MSANTD4 | IL2RB | HSPA1A | MKKS |
| NPR3 | TAFA1 | LRRC46 | GPR25 | HSPA1A | MFHAS1 |
| PPP2R3A | CD3G | RBP7 | GBP5 | HSPA1A | SH3BP1 |
| PPP2R5B | CD3D | CD109 | SLAMF1 | HSPA1A | ELP6 |
| RBM4 | CCL22 | ZNF780B | CXCR6 | HSPA1A | METTL17 |
| ST3GAL3 | IL12RB1 | ANKRD24 | GPR25 | FOXN2 | CD2 |
| SP4 | SIRPG | TLCD5 | LINC00114 | INHBB | CLIC2 |
| PPFIA4 | UNQ6494 | TMEM105 | TMIGD2 | INHBB | CRYBB2P1 |
| LZTS1 | SIRPG | NOP14-AS1 | CXCR6 | INHBB | CAMK1 |
| GPR162 | RASGRP1 | BEX5 | GFI1 | INHBB | TNRC6B |
| RGCC | TNFAIP3 | BEX5 | PIK3R5 | INHBB | LRP5L |
| STK26 | CASP1 | LINC-PINT | TBC1D10C | KPNA1 | CD3D |
| MAP1S | NOS2 | SHISA2 | CXCR6 | MAP1B | CIITA |
| STRBP | COQ2 | GPX8 | CALHM6 | NEDD9 | CD3D |
| ZNF83 | F2RL2 | SMIM10L2B | NUGGC | CCN3 | MYO9A |
| TMIGD3 | LINC00996 | FAM155A | CD226 | CCN3 | ZNF112 |
| MEAK7 | IL2RB | SMIM10L1 | CD2 | CCN3 | ZNF774 |
| INIP | COQ2 | ZHX1-C8orf76 | ICOS | PFDN1 | SLC39A14 |
| MED28 | CD3D | SNCB | SAA4 | PLAG1 | FASLG |
| LINGO1 | GFI1 | SYT4 | CLNK | PPEF1 | CHRM3-AS2 |
| TMEM209 | CD3D | GRP | C11orf45 | PKIA | UBASH3A |
| SNORD14E | CCDC68 | ARHGAP6 | GPR25 | PTGER3 | GPR25 |
| ADHFE1 | CHRM3-AS2 | F13A1 | TBC1D10C | NECTIN2 | GSR |
| LAYN | DNASE1L3 | GRP | H2BC17 | RDX | DOK2 |
| LOC153684 | GPR25 | GRP | CATSPERB | SALL2 | LTA |
| SYNPO2 | CCR7 | HOXA4 | LINC00996 | SHC1 | SLC39A14 |
| LINC-PINT | DNASE1L3 | HOXC11 | LINC02332 | STXBP1 | TBC1D14 |
| TCHH | KIR2DL4 | HSPA1A | GSTM4 | TLE1 | NOS2 |
| TLL1 | CD226 | CCN3 | WDR7 | TMOD1 | GPR25 |
| IGFL1 | IGLL1 | RDX | CCL22 | UGCG | LEPROTL1 |
| ARC | H2AC14 | KLF7 | CCL22 | VIP | LAG3 |
| DUSP26 | IFNG | LRRC17 | UBASH3A | VLDLR | CD3G |
| TSLP | IFNG | LRCH1 | TNFRSF11A | ZNF212 | CD3D |
| LINC01655 | BASP1-AS1 | RGL1 | CCL22 | AKAP6 | GIMAP5 |
| GUCY1A2 | ZNF831 | TMEM245 | CD3D | ZSCAN12 | IL12RB1 |
| LMO2 | CXCR6 | UFSP2 | CD2 | SNAPC5 | CD3D |
| CCN3 | SEMA3B-AS1 | SNORD14E | ELAC1 | NXPH4 | TMIGD2 |
| TSPAN31 | CD2 | ARL14EP | FECH | ARC | DNAAF4 |
| H2BC8 | DNASE1L3 | AFAP1L1 | CIITA | ARC | LOC101928445 |
| KCNQ4 | CD244 | ISM1 | CD33 | POFUT2 | CD3D |
| RTN3 | CXCL3 | PLB1 | FASLG | ZFPM2 | TBX21 |
| TMOD2 | LINC00996 | PPM1K | SLA2 | TMEM59L | CABP7 |
| INKA2 | LINC00996 | BCLAF3 | CXCR6 | BRMS1 | RPS6KA1 |
| ZNF528 | ACKR4 | GXYLT1 | CCL22 | ELP4 | COQ2 |
| BEX5 | LINC00996 | RTL5 | CASS4 | RGCC | NECAP2 |
| GPX8 | IL2RB | BEX5 | IL21R | C6orf15 | AGAP1-IT1 |
| HOTAIR | CRLF2 | ZNF321P | CXCR6 | SHANK1 | CLNK |
| RGN | LGALS17A | APTR | TRIM69 | ANGPTL4 | TMEM229B |
| C5orf46 | P2RY10 | IGFL1 | TAFA1 | PAIP2 | GTF2E2 |
| CALB2 | CD1D | CRYBA4 | CD1B | THG1L | NOS2 |
| CALB2 | APOBEC3D | LINC01655 | TREML1 | CCDC91 | IL2RB |
| CALB2 | C8orf58 | NOG | SLC45A2 | TNFRSF19 | BMP5 |
| CDO1 | GPR18 | TMEM59L | CCR3 | UBE2Q1 | TNFRSF1B |
| GRP | CSF2RB | CACTIN-AS1 | GPR25 | LIMS2 | IDO1 |
| PLCG2 | CXCR6 | ZNF667 | CHRM3-AS2 | PCID2 | AGPAT5 |
| LAMP5 | CD69 | TYRP1 | CARD17 | PCDHGA1 | JAKMIP1 |
| LAMP5 | DOCK2 | TPTEP1 | CLNK | BEX4 | SNORA73B |
| ANGPTL4 | EMILIN2 | IGFL1 | LINC02332 | GJC2 | CTLA4 |
| BEX4 | UBIAD1 | LINC01213 | LINC01648 | ZNF512B | DNASE1L3 |
| BEX4 | SH3RF2 | ACTC1 | TNFRSF9 | FBRS | IRF1 |
| CCDC130 | CXCL2 | GRP | NINJ2 | CCDC136 | TNFSF14 |
| ZNF610 | FFAR2 | PALM | CALHM6 | DBNDD1 | CD2 |
| ANO5 | NRG1 | SOX11 | TNIP3 | C1orf35 | CD3D |
| TMEM105 | LAG3 | TPM2 | FASN | SRCIN1 | LAG3 |
| IGFL1 | MATK | VIP | FGL2 | JAM3 | IL2RB |
| IGFL1 | MEI1 | HSPB7 | SLAMF7 | SLC49A3 | CXCR6 |
| TPTEP1 | TRG-AS1 | HSPB7 | TSPAN11 | HINT2 | NOS2 |
| LINC02381 | CXCR6 | ANGPTL4 | NLRX1 | TCEAL3 | IGSF6 |
| PMF1-BGLAP | ADH6 | BEX4 | PSMD6 | PPP1R14A | ODF3B |
| MYL4 | CD1B | BEX4 | TRAK2 | CYTOR | IL2RB |
| NGF | RPS2P32 | ARMH4 | CD274 | C1QTNF3 | TBC1D10C |
| MAMDC2 | TBX21 | MLKL | COQ2 | FCRLB | LINC00996 |
| TPRG1 | IFNG | MUC20 | EPHB2 | C1orf131 | CD3E |
| C20orf202 | CD244 | CBY2 | CXCR6 | CD109 | CD3G |
| PRCD | CHRM3-AS2 | LOC339803 | SLAMF1 | PNCK | ZMYND10 |
| LY6H | ACKR4 | ZNF284 | CXCR6 | ZBTB46 | IL12RB1 |
| DLX5 | CLNK | LOC102723566 | CXCR6 | TMEM86A | TBC1D10C |
| GRIK2 | CHRM3-AS2 | OMD | IL17A | SPC24 | C2CD4A |
| GRP | APOBEC3H | NOG | ABCD2 | NBPF11 | SIRPG |
| PRG4 | CLNK | PCP4L1 | IPO9-AS1 | ZNF615 | CXCR6 |
| PNMA8A | SLAMF1 | GABRB2 | CDRT15 | CRB2 | DNASE1L3 |
| PRSS35 | LINC01915 | CAMK2B | MCF2L2 | PRSS53 | MEFV |
| GAP43 | TRG-AS1 | CD72 | CTLA4 | BEX5 | GBP5 |
| GRP | LINC01273 | CRYAB | FAM83F | BEX5 | ANKDD1B |
| NTSR1 | IL12RB2 | DTNA | USP3-AS1 | STUM | IL26 |
| PRG4 | LINC02273 | GRP | CSTA | NPIPB4 | GPR25 |
| ZNF667 | CLNK | GRP | EGR2 | HOXA-AS3 | NRG1 |
| FGF19 | PRSS1 | GRP | DLC1 | ALMS1-IT1 | GPR25 |
| NKX3-2 | CCR4 | GRP | SLC49A3 | LOC101929709 | UBASH3A |
| CACNB3 | C2CD4A | GRP | LAYN | ZNF528-AS1 | NRG1 |
| CALB2 | BTK | GRP | ECSCR | VAX2 | JAKMIP1 |
| CALB2 | IL16 | GTF2F2 | MMP12 | ACOXL | IL17A |
| DDIT3 | FUT2 | HOXC4 | PYHIN1 | NALCN | FAM163A |
| ENO2 | NLRC5 | CCN3 | MCPH1 | C5orf46 | FASLG |
| ENO2 | AP5B1 | PSD | CIITA | LINC01655 | LY9 |
| GRP | HLX | RGS16 | CXCL10 | CCDC181 | LINC02273 |
| GRP | MPHOSPH9 | SGCA | P2RY13 | SEMA3D | IFNG |
| GRP | ARHGEF15 | MAP3K7 | COQ2 | NOG | PFKFB1 |
| GRP | APBB1IP | PPFIA4 | THEMIS | PCDHB6 | CHRM3-AS2 |
| GRP | CERS4 | AOC3 | CSF1 | IGFL1 | CD22 |
| GRP | LINC00888 | LPGAT1 | IRF1 | DLX5 | LHX2 |
| MRPL58 | GSR | LPAR6 | FAS | ALOX15 | H3C7 |
| CCN3 | IDO1 | SEMA6C | CCR7 | CALB2 | GPR25 |
| CCN3 | ZNF607 | ARC | AKAP3 | CLU | CD2 |
| NTSR1 | LTA | NOX4 | TIGIT | CRMP1 | SIRPG |
| SERPINE1 | OAS3 | HIGD1B | GBP5 | DBP | CXCR6 |
| PLAG1 | CLEC2D | BEX4 | MTFR1L | DDIT3 | EXOSC10 |
| PLAG1 | RIPOR3 | SHD | CALHM6 | ETV5 | CCL22 |
| RGS16 | APOL6 | IGSF9 | CD3D | FABP3 | IL2RB |
| TSPAN31 | PAFAH2 | COLEC12 | GFI1 | FER | ICOS |
| SLC14A1 | FASLG | CYRIA | CXCR6 | GRP | CD244 |
| SPP1 | DRG1 | WDR83 | C2CD4A | GRP | ZNF699 |
| UBE2E2 | IDO1 | HOPX | CCL22 | HNMT | CHMP7 |
| PCGF2 | IRF1 | CADPS2 | C2CD4A | HSPA1A | TAP2 |
| PPFIA4 | GPR25 | UHRF2 | C2CD4A | HSPA1A | GMPPB |
| MTA2 | GSR | UBE2QL1 | IL18RAP | INHBB | DLG4 |
| HAND2 | TLR8 | UBE2QL1 | LOC100506585 | BCAM | NUDT18 |
| HAND2 | LINC00996 | PNCK | CD244 | NTSR1 | ANO2 |
| FEZ1 | IL12RB1 | R3HDML | IL2RB | PHKG1 | GPR25 |
| GPRASP1 | IRF4 | ZNF169 | CXCR6 | PTH1R | LGALS17A |
| GPRASP1 | SLA2 | GRID2IP | SIRPG | PURB | NEIL2 |
| LRRC17 | GBP5 | SPRED3 | TNFSF14 | SIAH1 | CCL22 |
| NMU | CXCL9 | SMIM10L2B | TNFSF14 | SQLE | SPNS2 |
| SF3B2 | LAP3 | NBPF10 | IL17A | SEC62 | XPO7 |
| TGDS | AGPAT5 | MAGI2-AS3 | TNFRSF9 | TMOD1 | TBX21 |
| RAI14 | SOCS6 | IGFL1 | GPR171 | VLDLR | TIGIT |
| CYTH4 | CCL22 | GAP43 | GPR25 | SPARCL1 | CXCL10 |
| C6orf15 | CTLA4 | PPEF1 | IL17A | MTA2 | LAP3 |
| RAB9B | LINC01555 | CDK5R2 | CHRM3-AS2 | RB1CC1 | CD3D |
| PALMD | P2RY10 | HAND2 | NCF1B | HDAC5 | PI4KA |
| SLC41A3 | LETM1 | CCBE1 | CD226 | GJC1 | GPR171 |
| PI4K2A | COQ2 | TCHH | IFNG | FST | TNFRSF9 |
| LIN37 | CXCR6 | ZFHX4-AS1 | BFSP2 | SEMA6C | CD3G |
| BEX4 | MORC4 | NUDT10 | IL22RA2 | TRAPPC2B | DNASE1L3 |
| PDZD4 | LINC00996 | NGF | BTLA | STMN2 | ICOS |
| FAM3A | CXCL3 | CACNB3 | MAST3 | KIFAP3 | CD3D |
| CDADC1 | ERI1 | CACNB3 | EXOC6 | KIFAP3 | ME2 |
| HMCN1 | ICOS | CALB2 | CCR4 | ZFPM2 | CARD17 |
| ZNF573 | UBASH3A | CD79B | CXCR6 | LMOD1 | IDO1 |
| ARMH4 | LINC00114 | S1PR3 | CXCL13 | RAI14 | CALHM6 |
| DACT3 | CIITA | ETV3 | C2CD4A | SLC45A1 | GPR25 |
| CAMSAP1 | C2CD4A | GRP | FBXL7 | MPHOSPH8 | ATP6V1B2 |
| FAAP20 | C2CD4A | GRP | GDPD2 | ARMC1 | CASP1 |
| NBPF11 | CXCR6 | GRP | ZCWPW1 | TTC17 | NEIL2 |
| SHISA2 | IDO1 | GRP | SLC38A6 | PCDHGA1 | KIR2DL4 |
| TMEM200B | CXCR6 | GRP | LOC100129917 | MOSPD1 | CD2 |
| NBPF9 | CXCR6 | HSPA1A | R3HCC1 | TMIGD3 | FASLG |
| ZBTB34 | IL2RB | INHBB | GIT2 | ZNF512B | IKZF1 |
| MIR4435-2HG | IL2RB | MAP3K11 | CENPM | NDRG4 | GPR25 |
| SMIM10L2B | FBXW4P1 | MPP2 | IL18RAP | PRR14 | NOS2 |
| MINCR | C2CD4A | CCN3 | CD8A | NIPAL2 | ABHD3 |
| CDO1 | TBX21 | CCN3 | ZNF417 | ZNF606 | TMIGD2 |
| CDK5R2 | IL9R | CCN3 | TSPAN11 | SH3BP5L | NUDT18 |
| HAPLN2 | LHX2 | NTSR1 | SLC6A12 | ZNF816 | CALHM6 |
| LINC01686 | KIR2DL4 | SERPINE1 | SPSB1 | GIPC3 | SLAMF1 |
| LINC02257 | IL26 | PDE6D | COQ2 | WTIP | GPR25 |
| HOXC4 | BTLA | PGM5 | GFI1 | ISM1 | LTA |
| IGFL1 | MEFV | RYR2 | IFNG | ISM1 | CCL23 |
| SLC25A30-AS1 | FASLG | SLC2A3 | GBP4 | ISM1 | NCR3 |
| IGFL1 | GRK1 | SPP1 | ADAM9 | VSIG10L | TNFRSF9 |
| RNF112 | IL9R | UBE2E2 | CXCL13 | PRICKLE2 | TIGIT |
| CORO6 | CHRM3-AS2 | ZNF26 | MMP25 | TLCD5 | RASGRP1 |
| CALB2 | CYP26B1 | H2BC21 | RCHY1 | PHYHD1 | RASGRP1 |
| CDH4 | KIR2DL4 | AOC3 | THNSL2 | ZNF530 | LINC00996 |
| COMP | HLA-DOA | MPDZ | ICOS | LINC-PINT | MMP25 |
| CST2 | HIC1 | DIRAS3 | GPR18 | TMEM233 | CHRM3-AS2 |
| DDIT3 | NT5C3A | CRLF1 | SLAMF1 | SMIM10 | MMP25 |
| TSC22D3 | CXCL10 | HAND2 | ADAM23 | PMF1-BGLAP | LAIR2 |
| GAS1 | GBP5 | SH3BP5 | CXCR6 | ZNF528-AS1 | IFNG |
| GRP | HSPA1L | SLC12A6 | IL2RB | ZNF528-AS1 | TRG-AS1 |
| GRP | ARHGAP8 | LRRC17 | SIRPG | ZNF229 | CHRM3-AS2 |
| GRP | LRRC29 | EFS | CXCR6 | C3orf49 | CHRM3-AS2 |
| GRP | HERC5 | LZTS1 | CD247 | IGFL1 | DBH-AS1 |
| GRP | RAPGEF6 | ARC | MFF-DT | HOTAIR | LINC02332 |
| GRP | ALKBH6 | KIAA1549L | IL17A | NOG | KLHL41 |
| GRP | PEAR1 | POT1 | CD2 | CRYAB | CCNI2 |
| GRP | NAPA-AS1 | TUBG2 | CD3E | GRP | NAT2 |
| HSPA1A | TBP | C6orf15 | ULK4 | GRP | FNTB |
| CCN3 | CCR5 | G0S2 | TYMP | GRP | FBXO25 |
| NPR3 | SAMD3 | DACT1 | IL2RB | THBS4 | F2RL2 |
| PIM1 | NCOA7 | ZCWPW1 | CIITA | PPFIA4 | RGL4 |
| PRELP | CD300LF | EVA1B | NOS2 | ARC | LINC00114 |
| PTH1R | NUGGC | DHRS4-AS1 | COQ2 | LAMP5 | IL2RA |
| THBS4 | CCR4 | TNFRSF19 | SAA2 | LAMP5 | LMLN |
| TPM2 | TNFRSF1B | PIP4P2 | SEC22C | ANGPTL4 | IL27RA |
| UCHL1 | CCR7 | BEX4 | SOCS1 | SCN3B | XCR1 |
| H2BC21 | TNFRSF11A | NDUFA4L2 | HLA-DMB | BEX4 | SNX14 |
| AOC3 | SPOCK2 | ADAMTSL3 | TRG-AS1 | NKAIN4 | P2RY10 |
| JMJD7-PLA2G4B | LINC00114 | ZNF250 | IDO1 | STUM | RASGRP1 |
| SH3BP5 | IL12RB1 | FBRS | CXCL2 | TMEM200B | CCL22 |
| CHST10 | ICOS | GAL3ST4 | CD3E | INHBB | PSTPIP1 |
| CIR1 | ATP6V1B2 | ZNF408 | COQ2 | DLK2 | GPR25 |
| SOX13 | FOXA1 | PREX2 | PYHIN1 | RUNDC3B | CHRM3-AS2 |
| ARMCX2 | IDO1 | RNASEH2C | PBK | PNMA6A | IL17A |
| ABCC9 | TESPA1 | CORO6 | TRG-AS1 | NOG | SH3GL2 |
| LDB3 | CHRM3-AS2 | BMERB1 | IDO1 | CHRM4 | CD1B |
| ENPP4 | C2CD4A | METTL18 | GZMA | NALCN | LINC01281 |
| WDR47 | IL2RB | AHNAK2 | CD247 | CALB2 | CTSW |
| NACAD | TNFSF14 | PAQR4 | CENPM | CALB2 | GZMH |
| HEYL | CD3E | UBE2QL1 | LILRA1 | CALB2 | PPP1R16B |
| INTS6 | CCL22 | PNCK | KIF19 | CALB2 | FLVCR2 |
| RGCC | IRF1 | PSORS1C1 | CIITA | CALB2 | P2RY8 |
| KIF26B | CD274 | PGBD2 | IL2RB | COL8A2 | IL2RB |
| TNFRSF19 | AIM2 | NEK8 | EPHA10 | DCTD | GSR |
| TNFRSF19 | VAT1L | DUBR | CASS4 | AKR1C2 | SLA2 |
| TNFRSF19 | LYG1 | WASH2P | IL12RB1 | EGR2 | IDO1 |
| BEX4 | UBXN11 | IGIP | IL2RB | EGR2 | FOXP3 |
| GALNT16 | ZNF831 | HHIP-AS1 | TNFRSF9 | FKBP2 | GSR |
| HAMP | TIFAB | SPATA1 | CHRM3-AS2 | GGTA1P | SLAMF1 |
| DPEP2 | TNFRSF9 | LOC101927851 | PDCD1 | GNGT1 | FASLG |
| HSPBAP1 | CCL22 | IGFL1 | FBLL1 | GRP | ZEB1 |
| FBXO11 | CD3E | NOG | FGF7P6 | GRP | PDE5A |
| LY6G6C | WFDC21P | LY6H | IFNG | GRP | FCMR |
| GLIS2 | CALHM6 | IGFL1 | C12orf74 | GRP | SASH1 |
| SNORD14E | SIT1 | METTL24 | IFNG | GRP | OSBP2 |
| TNKS1BP1 | REEP4 | NOG | AMPD1 | GRP | ZNF324 |
| CAVIN3 | NOS2 | RGS6 | KLRC1 | GRP | PAMR1 |
| ZNF441 | CXCR6 | NOG | U2AF1L5 | GRP | ZNF668 |
| ZBTB46 | LAG3 | LINC01655 | DDC-AS1 | GRP | SHISA2 |
| TMEM86A | CXCR6 | NOG | ASMT | HOXC9 | KCNG2 |
| UNC5B | CD3D | PRKAA2 | CCR6 | KIF5A | NRG1 |
| CALHM5 | TNFRSF9 | SCN5A | CCR6 | MATN3 | CXCL13 |
| PHYHD1 | SLA2 | LOC441179 | LAIR2 | MPZ | GBP5 |
| TOLLIP-AS1 | CXCR6 | ARL4D | CTSW | CCN3 | C4orf33 |
| COL24A1 | CCR3 | NKX3-2 | SUCNR1 | NPR3 | DNAJC28 |
| TTC28-AS1 | GFI1 | CDH4 | LHX2 | NPR3 | CEP85L |
| RXFP4 | IL2RB | CSRP2 | DNASE1L3 | NTSR1 | TNFRSF9 |
| ELFN1 | MMP25 | AKR1C1 | GHRL | PLAG1 | CD3G |
| NALCN | LINC02694 | GSDME | GPR25 | PRELP | GAS7 |
| MYO1H | LINC02273 | DTNA | IFNG | RAB3IL1 | CD2 |
| MYL4 | IL17A | DTNA | SMG1P5 | SNCG | TNFRSF18 |
| ADARB1 | CTLA4 | DTNA | LOC105377623 | THBS4 | CTLA4 |
| CACNB3 | ABI3 | FCGR2A | CALHM6 | TMOD1 | ICOS |
| CTF1 | ICOS | GRP | NFATC1 | TPM2 | APEH |
| DTNA | HPSE2 | GRP | SLC15A2 | SCG2 | PTPN7 |
| GRP | RAD54B | GRP | SACS | PEX11B | SLC39A14 |
| GRP | ZNF286A | GRP | ATP10A | NOG | SLFNL1 |
| GRP | BTBD11 | GRP | IQCD | TNIK | C2CD4A |
| GRP | CEP128 | GRP | UGDH-AS1 | SPART | IDO1 |
| HSPA1A | SUV39H1 | GRP | LOC101927751 | LAMP5 | IKZF3 |
| KCNC3 | GPR25 | GTF2F2 | CENPM | ANAPC13 | ASAH1 |
| KCNK1 | C2CD4A | HOXD8 | CXCR6 | ZNF521 | CXCR6 |
| MTR | TRIM69 | HSPA1A | UMPS | HSPB7 | APBB1IP |
| GADD45B | APOL6 | HSPA1A | PTPMT1 | NGRN | ME2 |
| CCN3 | LILRB1 | IL1RAP | CD274 | PIP4P2 | CXCL9 |
| CCN3 | ASB2 | INHBB | RCSD1 | PIP4P2 | MPEG1 |
| ITSN1 | CD247 | INHBB | ATP9B | BEX4 | RAB28 |
| ST3GAL3 | CD247 | LMO7 | ERAP1 | BEX4 | R3HDM1 |
| TBXA2R | ICOS | MAP1B | IKZF1 | MAGEE1 | MMP25 |
| THBS4 | LINC00996 | NAB1 | LEPROTL1 | ARHGAP10 | CTLA4 |
| ZNF75A | SIRPG | NAB1 | SH2D4A | EEPD1 | BCAT2 |
| TUSC3 | CTLA4 | CCN3 | POLI | CRISPLD1 | GBP5 |
| PPFIA4 | POLN | CCN3 | ZNF501 | MAGT1 | GSR |
| PPFIA4 | LGALS17A | CCN3 | P2RY8 | IGSF21 | UBASH3A |
| PPFIA4 | TIFAB | CCN3 | RAB37 | PNMA6A | NRG1 |
| NRP2 | S1PR4 | NTSR1 | MIXL1 | UBE2QL1 | TNFSF14 |
| NR1I3 | LGALS17A | PHF1 | APOL6 | ZNF383 | IDO1 |
| AKT3 | PDCD1 | PLAG1 | COL23A1 | KLHDC8B | GZMB |
| TANK | CD2 | RIT1 | CD2 | NHLRC4 | MEI1 |
| ABCC9 | EOMES | SPAG4 | CALHM6 | C5orf46 | CD80 |
| LPAR6 | SLAMF7 | TESK1 | NOS2 | SMIM10L2B | CASS4 |
| SLU7 | APOL6 | TUBB2A | CD3D | NOG | GRM2 |
| LEMD3 | ME2 | UCHL1 | GBP5 | ISM2 | ADGRE3 |
| WDR91 | CXCR6 | ZSCAN26 | IL2RB | TCTEX1D1 | ZNF831 |
| HDAC7 | CD3D | MTMR2 | CD3D | TRPC4 | IFNG |
| EGLN1 | FOXA1 | ITGB1BP1 | CD3D | IQCA1 | LINC02273 |
| ASPN | IL2RB | SOX13 | FAM193A | P2RX5 | GPR25 |
| RNF121 | CD3D | UTP14C | ME2 | HAP1 | LIPC |
| ZNF407 | SIRPG | FAM20B | LEPROTL1 | CUBN | CHRM3-AS2 |
| TRPV4 | TIGIT | UST | CD244 | TSPAN10 | CD244 |
| NAA35 | CCNI2 | PRG4 | ARRDC5 | IGFL1 | DNASE1L3 |
| DLK2 | ICOS | CBX6 | CALHM6 | NOG | LY9 |
| MSANTD2 | CCL22 | LAMP5 | SP140 | NOG | SLC35G5 |
| HMCN1 | CD1E | LAMP5 | BHLHE22 | ACTC1 | GVINP1 |
| NEXN | CIITA | NGDN | CD3D | ATP2B4 | C2CD4A |
| RBM18 | CD3D | PDLIM3 | CD8A | CACNA2D1 | RASGRP1 |
| RBP7 | CD274 | ZNF354C | NUGGC | CACNB3 | SOWAHB |
| AFAP1L1 | CTLA4 | PNPLA8 | CD3D | CALB2 | GPR3 |
| ZNF570 | BHLHE22 | GFOD1 | GFI1 | CALB2 | IRF4 |
| TTLL11 | DNASE1L3 | IMPAD1 | CASP1 | CALB2 | IKZF3 |
| BEX5 | CD101 | FKBP14 | IL2RB | CALB2 | GIMAP1 |
| TMEM59L | HDC | HEATR1 | ME2 | DPYSL3 | CXCL10 |
| TCEAL2 | KLRD1 | PCDHGB7 | GPR25 | DTNA | APOBEC3H |
| PPP1R1A | CHRM3-AS2 | NAA16 | ERI1 | EFNB3 | SLAMF1 |
| HAMP | CCL25 | SNORD14E | FAAP24 | ERG | LAG3 |
| IGFL1 | VIM-AS1 | ZNF251 | CFB | GRP | GLI1 |
| PRG4 | CLECL1 | TMEM150A | CD3D | GRP | KIT |
| CAMK2B | GPR31 | NEK7 | COQ2 | GRP | TMEM106A |
| CCN3 | LINC00114 | FUT11 | DOK2 | GRP | ARSK |
| NPR3 | NCR1 | FUT11 | SLAMF7 | GRP | CERKL |
| SOX11 | LINC01281 | FAM13C | TBX21 | GRP | CHKB-DT |
| MED20 | NEIL2 | MMS22L | CD3G | HSPA1A | LETM1 |
| ZNF432 | CXCR6 | SUGT1P3 | TIGIT | CCN3 | TOX |
| TMEM59L | CHRM3-AS2 | RAB7B | ICOS | CCN3 | RCSD1 |
| NGF | IL18RAP | RTL5 | LINC00996 | NTSR1 | DNAH2 |
| LINC02257 | IL9R | BEX5 | FFAR2 | NTSR1 | MFF-DT |
| AGTR1 | KLRC1 | GPX8 | FECH | NTSR1 | LOC100130691 |
| CAMK2B | IL26 | ZNF865 | SOCS1 | NTSR1 | SMG1P3 |
| CYP1B1 | CD3G | NAP1L2 | LINC00861 | SERPINE1 | ANKRD52 |
| GAP43 | TLR8 | LINC02257 | TBX21 | PLAG1 | RASGRP1 |
| GRP | FGR | LINC02257 | TRG-AS1 | PRKAB2 | C2CD4A |
| MAP6 | LINC00996 | LINC01655 | DNAJB13 | PSD | FFAR2 |
| SEPTIN4 | CXCR6 | GNGT1 | LOC105377623 | PTH1R | SLC6A12 |
| PTGIS | FOXP3 | NALCN | GATA1 | SCN1B | FFAR2 |
| RAB3IL1 | CD3D | LINC02257 | LINC00528 | SFRP2 | CXCL10 |
| SCN1B | CTLA4 | LOC339874 | CHRM3-AS2 | SLIT3 | CTLA4 |
| THBS4 | CCR7 | BCHE | C2orf74 | SPP1 | ACTL6A |
| HAND2 | PIK3CG | AVPR2 | CHRM3-AS2 | SPP1 | PPP2CA |
| HAND2 | ZNF671 | LPAR1 | CXCR6 | SPP1 | C4orf3 |
| ARMCX2 | CD3E | GRP | CD80 | ELOVL4 | HDC |
| LPAR6 | CD2 | GRP | PLD4 | TACR2 | POU2AF1 |
| CBX1 | ASAH1 | GUCY1B1 | CCL22 | TAF6 | CENPM |
| KIFAP3 | PEX7 | INHBB | TNFRSF10C | ELOC | IRF1 |
| KIFAP3 | PCGF5 | KTN1 | APOL6 | CLDN5 | CALHM6 |
| HSPB7 | ZDHHC14 | PLAG1 | TRG-AS1 | WNT7B | IL17A |
| BEX4 | MTMR1 | PLN | CD274 | ZNF66 | UBASH3A |
| BEX4 | RMC1 | RDX | RASAL3 | ZNF225 | SLA2 |
| BEX4 | LRIG3 | THBS4 | GVINP1 | H2BC21 | KCTD9 |
| TMIGD3 | SLA2 | NR1D1 | STX18 | CAVIN2 | IDO1 |
| JAM2 | CXCR6 | GPRC5B | CD247 | GAS7 | IDO1 |
| LRRC27 | CXCR6 | FRMD4A | SLAMF1 | SNUPN | CD3D |
| CRISPLD1 | CTLA4 | ADAP2 | CXCR6 | PRG4 | KLRD1 |
| TSPYL5 | CXCR6 | MOSPD1 | CD3E | B4GAT1 | MCAT |
| P4HA3 | CXCR6 | JCAD | GBP5 | ADAMTS6 | TNFSF14 |
| IGFL1 | UBASH3A | FAM234B | NCR3 | DUSP10 | C2CD4A |
| IGFL1 | MKX | POPDC2 | TIGIT | DZIP1 | SLAMF1 |
| NBDY | SLC39A14 | MIR503HG | GPR25 | LMOD1 | ABI3 |
| ZNF667-AS1 | CD3G | SNORD14E | CD8A | HEYL | ENTPD7 |
| FLJ31356 | CLECL1 | ADGRF5 | CCL22 | HCAR1 | ITK |
| SUN3 | CLNK | BEX5 | UBASH3A | HDGFL3 | MMP25 |
| GRIK2 | LHX2 | ANKRD65 | GPR25 | ANGPTL4 | ERI1 |
| SLC35F1 | NUGGC | FAM229B | SLAMF1 | PCDH12 | CCL22 |
| GRP | CCL19 | LAMP5 | SOWAHD | AIG1 | COQ2 |
| NPR3 | IL26 | NOG | MBL1P | TM6SF1 | TIGIT |
| PPEF1 | LGALS17A | NOG | CLEC4D | FXYD6 | CCL22 |
| C7 | UGT2B7 | TRIM58 | TIFAB | A4GALT | GBP4 |
| CALB2 | TNF | PCP4L1 | RSPH4A | PCID2 | IRF1 |
| DHX8 | PARP3 | LY6H | TNIP3 | NRIP3 | P2RY10 |
| FOXC2 | LINC00996 | RADIL | CD1B | PNMA8B | CAMK4 |
| FLT4 | CCR7 | NALCN | LILRP2 | RRAGD | CXCR6 |
| GEM | CXCL10 | GRP | ALOX15B | TSPAN10 | TRG-AS1 |
| GJA4 | C2CD4A | GRP | LOC105376527 | FCHSD1 | FAM83F |
| GPX3 | CXCL10 | NOG | SLC1A2 | FBXO27 | GHRL |
| GRP | LINS1 | KCNK15 | LINC01915 | UBE2QL1 | C10orf55 |
| GRP | CPNE5 | GRIK2 | BFSP2 | ISM1 | MFAP3L |
| GRP | ARAP2 | GRP | ADAMTS15 | FAM13C | GHRL |
| GRP | TAGAP | RNF112 | CD1B | JAZF1 | FOXP3 |
| GRP | E2F7 | SOX11 | ACKR4 | IGFL1 | FMO1 |
| GRP | ZNF597 | RFX8 | CLECL1 | IGFL1 | TSSC2 |
| GRP | ATP9B | CAMK2B | DTHD1 | ZNF805 | CXCR6 |
| INHBB | NOCT | C1QTNF1-AS1 | GPR25 | SPRED3 | GPR18 |
| KCNJ5 | TBX21 | FCGR1CP | CD1B | STX17-AS1 | CXCR6 |
| MAP6 | NRG1 | S1PR3 | CD3G | LOC100506083 | ICOS |
| MPO | CHRM3-AS2 | GRP | MYO1F | BOLA3-AS1 | ICOS |
| PPP1R12B | IL2RB | GRP | PTPRC | PMF1-BGLAP | CD274 |
| NPR3 | CFAP70 | GRP | HEATR5A | RAB6D | MTNR1A |
| NPR3 | JAKMIP1 | GRP | GINS3 | SLC25A34-AS1 | CCL25 |
| NTSR1 | TESPA1 | ANOS1 | CCL22 | CATSPER1 | NCR3 |
| SERPINE1 | ATP6V1B2 | NPR3 | ATE1-AS1 | PRRT4 | SAA4 |
| PDK4 | CD2 | GPANK1 | COQ2 | ST6GAL2 | H2BC10 |
| PTGIS | CXCR6 | DGKI | CHRM3-AS2 | NOG | CNR2 |
| RARB | CTLA4 | GFPT2 | FOXP3 | HAPLN2 | BFSP2 |
| RFX2 | CXCR6 | TMEM59L | FBXW4P1 | GRP | VPREB3 |
| SLC11A1 | CXCR6 | TIAM2 | CTLA4 | SUSD5 | NUGGC |
| SOX11 | IL12RB2 | SCN3B | CD226 | UBE2QL1 | GPR174 |
| SPP1 | TYROBP | BEX4 | SACM1L | PPP1R1C | KIR2DL4 |
| SPP1 | UBE2D3 | BEX4 | IL20RA | KANK4 | C2orf74 |
| SPP1 | GUCD1 | UBE2QL1 | INSC | IGFL1 | THEMIS |
| SPP1 | CENPW | ZNF785 | FAM83F | RASA4 | CLNK |
| TPBG | MOCOS | BNIPL | ICOS | MCOLN3 | CCR3 |
| TRPC1 | BHLHE22 | SLC46A3 | CXCL2 | ZNF471 | CHRM3-AS2 |
| RNF112 | TRG-AS1 | AMIGO2 | LPCAT1 | HOTAIR | LINC02273 |
| PPFIA4 | MEFV | IGFL1 | GPR65 | AR | ZNF831 |
| NRP1 | CD3E | ZNF582-AS1 | GHRL | CACNB3 | ANKFY1 |
| PNMA2 | TNFRSF9 | NOG | TCP10L2 | CACNB3 | LYRM2 |
| KIFAP3 | GCA | IGFL1 | HRH2 | COL11A2 | ADGRE4P |
| LAMP5 | SALL1 | NALCN | CR1L | CRYAB | LCP2 |
| LAMP5 | SLC28A3 | FAM124B | GPR25 | DTNA | KCNA3 |
| LAMP5 | PDCD1LG2 | ZNF542P | RASGRP1 | FOXC1 | UGT2B7 |
| TMEM59L | IL17A | MAMDC2 | LOC105377623 | GGT5 | CALHM6 |
| HSPB8 | IGSF6 | LINC02257 | SAMD3 | GPC1 | SOCS1 |
| RGCC | UBE3C | ADGRB1 | CD1B | GRP | CACNA2D2 |
| C6orf15 | CD247 | CALB2 | CD72 | GRP | ASB16 |
| REEP2 | ICOS | CALB2 | PI4KAP1 | GRP | FBXO36 |
| JPH2 | MEOX1 | F8 | SLA2 | GRP | ZNF283 |
| CBX8 | C2CD4A | FABP4 | SLAMF1 | GRP | SEMA3B-AS1 |
| FAM110D | CIITA | GRP | NCKAP1L | GUCY1A2 | GIMAP5 |
| TRIM11 | CCNI2 | GRP | TLE4 | HOXB4 | TRPA1 |
| ARHGEF25 | IDO1 | GRP | NEXN | HSPA1A | GINS1 |
| RBP7 | PTPN7 | HSPA1A | CNNM3 | HSPA1A | FARS2 |
| EDARADD | IL17A | HSPA1A | CCAR2 | HSPA1A | DCTN6 |
| PNCK | IL18RAP | INHBB | CASZ1 | HSPA1A | EARS2 |
| PNCK | LINC00996 | MMP17 | CXCR6 | HSPA1A | QSOX2 |
| ISM1 | RIPOR3 | MSR1 | CCL22 | INHBB | SMARCD3 |
| ISM1 | WFDC21P | NAB1 | YES1 | INHBB | CARD8-AS1 |
| TLCD5 | SLA2 | CCN3 | HEMK1 | MFAP3 | ERI1 |
| ADGRF5 | ERI1 | NPTX1 | CHRM3-AS2 | GADD45B | GBP2 |
| MAMSTR | TBC1D10C | NTSR1 | LAX1 | GADD45B | DNAJC11 |
| BEX5 | WNT4 | PLN | F2RL2 | NGF | TESPA1 |
| WDR53 | CD3D | PSD | GBP5 | NTSR1 | CAMK4 |
| SMIM10L2B | PTGDR | SOX11 | CYSLTR2 | NTSR1 | ACKR4 |
| LOC101929340 | LINC00114 | SPP1 | RAB2A | OLR1 | SIRPG |
| MYOSLID | IL9R | SPP1 | UBE2L6 | PHF2 | TMEM102 |
| ST6GAL2 | CHRM3-AS2 | SPP1 | PHF5A | PLOD2 | COX10 |
| LINC01561 | IL17A | TRPS1 | BHLHE22 | PYGM | JAKMIP1 |
| EGR4 | LGALS17A | TRPS1 | TIGIT | SGCA | LAIR2 |
| ADPRH | CCL22 | RND2 | CD1B | SNAI1 | SASH3 |
| ETV4 | GSR | HAND2 | RTN1 | SNAPC2 | NOS2 |
| FOXC2 | GVINP1 | EDAR | CD3E | TGFB2 | UBASH3A |
| GRP | PLA2G4C | EDAR | LCK | TRPS1 | IL18RAP |
| GRP | SLC4A4 | ARC | KLRG1 | TYRP1 | GPR25 |
| GRP | PTGDR2 | LAMP5 | FGD2 | VEGFC | TBC1D10C |
| GRP | LINC00869 | C6orf15 | CXCR6 | VIP | IL12RB1 |
| GRP | NEURL3 | CCDC88A | CD3G | VIP | PRKAR2B |
| GRP | TCEANC | PBXIP1 | SLC39A14 | ZNF121 | CD3D |
| GRP | TCP11L2 | RIMKLB | IRF4 | ZNF133 | CDCA2 |
| GRP | ZNF429 | SRPRB | LAP3 | ZNF225 | UBASH3A |
| GRP | CENPP | LYNX1 | TNFRSF9 | ALDH5A1 | C2CD4A |
| MSH6 | CD3D | RIPOR1 | CXCL10 | RND2 | IL9R |
| HSPA1A | CXCL10 | WWC2 | CCR7 | ELL | CD3D |
| HSPA1B | VAMP3 | SLC35G2 | ICOS | FZD4 | CCL22 |
| INHBB | CARD8 | ST6GAL2 | PTGFR | H2BC21 | CHAF1B |
| ANOS1 | CD274 | ST6GAL2 | BHLHE22 | BLZF1 | CD2 |
| CCN3 | ARHGAP25 | ZNF461 | CD247 | SCEL | CD1B |
| NTSR1 | NUGGC | ZNF816 | CD3D | SGCE | IL2RB |
| SERPINE1 | APOL6 | CPT1C | TNFRSF9 | BAIAP3 | MMP25 |
| PLAG1 | DLG3-AS1 | WTIP | P2RY10 | SLIT2 | GPR25 |
| PRELP | CCR5 | TSHZ2 | TNFRSF9 | CEP57 | CD3D |
| PTPRM | CCL22 | UBE2QL1 | SCUBE1 | USPL1 | ME2 |
| PURB | COQ2 | RAB6D | ZNF596 | N4BP2L2 | CARD16 |
| TGFA | INTS9 | ZNF385C | SLAMF1 | HSPH1 | RRM2 |
| ZNF134 | BMP2K | MTIF3 | GSR | MID2 | SIRPG |
| CMAHP | PDCD1 | PPP1R32 | LAG3 | DNAJB4 | IDO1 |
| PPFIA4 | IPO9-AS1 | BEX5 | PTGER2 | LZTS1 | MMP25 |
| SGCE | IGSF6 | BEX5 | CCL22 | AAK1 | MAP4K1 |
| ZSCAN12 | CD247 | IFITM10 | CXCR6 | CADM1 | MEI1 |
| MAGI2 | TMEM156 | HOXA-AS3 | UBASH3A | DNAJC2 | COQ2 |
| TSPAN2 | SLAMF7 | LINC01836 | CXCR6 | HCAR1 | GPR25 |
| MLLT11 | P2RY13 | VAX2 | IL17A | OSTM1 | CFB |
| SWAP70 | CD3D | TCEAL2 | CHRM3-AS2 | C6orf15 | WDR78 |
| RASGRP3 | CD3G | SPATA46 | CHRM3-AS2 | TNFRSF19 | SDK1 |
| C6orf15 | RAPH1 | NOG | LOC101927745 | ERBIN | C2CD4A |
| C6orf15 | TTBK2 | CYP19A1 | CHRM3-AS2 | SEMA3G | CIITA |
| BET1L | CXCL3 | HHIPL2 | IL22RA2 | GALNT16 | LGALS17A |
| MS4A4A | IDO1 | ECRG4 | XCR1 | ZNF512B | IL12RB1 |
| TERF2IP | GTF2E2 | NALCN | CNR2 | HECW2 | MEI1 |
| SGTB | CD247 | IGFL1 | GSDMC | SIGIRR | CXCL2 |
| PIP4P2 | SNX10 | CAMK2B | KLRC1 | NDRG4 | TIGIT |
| SCNM1 | COQ2 | MYOZ3 | CHRM3-AS2 | LINC01711 | GRIN3A |
| ZNF606 | SAA2 | ABCA3 | DNASE1L3 | DYNC2H1 | CD1B |
| MYCT1 | CTLA4 | CALB2 | ST7-AS1 | ZFHX4 | CLEC9A |
| SLC35G2 | TIGIT | CRIP2 | NOS2 | FAM214B | CD3E |
| TRIM11 | CD3D | DTNA | KYNU | MPIG6B | GPR18 |
| CDADC1 | IL2RB | LPAR1 | IDO1 | AARSD1 | CXCR6 |
| ARHGAP24 | ICOS | GFRA1 | GPR25 | COG3 | CASP1 |
| TOMM40L | CD3D | GRP | FLT4 | ZNF394 | COQ2 |
| CCDC183 | CXCR6 | GRP | FAM131B | FAM126A | SH2D1A |
| CEP19 | SIRPG | GRP | CCDC8 | SNORD14E | IL2RB |
| HEXIM2 | CD247 | GRP | C14orf28 | GUSBP11 | CHRM3-AS2 |
| GIPC3 | CD3G | GRP | LINC02035 | FANK1 | DNASE1L3 |
| C19orf18 | GPR18 | GYPC | CALHM6 | GABPB2 | IL2RB |
| SHISA4 | CALHM6 | HSPA1A | CABIN1 | SDR16C5 | CXCL13 |
| FNDC5 | CD1B | HSPA1A | TSR1 | FAM13C | KCNA3 |
| ELFN1 | CD274 | HSPA1A | CCDC71L | GXYLT1 | IL2RB |
| LOC441204 | LINC00996 | MYH11 | TMEM119 | RTL5 | RASGRP1 |
| FAM229B | SIRPG | MYLK | FGL2 | BEX5 | LTF |
| NOG | GBP6 | CCN3 | ADH6 | C5orf46 | KLRD1 |
| JAKMIP2 | IL22RA2 | CCN3 | CHRNA5 | NEMP2 | MAP4K1 |
| NOG | CCDC36 | CCN3 | NFAM1 | TDRKH-AS1 | CD3G |
| SUSD5 | JAKMIP1 | CCN3 | LRTOMT | SYN2 | CHRM3-AS2 |
| NALCN | NCR1 | NPR3 | CHRM3-AS2 | TRIM9 | IL17A |
| HOTAIR | TBX4 | SERPINE1 | VPS9D1-AS1 | IGFL1 | PLD4 |
| LINC00958 | NCR1 | PLN | CXCL13 | FBXO43 | CHRM3-AS2 |
| DDIT3 | CEP55 | PPP2R5B | COQ2 | IGFL1 | FASLG |
| S1PR3 | CD247 | PTH1R | KCNA3 | CAMK2B | GBP6 |
| ERG | CD3G | DPF2 | COQ2 | ATRNL1 | CLNK |
| FABP4 | NCR3 | SALL2 | LINC00996 | HAND2-AS1 | KLRC1 |
| FUT1 | TNFRSF18 | SLC2A3 | NKG7 | CCDC151 | CD1B |
| GOLGA2 | PARP3 | SP4 | CD247 | HOTAIR | ZNF831 |
| GRP | YPEL1 | NR2F1 | SNX18 | IGLON5 | ACKR4 |
| GRP | LOXL4 | TGFB2 | ICOS | HAND2-AS1 | DPEP3 |
| GRP | MROH8 | TRO | UBASH3A | GRP | SIGLEC7 |
| HNMT | PBK | PPFIA4 | FAM21EP | FAM43B | CHRM3-AS2 |
| HOXB3 | CD3E | CRLF1 | LINC00114 | SLC2A1-AS1 | IFNG |
| HSPA1A | COQ3 | ITGBL1 | CCR4 | OTOAP1 | CHRM3-AS2 |
| NDUFC2 | CASP1 | GGPS1 | PBK | LINC02367 | BFSP2 |
| CCN3 | TNF | HAND2 | GVINP1 | SUSD5 | IL9R |
| NPR3 | LILRA4 | MID2 | POU2AF1 | ANGPT2 | CD247 |
| PDE6D | CD3D | R3HDM2 | RNF213 | CALB2 | NCR3 |
| PLA2G5 | CD1B | ABLIM3 | GFI1 | COMP | HK3 |
| PLN | CTSW | ARC | CENPS-CORT | CREB1 | TRIM69 |
| TERF2 | NLRC5 | PLA2G15 | ME2 | CRYAB | CLEC4A |
| THBS4 | LOC339192 | EML2 | PCCB | GDI1 | UCP2 |
| CHST3 | CXCR6 | C20orf194 | ICOS | GLUD1 | GSR |
| UTP14C | NEIL2 | SUSD5 | GRIP2 | GRP | ZNF223 |
| ABCC9 | TBX21 | ANGPTL4 | SEMA7A | GRP | NDST2 |
| LRRC17 | TNFRSF9 | ISYNA1 | CD3D | GRP | RGMA |
| SEMA3A | GBP5 | TBC1D22B | ME2 | GRP | ARHGAP22 |
| DEAF1 | CD3D | TP73-AS1 | SLAMF1 | GRP | WDR78 |
| STMN2 | SLAMF1 | RIMKLB | GPR171 | GRP | DUOXA1 |
| ARC | KLRD1 | PTBP2 | CD3G | GUCY1B2 | IFNG |
| DDAH2 | LAP3 | TSPYL2 | FAM83F | HOXB4 | CIITA |
| ACOT9 | COQ2 | POPDC2 | P2RY13 | HOXB4 | MMP25 |
| SLC22A17 | CIITA | HS1BP3 | SOCS1 | HSPA1A | ELP3 |
| HIGD1B | UBASH3A | OBI1 | CD2 | INHBB | LAG3 |
| GDAP2 | CXCR6 | GALNT14 | TIFAB | EIF3E | CXCL1 |
| CC2D2A | CD3G | TMEM204 | NOS2 | KCNG1 | CHRM3-AS2 |
| TSPYL2 | CALHM6 | ARHGAP10 | IRF4 | MGP | HLA-DRB6 |
| EPB41L4A | IFNG | ZFHX4 | LIPC | NFATC4 | CXCR6 |
| ZNF484 | SIRPG | ZNF606 | CD274 | CCN3 | SARM1 |
| SETDB2 | IL2RB | APH1B | IDO1 | SERPINE1 | HLA-DQA1 |
| SLC7A6OS | IL12RB1 | AIF1L | GFI1 | PRKCH | LAG3 |
| SNORD14E | CLEC4A | COG3 | AGPAT5 | S100A1 | GPR25 |
| ZFAND2A | CD3D | C1orf198 | IRF1 | SOX11 | ESR2 |
| TRABD2A | CASP1 | SCIN | DNASE1L3 | UBE2E2 | LAG3 |
| ISM1 | CASS4 | PHLDB2 | CD3G | TNIP1 | LAP3 |
| CNST | CD3E | NXPE3 | SIRPG | DUSP14 | CD3D |
| KCTD21 | CCL22 | AHNAK2 | IL12RB1 | KLF12 | TNFRSF9 |
| BEX5 | CCR4 | FBXO32 | MPEG1 | LIMCH1 | IKZF1 |
| NRROS | DNASE1L3 | CPT1C | BHLHE22 | RAB21 | MOCOS |
| BAIAP2-DT | GBP4 | ISM2 | LOC339192 | ATG2A | CD3D |
| ANKRD65 | TNFRSF9 | LCA5 | UBASH3A | KIAA0556 | IL2RB |
| LOC101929709 | LINC00996 | PIANP | TPBGL | FNBP4 | CD3D |
| CAMK2B | NCR1 | SAMD14 | TNFRSF9 | CADM1 | UBASH3A |
| IGFL1 | TRAT1 | SAMD14 | TIGIT | RANBP6 | ODF3B |
| CRTAC1 | KIR2DL4 | FAM13C | GPR25 | MYEF2 | GPR25 |
| C5orf46 | SUCNR1 | ADGRF5 | IL2RB | SOX18 | CALHM6 |
| LINC01655 | C2orf66 | MAMDC2 | TRG-AS1 | RBM22 | TYMS |
| COL4A2-AS1 | ADGB | FAM171A2 | IL18RAP | MOSPD1 | ODF3B |
| NOG | LRFN5 | TMEM240 | LAX1 | NUFIP2 | C2CD4A |
| NOG | SLFN12L | TPTEP1 | CD226 | ROBO3 | GPR25 |
| BEX4 | TRAK1 | C20orf202 | MAL | ARHGAP10 | UBASH3A |
| ABCC10 | PARP3 | MXRA7 | CD2 | ARHGAP24 | TIGIT |
| INHBB | KIAA1755 | MRPS31P5 | LINC01555 | FBXL20 | CCL22 |
| CCN3 | NCF1 | ZNF529-AS1 | DNASE1L3 | SNORD14E | ITGAL |
| PLOD2 | KIF13A | ELMOD1 | BFSP2 | SNORD14E | L1TD1 |
| NOL3 | CD2 | LURAP1L-AS1 | IL26 | PPP1R14A | WRN |
| ADGRL2 | SIRPG | NOG | SLC24A4 | CFAP36 | CD3D |
| ZNF451 | CXCR6 | HAP1 | LHX2 | TM4SF18 | SIRPG |
| CCDC8 | GPR25 | PCBP3 | LINC00996 | TYW5 | SIRPG |
| PLPP7 | GPR25 | THORLNC | IFNG | DPY19L3 | CCL22 |
| TTC14 | CXCR6 | TCEAL2 | LHX2 | SAMD11 | SIRPG |
| TPTEP1 | ADGRE4P | LINC01602 | LINC01648 | ZNF467 | MMP25 |
| LINC01655 | GPR25 | GAP43 | ACKR4 | FUT11 | IL2RB |
| ANGPTL1 | CHRM3-AS2 | NXNL2 | LINC02273 | PHYHD1 | UBASH3A |
| ERICH6B | KIR2DL4 | NKX3-2 | CD1A | PAN3 | KIF13B |
| SLC25A34-AS1 | BFSP2 | BNIP3 | CCL22 | KCTD1 | LINC00996 |
| ACTC1 | TLR8 | CAV2 | TNFRSF18 | CHSY3 | FASLG |
| CALB2 | LYL1 | SERPINH1 | GSR | GRID2IP | LINC00996 |
| CAMK2B | B3GAT1 | CDKN2D | CD3D | ATXN7L3B | IRF1 |
| GAP43 | LINC00996 | CRIP2 | SOCS1 | SMIM10 | IKZF1 |
| GRP | FPR1 | DTNA | CAMK1G | SMIM10L2B | C16orf71 |
| GRP | FZD2 | EPB41L1 | CXCL10 | LOC100288748 | DNASE1L3 |
| GRP | SELL | GRP | BMP5 | SH3PXD2A-AS1 | CXCR6 |
| GRP | ARMCX1 | GRP | ICAM3 | BCHE | FAM86B2 |
| GRP | RHOJ | GRP | ITGB3 | ARC | CYP4F22 |
| GRP | SLC9B2 | GRP | RASGRP3 | ECRG4 | CD1E |
| GRP | ADGRG5 | GRP | DUOX1 | ANGPT4 | KLRC1 |
| GRP | PELI3 | GRP | LAX1 | CAMK2B | TOGARAM2 |
| NPR3 | TNIP3 | GRP | NHEJ1 | SYN2 | LGALS17A |
| PRKG1 | IDO1 | GRP | C16orf46 | FAM66C | ZNF831 |
| RASGRF2 | CXCR6 | GRP | ZNF555 | LINC02257 | LINC02273 |
| RYR3 | CHRM3-AS2 | GRP | CNIH3 | ISM2 | KCNK13 |
| TPM2 | RTCB | GRP | LOC152048 | TCEAL2 | ADORA2A |
| PPFIA4 | GVINP1 | GRP | CCSER1 | FAM218A | BFSP2 |
| GPRASP1 | GBP5 | GRP | SEMA3F-AS1 | CALB2 | IL2RB |
| SMUG1 | COQ2 | H2BC5 | NOS2 | CLK3 | FAM83F |
| TMEM59L | RPL10L | HSD17B1 | GPR25 | COMP | NFS1 |
| ATP10A | CXCR6 | HSPA1A | MICALL1 | S1PR3 | IKZF1 |
| CC2D2A | FOXP3 | HSPA1A | TOE1 | FGF1 | TNFRSF9 |
| ZNF665 | LINC00114 | INHBB | LTK | FGF1 | RASGRP1 |
| FAM117A | CD2 | INHBB | PAPLN | GPM6A | XCR1 |
| PCGF1 | CDC45 | KCNJ5 | IL9R | GRP | BOD1L1 |
| RAB42 | CXCR6 | NPR3 | LOC100129540 | HOXC4 | INSC |
| POC5 | C2CD4A | SERPINE1 | CCDC61 | NPR3 | SCN8A |
| LOC153684 | ICOS | PDE1A | BHLHE22 | TPM2 | ATP5PB |
| ZNF385C | SIRPG | PHKG1 | FASLG | HAND2 | TNF |
| PPP1R32 | CXCR6 | PLAG1 | CD80 | GJC1 | LINC00996 |
| AMIGO2 | ZBED4 | PTPRN | KIR2DL4 | CEP164 | CXCR6 |
| ZNF114 | CHRM3-AS2 | SLC1A3 | CD244 | LAMP5 | CLEC7A |
| LINC02257 | DBH | SPP1 | MMP9 | HSPB7 | CD86 |
| NOG | HTR4 | H2BC21 | FECH | ZNF606 | LINC00996 |
| GNGT1 | CRTAM | LDB2 | TBC1D10C | UTP23 | FECH |
| LINC01655 | RIMBP3 | LRRC17 | SLAMF1 | AMIGO2 | PISD |
| POU6F2 | IFNG | LILRB3 | GPR25 | C5orf46 | NLRC4 |
| ARHGDIG | DNASE1L3 | EXOSC8 | INTS10 | TDRKH-AS1 | IDO1 |
| ATP1B2 | TNFSF14 | IFFO1 | CXCR6 | GNGT1 | LINC01648 |
| CACNA2D1 | TNFRSF9 | C6orf15 | KCNIP4 | FOXD3 | CLNK |
| CACNA2D1 | BHLHE22 | CHST15 | CCL22 | HOTAIR | H4C2 |
| CALB2 | PTGDR2 | AIG1 | CD3D | LOC101928988 | CHRM3-AS2 |
| CTF1 | CTLA4 | RIPK4 | CD3D | IGFL1 | MTNR1A |
| GRP | SLC16A2 | TTC17 | CD3D | IGFL1 | PGAM4 |
| GRP | SYNJ1 | LRIF1 | CD3E | ADRA1B | NRG1 |
| GRP | STBD1 | ATP8B2 | FOXP3 | CALB2 | CXCL13 |
| GRP | ULK2 | JCAD | TBC1D10C | CALB2 | CCDC163 |
| GRP | GPSM1 | LMF1 | LAG3 | CALB2 | APOBEC3F |
| GRP | PIWIL2 | SCNM1 | ELP3 | COL10A1 | CXCL9 |
| GRP | CEP76 | ZNF671 | LINC00996 | CSRP2 | IDO1 |
| GRP | JAZF1 | CRISPLD1 | UBASH3A | DDIT3 | REEP4 |
| GRP | ZSCAN16-AS1 | EIF2A | LAP3 | GRIK5 | NRG1 |
| HOXB7 | WARS1 | SYDE1 | CALHM6 | GRP | ALPL |
| HSPA1A | MTHFD1L | CPT1C | IL18RAP | GRP | MMP19 |
| INHBB | SLC22A5 | ARMH4 | TNIP3 | GRP | DDR2 |
| INHBB | HDAC4 | ERFE | LINC00996 | GRP | PTGER2 |
| CCN3 | BST1 | DTX3 | CCL22 | GRP | LIPM |
| CCN3 | ZNF620 | ANO5 | ZNF831 | GRP | INCA1 |
| NPR1 | CD247 | JAZF1 | DNASE1L3 | GRP | RELL1 |
| NPR3 | ENTPD1-AS1 | BEX5 | PARVG | KISS1 | IDO1 |
| POLR2C | LAP3 | SHISA2 | DNASE1L3 | MYLK | MPEG1 |
| PRKD1 | CD274 | ZNF528-AS1 | LOC105377623 | NGF | UBASH3A |
| PTS | COQ2 | NOG | CCR3 | NPR3 | KYNU |
| RBMS1 | F2RL2 | OCM | BFSP2 | NPR3 | KCNH4 |
| SLC19A1 | C2CD4A | OCM | CHRM3-AS2 | NTSR1 | GRAP2 |
| TEF | CD3D | FLJ31356 | IFNG | PALM | TNFRSF18 |
| THBS4 | MEOX1 | DUXAP8 | IL17A | SGCD | CTLA4 |
| TPM2 | TTLL12 | CAMK2B | C22orf15 | SLC15A2 | SIRPG |
| VEGFB | TAP1 | TYRP1 | SH2D1A | SOX11 | SLC16A7 |
| BAIAP3 | CXCR6 | WNT7B | GPR25 | SPP1 | PRPS2 |
| HAND2 | ITK | HHIPL2 | ZNF831 | TH | DNASE1L3 |
| COX17 | IRF1 | ERCC1 | CD3D | TPBG | CD3E |
| MID2 | CXCR6 | GRP | DBH-AS1 | TYRP1 | RASGRP1 |
| TRIM58 | GPR25 | CCN3 | LAMC3 | VDAC2 | GSR |
| RAI14 | MPEG1 | NTSR1 | LHX2 | TRADD | CXCL2 |
| NGRN | C2CD4A | SURF2 | TYMS | HOMER3 | CD3D |
| RAB6B | SIRPG | GPRASP1 | NCR3 | HAND2 | SH2D1A |
| PTRH2 | COQ2 | ADAMTS5 | LINC00996 | HAND2 | MEI1 |
| BTN2A3P | CXCR6 | RBMS3 | CD1B | SOX13 | NF2 |
| TNFRSF19 | IKZF1 | SMCO4 | NOS2 | ARMCX2 | MOCOS |
| RNF20 | TYMS | ZNF248 | CD247 | PRG4 | IL12RB2 |
| BEX4 | IFIH1 | CDC73 | CD2 | ANGPTL4 | TRIM22 |
| SEMA3G | CXCR6 | DIPK2B | CD274 | KLHDC8A | TNFRSF9 |
| LY6G6C | TRPA1 | SLC35G2 | GPR18 | CENPJ | C2CD4A |
| EEPD1 | PARP3 | SNORD14E | NFAM1 | BEX4 | GTPBP3 |
| POLR2M | GBP4 | GLMP | FBXO6 | RELCH | C2CD4A |
| SNORD14E | ERI1 | TPM3P9 | DNASE1L3 | LINC01711 | NRG1 |
| METTL23 | IRF1 | CNST | TRIM69 | ZNF34 | CD3E |
| ISM1 | MN1 | KCNT2 | CLNK | FNDC1 | CXCL9 |
| DQX1 | CD2 | CDO1 | CHRM3-AS2 | FAM167B | GZMB |
| PLPP4 | CXCR6 | TM4SF19-AS1 | CHRM3-AS2 | INTS4 | C2CD4A |
| MAMSTR | IL12RB1 | ACTC1 | RASGRP1 | TTYH2 | CXCR6 |
| GPX8 | CFB | CALB2 | ATXN7L2 | ALG10B | IL12RB1 |
| ASPDH | GPR25 | CHRNA3 | SLAMF1 | VSIG10L | GFI1 |
| MOSMO | IDO1 | CRYAB | MOB3B | ZNF385C | CD3G |
| SOX11 | LRRC8C-DT | GRIK5 | LINC00996 | LOC339803 | UBASH3A |
| TMEM59L | TREML1 | GRP | CPM | KIAA1211L | CD3D |
| PNMA8B | JAKMIP1 | GRP | AKAP7 | IGFL1 | CDKL1 |
| CCDC81 | CHRM3-AS2 | GRP | SH2D3C | C5orf46 | LOC100506585 |
| ST3GAL6-AS1 | TIFAB | GRP | VPS53 | PCP4L1 | CASS4 |
| SLC35F1 | HDC | GRP | TARS3 | CD27-AS1 | MFSD2A |
| C8orf88 | SLAMF1 | HOXC4 | GPR18 | HOXA-AS3 | P2RY10 |
| PHYHIP | CD1B | LY6H | LINC00242 | JMJD7 | GPR25 |
| CYP4F8 | CHRM3-AS2 | MAP1A | CCL22 | LOC102723566 | CTLA4 |
| DKK1 | IL17A | MPZ | CXCR6 | GABRB2 | BFSP2 |
| SEPTIN7 | SLC39A8 | NPR3 | HDAC9 | IGFL1 | SLC31A2 |
| CST2 | RNU4-2 | RIT1 | CXCL10 | TRIM6 | IFNG |
| DES | HLA-DQB1 | SGCA | GFI1 | DUXAP8 | CHRM3-AS2 |
| DTNA | SLC6A12 | H3C6 | NAT1 | C1GALT1C1L | LINC01555 |
| DTNA | ARMH1 | PPFIA4 | IQCH | PRRT4 | ADGRE4P |
| GRP | SARM1 | NOG | BMPER | KRT81 | GPR25 |
| GRP | PCDH17 | SOX13 | HIRA | NOG | FAM87B |
| GRP | SMIM8 | GPRASP1 | CTLA4 | LINC02257 | LRP1-AS |
| GRP | TRPV4 | AKT3 | CCL22 | METTL24 | KCNA3 |
| GRP | ZNF527 | ARC | BTG3-AS1 | APLP1 | LINC01555 |
| GRP | NXPE3 | LAMP5 | CD72 | NKX3-2 | RASGRP1 |
| GRP | PPM1L | TMEM59L | CEACAM3 | CALB2 | PPM1J |
| GRP | FAM151A | HEYL | FOXRED2 | DDIT3 | LRPAP1 |
| GRP | FBXO48 | HSPB7 | NCKAP1L | DMPK | FOXA1 |
| GRP | SIGLEC14 | PDZD4 | UBASH3A | EEF1A2 | FFAR2 |
| GRP | LOC100996419 | POLR2J4 | FFAR2 | FOXC1 | CD8A |
| HSPA1A | GEMIN4 | FRMD6 | FOXP3 | GRP | ACAN |
| HSPA1A | PIGO | ZNF707 | CCDC134 | GRP | CSF3R |
| HSPA1A | MED11 | TPTEP1 | NRG1 | GRP | DGCR11 |
| INHBB | TIGD6 | LOC100507291 | CXCR6 | GRP | SLC2A9 |
| INHBB | GIMAP8 | GRP | PTHLH | GRP | SYTL3 |
| MAOB | CXCR6 | FCRLB | IL17A | GRP | RNF144B |
| GADD45B | PARP3 | TMEM221 | IL9R | GRP | UBA6-AS1 |
| CCN3 | CTSW | FLJ16779 | IL17A | GRP | LOC105371592 |
| CCN3 | SAMD4A | GRP | RHCG | HSPA1A | SART3 |
| CCN3 | CD163L1 | NPR3 | DBH | HSPA1A | NUP37 |
| CCN3 | IQCH-AS1 | SLC14A1 | CCL25 | IL1RAP | CTLA4 |
| NTSR1 | SOX6 | ARC | A1BG-AS1 | INHBB | HEMK1 |
| PDE1A | TIGIT | 114299 | LHX2 | ADAM11 | KIR2DL4 |
| SFRP2 | CXCL9 | CBY2 | INSC | MPP3 | CTLA4 |
| PIP4K2B | MFHAS1 | SAP25 | CHRM3-AS2 | NDN | CXCL9 |
| PPFIA4 | PKDREJ | VAX2 | IL22RA2 | NGF | FMO3 |
| CNTNAP1 | CXCR6 | FOXD4 | IL17A | CCN3 | SLC16A2 |
| MAFB | CD3E | PRKAA2 | CHRM3-AS2 | CCN3 | SLC24A1 |
| PRG4 | KLRC1 | ZFHX4 | ECE1-AS1 | CCN3 | TRMT44 |
| SPRY1 | MFHAS1 | TEX43 | DNASE1L3 | NPR3 | IFNG |
| LYVE1 | ICOS | ZNF385D | CLNK | NTSR1 | TMEM26 |
| TMSB15A | ICOS | BNIP3 | TIGD2 | PDE6B | JAKMIP1 |
| ZC3H13 | RNF213 | CRYAB | CD3E | MAP2K7 | MFHAS1 |
| DOCK9 | NUDT18 | DAXX | SLC39A14 | MAP2K7 | PARP3 |
| LAMP5 | CD28 | DTNA | SOCS2-AS1 | PTH1R | CP |
| RGCC | DMTN | ERG | CCR7 | RAD9A | C2CD4A |
| RGCC | SETD3 | FABP4 | KCNJ2-AS1 | ATXN1 | IL2RB |
| C6orf15 | AKAP5 | GRP | LILRB4 | SPP1 | MRFAP1L1 |
| EGFL7 | CD3D | GRP | AAK1 | TTF1 | NEIL2 |
| EGFL7 | SOCS1 | GRP | ADGRL2 | PPFIA4 | MDH1B |
| MTMR12 | COQ2 | GRP | WFDC1 | PER2 | C2CD4A |
| TNFRSF19 | STAT4 | GRP | OGFRL1 | EIF2B2 | COQ2 |
| PIP4P2 | CALHM6 | HOXC4 | GPR25 | MED6 | CD3D |
| ZBTB26 | IL2RB | HSPA1A | DNMT1 | RRAGB | CD3D |
| CCDC90B | CD3D | INHBB | MED31 | GPNMB | CXCL10 |
| RASL11B | LINC00996 | CCN3 | CHN1 | KLF12 | SIRPG |
| MMRN2 | CALHM6 | NPR3 | SUCLG2-AS1 | VPS45 | CD3D |
| FAM110A | NOS2 | NTSR1 | FASLG | FNDC3A | ATP6V1B2 |
| RASSF5 | IDO1 | NTSR1 | LRP2BP | ATP11B | PARP3 |
| TAF3 | IL2RB | PFKFB3 | IRF1 | ADAT1 | C2CD4A |
| TATDN1 | CD3D | CDK14 | CXCR6 | LAMP5 | BEST4 |
| ENKD1 | CD3D | PIM1 | REEP4 | ZBTB20 | IL9R |
| PPP1R15B | CXCL2 | PLAG1 | ICOS | HCAR1 | DLG3-AS1 |
| AHNAK2 | GBP5 | SEPTIN4 | IKZF1 | SAR1B | CD3D |
| PNCK | IL17A | PTPRN | DBH-AS1 | ANGPTL4 | ITGAE |
| TMEM86A | CCR7 | SLC11A1 | IL12RB1 | INSIG2 | COQ2 |
| TMTC2 | IL2RB | SNTB1 | IRF1 | ZNF639 | CD3D |
| SMIM29 | PARP3 | SOX11 | GIMAP5 | RAB6B | P2RY13 |
| SCUBE3 | TNFSF14 | SPP1 | PSMB2 | ARID4B | GBP4 |
| RBPMS2 | CD274 | SPP1 | RANGAP1 | TBC1D13 | PARP3 |
| CASTOR3 | CXCR6 | SPP1 | DNAJC8 | TNFRSF19 | PARVG |
| KLHL31 | LINC00996 | VIP | MYL5 | NRIP3 | ICOS |
| SAMD12-AS1 | TMIGD2 | ZNF33B | C2CD4A | PNMA8B | GPR82 |
| KTN1-AS1 | SIRPG | ZNF175 | CD247 | TSHZ3 | CXCR6 |
| NAALAD2 | CHRM3-AS2 | H2BC21 | MOB3B | CHID1 | CXCL3 |
| C5orf46 | CD244 | JAKMIP2 | ZNF831 | JHY | CASS4 |
| METTL24 | JAKMIP1 | PREB | GSR | WWC2 | CTLA4 |
| LINC02544 | LINC02027 | ZNF443 | ERI1 | MPIG6B | H2BC13 |
| ANGPT2 | IL12RB1 | TBC1D8 | FAM83F | LY6G6C | APOBEC3G |
| DTNA | KIR2DL4 | KCNE4 | CCR7 | LY6G6C | ZC3H12D |
| FLI1 | SIRPG | LAMP5 | CD96 | AHNAK2 | CTSW |
| FMO4 | CD247 | TMEM59L | ADGRE3 | SHE | RASGRP1 |
| HSPA1A | GBP1 | LMOD1 | SASH3 | FAM131A | CALHM6 |
| INHBB | SPN | HSPB7 | CIITA | EGFLAM | MMP25 |
| ITIH3 | ZNF831 | RGCC | FUT2 | PNCK | RBM15-AS1 |
| KCNQ3 | LHX2 | RRNAD1 | IRF1 | SPINDOC | CENPM |
| MCC | TIGIT | ANGPTL4 | GBP4 | ISM2 | ISL2 |
| PCDH7 | TIGIT | PCDH12 | IDO1 | ZNF570 | SAA2 |
| SERPINE2 | SOCS1 | SYT17 | CTLA4 | CNST | CD3D |
| PLN | CTLA4 | CMTR2 | C2CD4A | CREG2 | ZNF831 |
| PTH1R | SLFN12L | BEX4 | CDC25A | CNPY4 | CD3E |
| RDX | IL10RA | BEX4 | BRPF1 | PHYHD1 | SIRPG |
| SCN1B | UBASH3A | BEX4 | FCHSD2 | TMEM105 | DNASE1L3 |
| TERT | GPR25 | JPH2 | CD1C | IGFL1 | GRIP2 |
| MAFB | CALHM6 | RIMKLB | CD38 | TMEM200B | CIITA |
| HDAC5 | POLR3H | BAHCC1 | CCL22 | SMIM10L2B | RBM15-AS1 |
| NMUR1 | GPR25 | CWC22 | DCTN6 | PCP4L1 | ADGRE3 |
| BTN2A2 | CXCR6 | FAM204A | ERI1 | LOC100133091 | GFI1 |
| FBXL7 | IKZF1 | POPDC2 | CTLA4 | SRRM5 | LINC00996 |
| IL20RB | FASLG | DHRS12 | C2CD4A | TDRKH-AS1 | CTLA4 |
| TNFRSF19 | GPR18 | ZNF696 | FAM83F | LINC01655 | ZNF831 |
| TNFRSF19 | LINC00996 | NUF2 | ME2 | UPK2 | KCNG2 |
| MOSPD1 | GZMA | GALNT15 | RASGRP1 | LINC02257 | KCNG2 |
| ZNF512B | CIITA | ARMH4 | INSC | HAND2-AS1 | CNR2 |
| FAM234B | LINC00996 | SATB2-AS1 | FGL2 | FADS6 | KIR2DL4 |
| ZBTB26 | EPHA10 | INTS6L | CCL22 | CDH23 | IL17A |
| SRCIN1 | MMP25 | SUGT1P3 | CIITA | GALNT14 | BTLA |
| SNORD14E | GEN1 | ZNF530 | MMP25 | CORO6 | GPR25 |
| GNPDA2 | CXCR6 | IGFL1 | LOC339192 | FCRLB | BHLHE22 |
| SPIN3 | NEURL1 | ZBED6 | SIRPG | IGFL1 | ANKRD34A |
| ADAMTS17 | CD1B | ARC | KLF1 | HOXC4 | EOMES |
| AMZ2P1 | CXCR6 | PNMA8B | IFNG | GRP | ATP6V0D2 |
| BEX5 | SLA2 | IGFL1 | BLK | TRIM58 | LGALS17A |
| ANKRD65 | TIGIT | IGFL1 | GPR174 | APBB1 | S1PR4 |
| TRAF3IP2-AS1 | LINC00996 | NAP1L2 | BTLA | AR | CD226 |
| NOG | LINC01750 | HAND2-AS1 | CLNK | DPYD | GBP5 |
| GPIHBP1 | IL17A | TCEAL2 | AMZ1 | DTNA | IQCN |
| MYOSLID | CD226 | IGFL1 | KLRG1 | ERV3-1 | CCL22 |
| CAMK2A | CLNK | KLHDC8A | GPR25 | GRP | CLTCL1 |
| NGF | PDE6G | ST6GAL2 | CAMK1G | GRP | IQCG |
| PDZRN4 | ZNF831 | C1orf105 | IFNG | GRP | FAM161B |
| LOC101929295 | CHRM3-AS2 | MIR1915HG | CLECL1 | GRP | ATXN2-AS |
| MYOSLID | ZNF831 | LINC01977 | FASLG | HSPA1A | BLMH |
| CALB2 | TNFRSF18 | ALOX15 | UBASH3A | HSPA1A | RPUSD2 |
| CALB2 | SLAMF7 | ATP1B2 | TESPA1 | HSPA1A | GCC1 |
| CCN3 | ERI1 | BNIP3 | ASPHD2 | LIMS1 | APOL6 |
| NPR3 | FMO3 | BNIP3 | ACTR3B | BCAM | SOCS1 |
| SGCA | TBC1D10C | CALB2 | UBASH3A | LYL1 | IL12RB1 |
| AOC3 | CXCL10 | COMP | IL2RB | NAB2 | CD3D |
| HAND2 | PDCD1LG2 | CST2 | CLIC2 | CCN3 | KIAA0895 |
| LMOD1 | CXCL10 | DTNA | MTNR1A | CCN3 | CLEC7A |
| HSPB7 | CD48 | DTNA | FCRL5 | NPR3 | CDH23 |
| BAIAP2-DT | COQ2 | F13A1 | CD7 | NPR3 | SHC4 |
| C8orf88 | SLA2 | GRP | CD72 | NTSR1 | NLGN4X |
| COL9A3 | CD247 | GRP | IKZF3 | P2RX7 | FASLG |
| DBP | SIRPG | GRP | C19orf38 | RAG1 | LINC01555 |
| SNAPC1 | CXCR6 | GRP | LYPD5 | SLC4A3 | GPR25 |
| ARHGAP29 | TNFRSF9 | GRP | PRRT3 | SLC11A1 | SIRPG |
| PCP4L1 | LILRP2 | GTF2F1 | GSR | TGFB3 | CXCR6 |
| FOXC2 | TRG-AS1 | MR1 | FOXP3 | THBS3 | ODF3B |
| IGFL1 | CHRM3-AS2 | HSPA1A | RNF34 | THBS4 | LILRB5 |
| NOG | H2AZ1-DT | INHBB | MAPKBP1 | VIP | TMEM273 |
| SEMA3D | CHRM3-AS2 | CCN3 | DAAM2 | LAT2 | FOXP3 |
| CALB2 | CCR7 | CCN3 | APOBEC3G | SCG2 | CD101 |
| CALB2 | SPN | CCN3 | IPPK | PPFIBP2 | CD3D |
| CALB2 | SLC9A7 | CCN3 | ZNF615 | NR1D1 | MAEA |
| CALB2 | FAM78A | NPR1 | MMP25 | GPRASP1 | TNFRSF9 |
| CSRP2 | FOXP3 | PALM | S1PR4 | LPGAT1 | CHMP7 |
| EPB41L1 | MMP12 | RARA | PARP3 | N4BP2L2 | FGL2 |
| GRP | DNMT3B | RNASEL | EPHA10 | SEMA6C | SIRPG |
| GRP | FOXS1 | STXBP1 | TRAF3 | ADAMTS5 | P2RY10 |
| GRP | RGS12 | TEC | CXCR6 | ADAMTS5 | ICOS |
| GRP | CCNJL | NR2F1 | CD3E | KIFAP3 | UBIAD1 |
| GRP | RELT | TGFB1I1 | CD3D | NACAD | GPR25 |
| GRP | ARHGAP19 | TH | OGDHL | ARC | ATP8B3 |
| KIF5A | FASLG | LAT2 | IDO1 | UPF2 | ELP3 |
| MDM4 | C2CD4A | ST8SIA4 | SIRPG | FBXO3 | ERI1 |
| NPR3 | ATP8B4 | COLQ | GPR25 | C6orf15 | ZBTB37 |
| SERPINE1 | SMAP2 | CDK13 | RNF213 | C6orf15 | ZNF233 |
| PRELP | MOXD1 | EIF3C | IDO1 | TMOD2 | SLAMF1 |
| SLIT3 | CCR7 | NOG | TDGF1P3 | FAM178B | GRIP2 |
| TH | FFAR2 | NR1D1 | GAK | DCDC2 | CEACAM3 |
| AOC3 | CD3D | SOX13 | HTT | PNMA8A | CASS4 |
| C2CD2L | FAM83F | LRRC17 | CD3G | BEX4 | MCUB |
| HSPB7 | ARHGAP25 | FST | CD3G | KCNK15 | FASLG |
| RPS6KA6 | IDO1 | POLR3C | IRF1 | BLOC1S5 | CD2 |
| RAB9B | RASGRP1 | MID2 | CIITA | FKBPL | CD3D |
| BEX4 | SMARCAL1 | ARC | KMO | MICAL1 | NOS2 |
| KCMF1 | IRF1 | CAMSAP2 | TRIM69 | MRPL9 | LAP3 |
| MTHFSD | FAM83F | HCAR1 | GVINP1 | ZBTB10 | RABL2B |
| TSPAN10 | TESPA1 | C6orf15 | LPP-AS2 | WWC2 | PDCD1 |
| ISM2 | STX18-AS1 | ARHGEF4 | IL18RAP | PRR3 | IL2RB |
| SDR16C5 | NKG7 | CYB5R2 | CTLA4 | BHLHB9 | DNASE1L3 |
| PLPP4 | CCL22 | ADAMTSL4 | CTLA4 | COLEC12 | P2RY10 |
| ZNF385C | SLA2 | TNFRSF19 | IL15 | OR51E2 | GPR25 |
| GDPD1 | IDO1 | TNFRSF19 | ZNF132 | AIF1L | SIRPG |
| LOC100128398 | GPR25 | TNFRSF19 | LOC101927989 | USHBP1 | GPR25 |
| LINC00649 | CD3G | GALNT17 | NRG1 | ZCCHC7 | ELP3 |
| NOG | MOV10L1 | GALNT14 | IL9R | ZNF512 | IL2RB |
| GRIK2 | FCRL4 | SUGCT | GZMM | HOPX | CTLA4 |
| ARC | PGAM4 | COG3 | XPO7 | G6PC3 | CXCL2 |
| CACNB3 | CD3D | JAM3 | CALHM6 | EFHC1 | CXCR6 |
| CALB2 | MTCL1 | CCDC115 | TYMS | CPT1C | FASLG |
| COL11A2 | IL17A | HVCN1 | IKZF1 | ISM1 | KLHL6 |
| DAPK1 | CCL22 | LRCH3 | IL2RB | RDH10 | NOS2 |
| F13A1 | IGSF6 | FAM207A | CENPM | CBY2 | LINC00996 |
| FLT4 | TBC1D10C | SGF29 | NOS2 | CCZ1B | CXCR6 |
| GABRD | CXCR6 | RBP7 | CTSW | BCL6B | PTPN7 |
| B4GALNT1 | GPR25 | NIBAN1 | CXCR6 | PAN3 | EDEM1 |
| GPR4 | IL2RB | NKAIN4 | ACKR4 | SUGT1P3 | ICOS |
| GRP | CTSW | ISM1 | KIF1C-AS1 | NHLRC4 | GPR25 |
| GRP | LYL1 | CILP2 | NCR3 | SIRPB2 | GPR25 |
| GRP | PTN | ALDH1L2 | CTLA4 | BEX5 | ZAP70 |
| GRP | VEGFC | LCA5 | TNFRSF9 | AMIGO2 | CD2 |
| GRP | ZNF771 | GDPD1 | CXCR6 | TRAM2-AS1 | IL2RB |
| GRP | LMLN | SPRED3 | GPR25 | RBIS | CXCL10 |
| GRP | APOBEC3D | FAM111A-DT | CCL22 | SMIM10L2B | MEFV |
| GRP | RNF207 | HES7 | CLNK | ZHX1-C8orf76 | CD3G |
| GRP | MIR3142HG | PCOLCE2 | IFNG | ZNF529-AS1 | MMP25 |
| HSPA1A | HMBS | GJA3 | IFNG | LINC02257 | IL22RA2 |
| HSPA1A | IRF8 | CDC5L | LEPROTL1 | NOG | LOC101928994 |
| HSPA1A | SLC1A4 | DTNA | BTLA | ZFHX4 | IFNG |
| HSPA1A | HMGXB3 | PHC2 | WARS1 | LOC101927124 | IFNG |
| HSPA1A | TRIM65 | GCLC | CD3D | TH | CD1B |
| INHBB | KIF9 | GRP | TRDMT1 | NOG | NAALAD2 |
| INHBB | PIF1 | GRP | REM1 | ARC | LOC100129540 |
| ITGA7 | CALHM6 | GRP | TMIGD3 | HCAR1 | THEMIS |
| KISS1 | CXCR6 | GRP | TRAF3IP3 | GALNT17 | LINC01555 |
| MAP1A | CXCR6 | GRP | DCLK2 | ISM2 | CCR8 |
| MAP1B | LAG3 | GRP | CATSPER2P1 | RAB6D | DNAJC28 |
| GADD45B | TNFAIP3 | GRP | C16orf95 | ZDHHC15 | LIPC |
| NOTCH4 | IDO1 | HSPA1A | PML | IGFL1 | IP6K3 |
| CCN3 | RASAL3 | HSPA1A | TNFRSF10A | LOC105370854 | IL22RA2 |
| NTSR1 | PDE10A | HSPA1A | UBAP2 | GRP | GZMK |
| ODF2 | CHMP7 | HSPA1B | KPNB1 | RGS17 | KIR2DL4 |
| PHKG1 | INSC | ING2 | CD2 | ARL17B | IFNG |
| PRELP | OLFML1 | MGP | CD53 | DYDC2 | LINC02332 |
| TNS1 | MPEG1 | GADD45B | NOS2 | NOG | NLRP7 |
| VLDLR | GFI1 | NAB1 | PPP2R2A | BNIP3 | APOBEC3D |
| ZSCAN9 | CD3E | CCN3 | C3orf18 | CACNB3 | SMAD4 |
| RNF8 | CD3D | CCN3 | CYB5RL | FGFR1 | CCL22 |
| TMCC2 | LINC00996 | PLAG1 | H3C7 | GRP | SP140 |
| EDAR | CALHM6 | PLAG1 | RNASEK-C17orf49 | GRP | SLC25A34 |
| PALLD | LCP1 | UBL3 | CASP1 | HSPA1B | PSMB10 |
| LAMP5 | GNGT2 | MAPK11 | CXCR6 | INHBB | CD247 |
| PDLIM3 | SLAMF7 | PTPRN | IL9R | KDR | IL2RB |
| EFEMP2 | SOCS1 | PURB | NLRC5 | CCN3 | CYP2D6 |
| SLC45A1 | ICOS | STRN | CD2 | PLN | DOCK8 |
| TNFRSF19 | GFI1 | MAP3K7 | CD3D | PURA | CCL22 |
| BEX4 | ELP2 | TH | GPR25 | RABIF | CD3D |
| RIMKLB | LAX1 | CNTNAP1 | CTLA4 | RBMS1 | TBC1D10C |
| HAMP | IL9R | CDK5R1 | GFI1 | RIT1 | INTS9 |
| SRCIN1 | PTPN7 | ARHGEF1 | TYMS | SKP1 | CASP1 |
| CARD11 | GSTO2 | ABCG2 | LINC00996 | THBS4 | TNFSF14 |
| TJAP1 | NLRC5 | SH3BP5 | SIRPG | TYRP1 | JAKMIP1 |
| ISM1 | IKZF1 | ARMCX2 | FOXP3 | MTERF1 | DNASE1L3 |
| LOC148709 | UBASH3A | STMN2 | LAX1 | CAVIN2 | CXCR6 |
| NUDT17 | CTLA4 | ZHX1 | TNFRSF11A | FOXH1 | CD1B |
| SLC51A | DNASE1L3 | ENPP4 | CD2 | NOL3 | ME2 |
| PRECSIT | GFI1 | KIFAP3 | MTFR1L | LY86 | CXCR6 |
| ZNF582-AS1 | TRG-AS1 | SCFD1 | CD3D | OGA | TYMS |
| SMIM10L2B | GPR18 | SSBP2 | LTA | HSPH1 | GNPDA1 |
| ZNF667-AS1 | TIGIT | TMEM59L | ZNF831 | STMN2 | GPR171 |
| OSER1-DT | CD3D | C5AR2 | CD1B | ARC | CD226 |
| SP2-AS1 | SIRPG | RGCC | LARS2 | LDOC1 | FOXP3 |
| LINC02257 | ANK1 | RGCC | RDH14 | MLXIPL | SOCS1 |
| TMEM59L | NCR1 | INSIG2 | CD3D | PAIP2 | CASP1 |
| SNHG31 | CHRM3-AS2 | SUCO | CD2 | HIKESHI | NEIL2 |
| HAND1 | CD244 | EMCN | SIRPG | GPRC5B | DNASE1L3 |
| BCHE | NLGN4X | SIDT1 | DNASE1L3 | GPRC5B | LAG3 |
| NOG | TTC34 | ZNF415 | FASLG | ASPN | CXCL13 |
| SUSD4 | CHRM3-AS2 | GNB4 | TBC1D10C | DEF8 | SOCS1 |
| NECAB1 | CD226 | ADGRL4 | IL2RB | ODR4 | CD2 |
| IGFL1 | DLG3-AS1 | ATG9A | NOS2 | HCFC1R1 | IRF1 |
| NPR3 | C12orf74 | OGFRL1 | CXCR6 | TXLNG | C2CD4A |
| ARC | IL17A | GGNBP2 | C2CD4A | LRP2BP | CD1B |
| ARC | SIGLEC11 | MYO15B | NOS2 | WWC3 | SASH3 |
| NAV3 | CCR3 | TMEM134 | CD3D | ATP10D | CXCR6 |
| GRP | EDN3 | SOX7 | TIGIT | RHBDF1 | PARP3 |
| GRP | RSPO3 | ELOF1 | NOS2 | ADAMTS10 | UBASH3A |
| NTSR1 | CCR3 | HOOK3 | CCL22 | HOOK3 | KLRB1 |
| AVPR2 | TBX21 | ARRDC4 | CD2 | FAM126A | FASLG |
| BNIP2 | CD2 | LOC93622 | CD3D | ZNF439 | LINC00996 |
| CACNA1C | CD3G | LMTK3 | CXCR6 | ARHGEF25 | LAG3 |
| CACNB1 | GFI1 | MACROD2 | IL17A | FAM131A | CD8A |
| CACNB3 | RELCH | SAMD11 | CD274 | ISM1 | CCDC200 |
| SEPTIN7 | LCP1 | DSTNP2 | IL2RB | LAYN | CTLA4 |
| COMP | GPR68 | INTS6L | IKZF1 | GRASP | UGT2B7 |
| COMP | HAVCR2 | SDK1 | GPR25 | ZNF610 | CD1B |
| CST2 | C3orf85 | ENDOV | CTLA4 | PIANP | KLRD1 |
| DGKA | IL2RB | YIPF6 | COQ2 | PAN3 | APOL6 |
| ENO2 | HLA-DMB | ZNF568 | CHRM3-AS2 | PAN3 | C2CD4A |
| FLT1 | IDO1 | MIR4435-2HG | CCL22 | CHSY3 | GPR25 |
| FLT3LG | FASLG | ECSCR | CXCR6 | TUBB2B | LAIR2 |
| GRP | ZNF273 | C8orf88 | NRG1 | SMIM10L2B | CHRM3-AS2 |
| GRP | ABCB9 | LOC101929240 | SOCS1 | CARD8-AS1 | CXCR6 |
| GRP | CARD9 | CRYBA4 | KIR2DL4 | ZNF528-AS1 | GPR82 |
| GRP | SNX22 | NOG | TMEM18-DT | KLHL4 | CCR6 |
| GRP | SYDE2 | HOXC6 | RPL10L | SUGT1P4-STRA6LP | CHRM3-AS2 |
| GRP | TTYH2 | HAND1 | IL17A | CARTPT | LGALS17A |
| GRP | TTC39A-AS1 | TCEAL2 | ACKR4 | LINC00562 | KIR2DL4 |
| GRP | KBTBD11-OT1 | HOTAIR | ARSH | C5orf46 | CCR8 |
| HSPA1A | PDCD7 | TCHH | CCR6 | HTR2B | CHRM3-AS2 |
| HSPA1A | RHBDF2 | NGF | KIR2DL4 | ARC | XCL2 |
| INHBB | ARHGEF6 | SOX11 | TSPAN32 | BHMT2 | IL17A |
| NOTCH4 | ITGAL | HAP1 | BFSP2 | SUSD5 | IL26 |
| CCN3 | ZNF839 | NOG | KLRD1 | DELEC1 | LINC01648 |
| NPR3 | IL9R | IGFL1 | APOBEC3H | NALCN | FLT3 |
| NPR3 | LGALS17A | LINC01655 | KLRD1 | INHBB | FDXACB1 |
| PLS3 | LCP1 | NXNL2 | CRLF2 | CCN3 | SCIMP |
| PTH1R | NLGN4X | NOG | PTPN5 | TPBG | CXCR6 |
| PTPN14 | GFI1 | GPM6A | KIR2DL4 | CRIP2 | CENPM |
| RTN2 | CYTIP | ARL4D | SIRPG | CRYAB | MANBA |
| SIAH2 | NCOA7 | GRP | SWSAP1 | GGTA1P | SIRPG |
| SMARCE1 | CD3D | CCN3 | CEACAM21 | GRP | HLA-DQB2 |
| TH | FASLG | CCN3 | FGD2 | GRP | TESK2 |
| VIP | CIITA | PRKCH | CTLA4 | GRP | TBC1D9 |
| PPFIA4 | KRBA2 | RARA | CD3D | GRP | TBC1D12 |
| GCNT3 | NOS2 | THBS4 | KLRG1 | GRP | MS4A6A |
| BCL7C | MCAT | DDAH2 | EPHB2 | GRP | CALHM6 |
| FEZ1 | SIRPG | LAMP5 | KLRB1 | NPR3 | UNC5C |
| ADAMTSL2 | IDO1 | LAMP5 | MYO7A | NPR3 | ZNF366 |
| PPP4R1 | CD3D | NCKAP5L | CXCR6 | SALL2 | SIRPG |
| CFDP1 | GTF2E2 | PHF6 | TNFRSF11A | SGCA | CXCR6 |
| CAP2 | DNASE1L3 | SNORD14E | MIR3142HG | THBS4 | CCL22 |
| ARID5A | APOL6 | ARMH4 | TRPA1 | TPM2 | SARS1 |
| STMN2 | CD3G | GPAT2 | DNASE1L3 | ZNF230 | CXCR6 |
| SYT11 | CCL22 | NPHP4 | SIRPG | HAND2 | SLA2 |
| ARC | DARS-AS1 | TMEM81 | SIRPG | LPGAT1 | MMP12 |
| BACE1 | CD3E | LOC101927851 | GPR25 | SPRY1 | MMP12 |
| RAB38 | GBP5 | PICART1 | CHRM3-AS2 | LAMP5 | SPN |
| RAI14 | CD3E | HOTAIR | LINC02159 | ADAMTSL4 | CCL22 |
| HEYL | IL7R | HOTAIR | CCL25 | CDKN2AIP | MMP12 |
| STAU2 | CD3E | BCHE | MIR34AHG | BEX4 | PLA2G12A |
| C6orf15 | CCT6B | HAND1 | GAPT | TLCD5 | CXCR6 |
| C6orf15 | CFAP69 | ART5 | IFNG | NOG | DTHD1 |
| EFEMP2 | CXCL9 | HOTAIR | IL26 | FCGR1CP | CCL25 |
| TNFRSF19 | CYP26B1 | NKX3-2 | DNASE1L3 | TCEAL2 | FAM87A |
| ZNF692 | SOCS1 | BCHE | NRG1 | PAEP | CCL25 |
| BEX4 | FGL2 | CALB2 | POMK | NOG | TREML1 |
| RIMKLB | LINC01555 | CALB2 | SPATA18 | HAND1 | CLEC12A |
| CC2D2A | DNASE1L3 | CALB2 | ZC3H12D | PCDHGA4 | IL22RA2 |
| SCUBE2 | TNFRSF9 | COMP | HOXA13 | CLEC4F | KLRC1 |
| ARHGAP10 | INSC | GJB2 | SLC39A14 | IGFL1 | SYNC |
| JHY | CD244 | GRP | GRK3 | MIR1915HG | CHRM3-AS2 |
| SRCIN1 | CD247 | GRP | HAS2 | HOXC8 | IFNG |
| RBP7 | DNASE1L3 | HOXC4 | GHRL | HAND2-AS1 | TTC24 |
| ZNF641 | CCL22 | HOXC6 | LINC01555 | CALB2 | BFSP1 |
| WTIP | LINC00996 | HSPA1A | MMP12 | CALB2 | ZBP1 |
| MPLKIP | CD3D | KRT10 | IRF1 | CCNH | COQ2 |
| SPTY2D1 | CD3D | NID1 | MMP12 | COMP | CD3D |
| SLC25A30 | CD8A | NPR3 | GDNF | DGKA | FAM83F |
| ENDOV | CXCR6 | NPR3 | COL23A1 | FAP | FOXP3 |
| KCTD1 | TNFRSF9 | NPR3 | A2M-AS1 | GNGT1 | GPR18 |
| FAM221A | CXCR6 | NTSR1 | CCR4 | GRP | TMEM131L |
| ZNF713 | FASLG | PRELP | IL2RB | GRP | DMXL2 |
| PEAK3 | GPR25 | SNCG | CD48 | GRP | CSGALNACT1 |
| ELFN1 | GFI1 | VIP | SNX10 | GRP | TICRR |
| ELFN1 | FFAR2 | STK24 | MMP12 | GRP | ANKRD42 |
| PCAT19 | TIGIT | C1QL1 | CXCR6 | HOXC4 | MASP2 |
| HAND1 | ADGRE4P | ARC | LOC100507564 | INHBB | E2F2 |
| SFTA1P | CHRM3-AS2 | LAMP5 | C16orf54 | KCNMB1 | GBP5 |
| BNIP3 | GNLY | LAMP5 | SLC9A9 | MYO5A | CTLA4 |
| CALB2 | DBH-AS1 | DEXI | FAM83F | CCN3 | RELL1 |
| GRP | PIK3R6 | NGRN | ELP3 | NTSR1 | LOC101930370 |
| GRP | RNF152 | BEX4 | MAPK9 | RGS16 | LEPROTL1 |
| HSPA1A | SH2B3 | SPSB3 | CXCR6 | SALL2 | P2RY10 |
| HSPA1A | KNSTRN | LEAP2 | CXCR6 | SOX11 | ADAMTS3 |
| INHBB | DNASE1L3 | SAMD11 | CCL22 | TPM2 | COX14 |
| INHBB | ACAP1 | TMEM105 | TRPA1 | TRPC1 | P2RY10 |
| INHBB | JAML | AMIGO2 | HHLA2 | UBE2E2 | F2RL2 |
| INHBB | MAST4 | IGFL1 | NLRC4 | VLDLR | CXCR6 |
| MEF2A | TRIM69 | LINC00548 | CHRM3-AS2 | PPFIA4 | SMG1P5 |
| NTSR1 | KLRC1 | SNHG20 | FAM83F | SYNGR1 | SLAMF1 |
| OXTR | GPR25 | NAPA-AS1 | CXCR6 | NOG | ANO2 |
| SNCG | CTLA4 | ADAMTSL3 | TBX21 | HAND2 | AOX1 |
| UQCRB | IRF1 | C19orf18 | LAIR2 | HAND2 | CLIP4 |
| STX6 | ME2 | ANKRD34A | GPR25 | CIR1 | NCOA7 |
| CNPY3 | GSR | SUSD5 | LINC02273 | MAGI2 | RASGRP1 |
| STMN2 | TNFRSF9 | VAX2 | FLT3 | EDAR | CD2 |
| BAZ1A | CD2 | TMEM221 | CLNK | STON1 | IKZF1 |
| KIFAP3 | LUZP1 | NOG | ASTL | BTN2A1 | IRF1 |
| NACAD | TBX21 | LOC102724153 | IL17A | KIFAP3 | THUMPD3 |
| EML2 | ME2 | GNGT1 | LINC01101 | HSPB8 | PTPRC |
| TMOD2 | RASGRP1 | NOG | PLA2G1B | HSPB7 | CCR7 |
| PSMC3IP | CXCR6 | DPY19L2 | IL22RA2 | ANGPTL4 | BDKRB2 |
| PAIP2 | IRF1 | AVPR2 | CD1A | SCARA3 | IL2RB |
| CHMP3 | CASP1 | SUSD5 | CEND1 | PIP4P2 | RBL1 |
| NKAPD1 | CD2 | EMILIN3 | IL17A | BEX4 | FNBP1 |
| TNFRSF19 | CASS4 | GRP | TM6SF1 | BEX4 | OTUD1 |
| UGGT2 | CDCA2 | PTH1R | LTA | BCO2 | GPR25 |
| FAM234B | GPR25 | GNGT1 | IL9R | CARD19 | CXCL2 |
| AOPEP | EPHA10 | GRP | TCEAL7 | MPLKIP | COQ2 |
| ZNF480 | FECH | TMEM59L | INSC | ISM2 | SPTY2D1OS |
| HOXC11 | KIR2DL4 | SLC35F1 | CCR3 | TLCD5 | MMP25 |
| NGF | GPR174 | SLC35F1 | IFNG | CBY2 | LYRM9 |
| ANKRD53 | CHRM3-AS2 | STUM | JAKMIP1 | FAM162B | DNASE1L3 |
| TBX1 | CHRM3-AS2 | IGFL1 | MIR34AHG | IGFL1 | SPIB |
| C1GALT1C1L | SLAMF1 | ACTC1 | TESPA1 | ANXA2R | CD3G |
| ARL4C | GBP4 | CA11 | IDO1 | GPX8 | MPEG1 |
| GPRC5B | SIRPG | CALB2 | NEURL3 | SMIM10 | FOXP3 |
| JAM3 | DNASE1L3 | CRYAB | FBLN5 | ZNF667-AS1 | SLAMF1 |
| ARHGEF25 | SIRPG | CST2 | CD48 | PCAT19 | CD3G |
| GRP | BTC | DLX3 | DNASE1L3 | PCAT19 | SIRPG |
| GRP | LST1 | ACSL3 | NCOA7 | NOG | GATA1 |
| NOG | ANK1 | GFRA3 | UBASH3A | HAND1 | DEFA4 |
| REEP2 | CTLA4 | GRIK5 | THEMIS | GNGT1 | IL17F |
| RIMKLB | CCR7 | GRP | CD79B | IGFL1 | KIR2DL4 |
| IGLON5 | AKAP3 | GRP | SLA | GJA3 | SLC25A47 |
| GNGT1 | LOC100506885 | GRP | IRF9 | TCEAL2 | CD226 |
| PCP4L1 | IFNG | GRP | CHST12 | IGFL1 | LTA |
| SLC6A17 | LIPC | GRP | GPATCH2L | LINC01655 | CLECL1 |
| LINC01655 | BTLA | GRP | ATP10D | SLC26A10 | CRLF2 |
| TMEM59L | PTGDR | GRP | CRACD | ADCY1 | GPR25 |
| ARHGAP20 | ZNF831 | GRP | TMEM86A | NKX3-2 | RTN1 |
| LOC101927124 | CHRM3-AS2 | GRP | ZC3H12D | BTK | SIRPG |
| PCDHGA4 | CLNK | HSPA1A | FBXO7 | CACNB3 | DPH1 |
| SUGT1P4-STRA6LP | KIR2DL4 | KCNMB1 | SIRPG | CALB2 | IZUMO4 |
| CALB2 | IL2RA | MYH11 | SPI1 | CALB2 | NCF1 |
| CALB2 | MYO1G | NFKBIE | CENPM | COL5A2 | MMP12 |
| CHRNA3 | BHLHE22 | NGFR | P2RY10 | COL11A2 | IFNG |
| CYP1B1 | SLAMF1 | CCN3 | CXCL13 | CRMP1 | CXCR6 |
| GRP | ABCA1 | CCN3 | PRELID3A | DTNA | TNFRSF8 |
| GRP | C1orf162 | CCN3 | PSD3 | EEF1A2 | PDCD1 |
| NPR3 | MAPK10 | CCN3 | BCAS3 | EFNA4 | LAP3 |
| NPR3 | PIEZO2 | CCN3 | THSD4 | FGF13 | GIMAP5 |
| NPR3 | NCR3 | NPR3 | ANK1 | FOXC1 | GNLY |
| NTSR1 | GPR171 | NPR3 | LILRA2 | GRP | ZNF236 |
| SGCA | IRF4 | NTSR1 | TNIP3 | GRP | TOX2 |
| SGCA | SIRPG | NTSR1 | PARP15 | GRP | TMEM116 |
| TMOD1 | TNFRSF9 | NTSR1 | KCNRG | GRP | BHLHA15 |
| TPM2 | OGDH | OPCML | LIPC | HOXB3 | CXCL9 |
| TPM2 | SRSF7 | SALL2 | LAX1 | HSPA1A | PCYT2 |
| ZNF202 | C2CD4A | SPP1 | IARS1 | HSPA1A | PRC1 |
| PPFIA4 | TUBA3D | SPP1 | PPP1CC | HSPA1A | TXNL4A |
| MYOM1 | UBASH3A | SPP1 | JAGN1 | HSPA1A | POC1A |
| HAND2 | LAX1 | TPM2 | DNAJC5 | INHBB | EDNRB |
| TMCC2 | RASGRP1 | TRPC1 | TNFRSF9 | LMO7 | INTS14 |
| AP4S1 | CXCR6 | PCGF2 | TYMS | NGF | NRG1 |
| ABLIM3 | CCR7 | RNF112 | GPR25 | CCN3 | IRF9 |
| HSPB7 | CD300LF | H2BC21 | C2CD4A | CCN3 | MLYCD |
| NOX4 | BHLHE22 | PPFIA4 | ART3 | NPR3 | VIPR2 |
| HDGFL3 | CXCR6 | SYNGR1 | TIGIT | NPR3 | KRBA2 |
| BEX4 | USP38 | ZEB2 | CTLA4 | P2RX7 | TIGIT |
| CXorf21 | SIRPG | EDIL3 | CXCL9 | PHF1 | CHMP7 |
| LY6G6C | FCMR | MPHOSPH10 | INTS10 | PKD2 | TRIM69 |
| PLPP4 | KCNJ2 | MPHOSPH10 | PBK | PODXL | IRF1 |
| GGN | CD244 | CD2BP2 | ASAH1 | PRKCH | ITGAL |
| CBY2 | SIRPG | RABL2A | CXCR6 | PTH1R | ADGRE1 |
| TMEM105 | CTLA4 | VSIG4 | HLA-DMB | S100A13 | PBK |
| ANKRD65 | GBP5 | CADM1 | SIRPG | SCN4B | TIGIT |
| ZNF528-AS1 | TIGIT | LAMP5 | ICOS | SLC4A3 | RASGRP1 |
| LINC02257 | CD1B | TMEM59L | SLC18A2 | SPP1 | ASAH1 |
| RGS9BP | IL26 | TMEM59L | BEGAIN | SPP1 | DHX15 |
| TCEAL2 | KLRC1 | RAI14 | NFXL1 | TFAP2C | ADGRG7 |
| CACNA1F | CHRM3-AS2 | ANGPTL4 | B9D1 | TNFAIP6 | IDO1 |
| CNP | IRF1 | TNFRSF19 | CTLA4 | GPANK1 | ME2 |
| CTF1 | GBP5 | TNFRSF19 | ZAP70 | MTERF1 | IDO1 |
| FGF13 | IFNG | BEX4 | MSL3 | H4C9 | C2CD4A |
| GRP | CRMP1 | CLK4 | IDO1 | H2BC21 | DLL1 |
| GRP | TEC | PDZD4 | LTA | CDC16 | SLC39A14 |
| GRP | MFSD4B | MEAK7 | CD3E | AKAP12 | CD7 |
| HOXC6 | CD226 | NLRC4 | TNFRSF9 | HS3ST2 | ZBTB32 |
| INHBB | PIGL | DLK2 | MMP25 | GNB5 | GBP5 |
| LTBP3 | GBP1 | WWC2 | CXCR6 | PLAC1 | GHRL |
| CCN3 | MFSD6L | CSRNP3 | IFNG | VSIG4 | GBP4 |
| NPR3 | GRIN3A | TRIM46 | CASS4 | RAB38 | CXCR6 |
| PRELP | ARHGEF6 | POLR3GL | INTS10 | EML2 | CHMP7 |
| RNASEL | IL2RB | HVCN1 | CXCR6 | FBXL3 | APOL6 |
| SQLE | LAP3 | SNORD14E | CHAC2 | HSPB7 | LSAMP |
| VIP | CCL22 | PPP1R14A | CD2 | CSDC2 | BHLHE22 |
| MPDZ | SLAMF1 | PPP1R14A | NAGPA | PDZD11 | GSR |
| NR1D1 | LETM1 | ISM1 | FANCB | CRLF3 | CD3D |
| PLPPR4 | LINC01555 | ISM1 | MEIS1 | SYT17 | IRF4 |
| GFPT2 | CXCR6 | ISM2 | WNT9A | NDE1 | C2CD4A |
| SNAPC5 | ELP3 | SYNPO2 | IDO1 | PALMD | MEI1 |
| MID2 | CTLA4 | MUC20 | RBM47 | SSH3 | CXCL3 |
| N4BP3 | CIITA | SAMD14 | SLAMF1 | TNFRSF19 | ITGA8 |
| ARC | ARPC4-TTLL3 | FAM13C | NRG1 | TNFRSF19 | ADGRE2 |
| NPTXR | MMP25 | NALCN | KLRC1 | VPS11 | PARP3 |
| LAMP5 | STAT4 | IGFL1 | LILRA6 | GJC2 | CXCR6 |
| HCAR1 | CYSLTR1 | CCDC9B | IL2RB | HECW2 | SLAMF1 |
| RGCC | DMAC2 | ANKRD65 | GFI1 | PDZD4 | PTGDR |
| LGALSL | CCNI2 | ANKRD65 | P2RY13 | ISLR2 | GPR25 |
| FKBP14 | FECH | SMIM10L2B | AKAP3 | ELOVL5 | CXCL3 |
| TNFRSF19 | PDCD1 | SMIM10L2B | NDUFA6-DT | BCO2 | IFNG |
| TNFRSF19 | SIRPG | NBPF20 | IL17A | HHIPL1 | TIGIT |
| THSD1 | CTLA4 | LOC101929340 | ICOS | HDGFL2 | IRF1 |
| BEX4 | GALNT11 | LINC02257 | LINC01270 | TSPAN18 | SLAMF7 |
| BEX4 | CDCA3 | CDK5R2 | IFNG | MIDEAS | CD3E |
| MAP3K7CL | CXCR6 | GABRB2 | LINC02273 | GNRHR2 | UBASH3A |
| ARHGAP10 | PDCD1 | NOG | C22orf15 | ISM2 | KDM4D |
| VASH2 | LINC01555 | CSRNP3 | CLNK | ISM2 | RASGRP4 |
| C1orf105 | NRG1 | NGF | SLC47A1 | ZNF570 | SLA2 |
| ISM1 | FAM20A | TMEM217 | IFNG | PLPP4 | XRRA1 |
| ISM1 | DOCK10 | SKIDA1 | CHRM3-AS2 | PIANP | MS4A2 |
| CILP2 | CD244 | HOTAIR | LHX2 | SCUBE3 | RASGRP1 |
| ZNF570 | GPR65 | HOTAIR | IFNG | KCTD1 | SLAMF1 |
| SDR16C5 | CALHM6 | NECAB1 | GIMAP5 | RXFP4 | CALHM6 |
| NHLRC4 | FASLG | EBF2 | CHRM3-AS2 | BEX5 | CCR7 |
| GALNT18 | GBP4 | EDARADD | CD1B | BEX5 | PIWIL2 |
| TOB1-AS1 | CTLA4 | NALCN | ADGRE4P | BEX5 | CD300LF |
| LOC101927851 | FFAR2 | VPS33B-DT | CHRM3-AS2 | TUBB2B | PDCD1 |
| KIAA1549L | GPR25 | HES7 | CCR6 | ANKRD37 | IDO1 |
| CLEC1A | SLAMF1 | CALB2 | IFI30 | KIF7 | CXCR6 |
| IGLON5 | FLJ38576 | CDH2 | GPR25 | STUM | LRP1-AS |
| TCEAL2 | NEDD8-MDP1 | GMFB | NOS2 | PNPLA7 | FFAR2 |
| ERICH6B | CHRM3-AS2 | GRP | AP4S1 | SPRED3 | GHRL |
| KCNK17 | GPR25 | GRP | ZNF407 | C16orf74 | GFI1 |
| NOG | ECE1-AS1 | GRP | LINC00852 | PCP4L1 | MS4A2 |
| BCHE | CD1B | GRP | HYKK | PCAT19 | ICOS |
| CDK5R2 | BTLA | GRP | ANKDD1B | LOC100506258 | CHRM3-AS2 |
| NALCN | LINC02158 | HSPA1A | EXOSC2 | ZNF687-AS1 | GBP5 |
| TRIM23 | CXCR6 | INHBB | KYAT1 | LINC02560 | NRG1 |
| CCNT1 | NUDT18 | INHBB | WDR91 | HCAR1 | CD244 |
| F13A1 | CXCL13 | INHBB | DIABLO | HCAR1 | ATP6V0D2 |
| GPR21 | NEIL2 | CCN3 | CMKLR1 | CRTAC1 | CHRM3-AS2 |
| GRP | CETP | CCN3 | PMEL | IGFL1 | SLC47A1 |
| GRP | SEPTIN4 | CCN3 | EFCAB11 | IGFL1 | MFSD2B |
| GRP | CARF | CCN3 | ZC3H12D | AQP5 | KIR2DL4 |
| GRP | TMEM121 | NPR3 | FLT3 | OPRL1 | GPR25 |
| GRP | PSTK | NPR3 | GOLGA6L10 | HOXC6 | CRTC3-AS1 |
| GRP | C8orf37 | NTSR1 | ZIK1 | IGFL1 | RPS2P32 |
| GRP | ZNF852 | NTSR1 | ZNF286B | HOXC-AS1 | IL26 |
| GRP | HLA-DQB1-AS1 | SERPINE1 | BCR | ARC | TEX101 |
| HSPA1A | NOS2 | PRELP | PRR16 | OSGIN2 | CD3D |
| INHBB | RASAL3 | NR2F1 | SHROOM4 | CALB2 | SYNC |
| GADD45B | APOL2 | TMOD1 | NCR3 | CALB2 | LINC01594 |
| NAB1 | CLPX | VIP | CD300LF | CAV2 | IDO1 |
| NPR3 | SH2D1B | PCGF2 | TXNL4A | COL5A2 | CXCL2 |
| NTSR1 | ANKRD33B | SSPN | CXCR6 | CRYAB | IL2RB |
| OLR1 | IKZF1 | H4C8 | CCL22 | CST2 | IFFO1 |
| CLDN11 | IFNG | AOC3 | IL7R | DTNA | SIRPB1 |
| PCDH7 | ICOS | AOC3 | FGL2 | ENO2 | NKG7 |
| PLAG1 | IL18RAP | NR1D1 | DPY19L1 | FABP4 | FASLG |
| PLOD2 | CWF19L1 | C2CD2L | CDCA2 | GLI3 | GPR25 |
| PSD | ICOS | TNK2 | FOXA1 | GRP | FER |
| RDX | IL2RB | TDRKH | MOCOS | GRP | PRKD1 |
| RIT1 | NUDT18 | KIFAP3 | AREL1 | GRP | PTGIR |
| RIT1 | MPEG1 | NACAD | MAL | GRP | SNED1 |
| RTN2 | TNFAIP8L2 | CEP68 | CD3E | GRP | CBLN3 |
| SALL2 | CASS4 | LAMP5 | LINC00114 | HSPA1B | SRSF7 |
| TWIST1 | CXCR6 | DNM3 | LINC00996 | INHBB | ELAC1 |
| SCG2 | NCF1 | RABGEF1 | GFI1 | INHBB | ADAMTS9 |
| MPDZ | TNFRSF9 | C6orf15 | TBC1D32 | INHBB | HMBOX1 |
| ELMO1 | DNASE1L3 | POLR1D | ASAH1 | MPP2 | THEMIS |
| UST | GBP1P1 | RAB9B | NRG1 | CCN3 | KBTBD8 |
| CNPY3 | TAP1 | RIPPLY3 | LINC00114 | CCN3 | ZC3H12C |
| MLLT11 | CD274 | FBXL19 | CDC45 | CCN3 | INKA1 |
| LYPD3 | CXCR6 | ENOX1 | BHLHE22 | NPR3 | RUFY4 |
| ANGPTL4 | CCL4 | HEATR1 | C2CD4A | P2RX7 | LINC00996 |
| COPZ2 | IL2RB | TNFRSF19 | GNGT2 | PIM1 | IRF1 |
| HIGD1B | SLAMF1 | TNFRSF19 | INSC | PURB | C2CD4A |
| SYT17 | TIGIT | TNFRSF19 | SCIMP | RDX | FMNL1 |
| FBXL12 | PAFAH2 | TNFRSF19 | THAP7-AS1 | SFRP4 | HCK |
| TNFRSF19 | HIVEP3 | ADGRL4 | IL7R | SGCD | TNFRSF9 |
| TNFRSF19 | MEI1 | PYCR3 | CENPM | SLIT1 | LHX2 |
| BEX4 | CD3E | GDPD3 | CD2 | SP4 | CIITA |
| FAM234B | RASGRP1 | ARHGAP10 | CD3G | TIAM1 | GPR25 |
| LMF1 | TBC1D10C | SUGCT | DNASE1L3 | TPBG | EPHA10 |
| TMUB2 | IRF1 | JHY | IL18RAP | UBE2E2 | CD8A |
| GAL3ST4 | CALHM6 | TCEAL4 | NCOA7 | VIP | GBP5 |
| ZNF606 | CD3G | ZCCHC7 | COQ2 | FZD7 | CD3D |
| EFHD1 | P2RY13 | BRSK1 | MMP25 | DNAH17 | TRG-AS1 |
| TMEM163 | TRG-AS1 | MCEE | CD3D | ZSCAN12 | CIITA |
| AOPEP | IL2RB | PCGF1 | ELP3 | ABCC9 | HDC |
| PCED1B | IL2RB | ORAI3 | CD3D | LHFPL6 | CXCL9 |
| CHURC1 | CD3D | NLRP3 | ICOS | CFDP1 | IRF1 |
| CTHRC1 | CXCL10 | ISM1 | HIVEP3 | TDRKH | IL2RB |
| RBP7 | UGT2B7 | ARMH4 | LINC00996 | KLF12 | SLAMF1 |
| ISCA2 | CD3D | LINC00662 | GFI1 | SLC4A1AP | CD2 |
| KCTD11 | CD3D | FGD5 | IDO1 | RAI14 | TANGO2 |
| PUS10 | CD247 | ZNF567 | CD247 | HERC4 | IL2RB |
| PPM1K | SIRPG | SCAMP5 | CALHM6 | RGCC | MAN1A1 |
| ZNF467 | DNASE1L3 | SDR16C5 | CD48 | PHF11 | SOCS1 |
| TLCD5 | MEI1 | MUC20 | NCOA7 | HDAC7 | NLRC5 |
| LOC339803 | GPR18 | WFDC10B | UBASH3A | SEMA5B | IL18RAP |
| BEX5 | MMP25 | WFDC10B | TMCC1-AS1 | ASPN | CD300A |
| ZNF404 | LINC00996 | NRBP2 | SOCS1 | ODR4 | C2CD4A |
| RELL1 | CCL22 | STUM | TREML1 | TTC17 | C2CD4A |
| NKAPL | CHRM3-AS2 | SPRED3 | HDC | PCDHB15 | FASLG |
| CDH19 | BFSP2 | MIR17HG | DNASE1L3 | RNF20 | CASP1 |
| GNGT1 | ADGRE1 | ECSCR | PTPN7 | BEX4 | TRIM22 |
| GRP | THEMIS | SMIM10L2B | KCNA3 | BEX4 | ABI3 |
| NPR3 | MPL | HHIP-AS1 | RASGRP1 | JPH2 | IL21R |
| FXYD1 | TIFAB | NOG | B3GAT1 | DIP2B | C2CD4A |
| TYRP1 | HDC | TCHH | IL22RA2 | NAPB | IL2RB |
| ARC | LHX2 | LINC02257 | LOC100128059 | CCDC136 | IL18RAP |
| HAND2-AS1 | GIMAP5 | PYGM | CD1B | KLC2 | CD3D |
| H2BC18 | NUGGC | ALDOC | POC1A | LYNX1 | GPR25 |
| LINC01119 | CHRM3-AS2 | BMP6 | SLAMF1 | COLEC11 | GPR25 |
| MKNK1-AS1 | CHRM3-AS2 | CACNB3 | CHAF1B | TLE6 | GPR25 |
| GRIK2 | LIPC | RCBTB2 | IL2RB | TMEM163 | TBX21 |
| GRP | ZNF132 | GRP | FBN2 | ST6GAL2 | ANKRD33B |
| NALCN | CRLF2 | GRP | PRDM8 | ZFAND2A | COQ2 |
| HAP1 | LGALS17A | GRP | LINC01144 | TM4SF18 | CIITA |
| NOG | PPP2R2B | GRP | GTF2H2C | TMEM170A | C4orf19 |
| C8orf88 | CD244 | GRP | ZGLP1 | CPT1C | CASS4 |
| ADM | CD3D | GRP | GAPLINC | ARL8A | TNFRSF1B |
| ARL4D | GBP5 | GRP | LNCTAM34A | ISM1 | LILRB5 |
| CAMLG | IRF1 | HSPA1A | BUB1B | ZNF548 | CXCR6 |
| GPX3 | CD2 | INHBB | C8orf58 | ZNF569 | NRG1 |
| GRP | GBP1P1 | KCNG1 | CLECL1 | CNIH3 | ICOS |
| GRP | LOC100506585 | LAIR1 | CXCR6 | EXOC8 | ERI1 |
| HSPA1A | RAD51 | MPZ | UBASH3A | SHLD1 | C2CD4A |
| HSPA1B | HNRNPD | PPP1R12B | CCL22 | RUNDC3B | NRG1 |
| NPR3 | LINC02273 | NTSR1 | CD226 | TSNARE1 | CD3D |
| NTSR1 | DNAH7 | P2RX7 | LTA | INTS6L | IL12RB1 |
| PGM5 | UBASH3A | PIK3C3 | CXCR6 | DAGLB | CD3D |
| TGFB1I1 | FGL2 | SEPTIN5 | GFI1 | THSD7A | GIMAP5 |
| EIF3C | CXCR6 | ELOVL4 | CARD17 | BCL6B | CD8A |
| AP1S2 | CCL22 | TPD52L2 | GSR | HERC2P3 | LGALS17A |
| MMP24 | MEI1 | VEGFC | CIITA | SIGLEC15 | GPR25 |
| AAK1 | SIRPG | ADAM12 | GBP5 | ATG9B | MMP25 |
| WDR47 | CCL22 | AOC3 | SLAMF8 | LOC374443 | CD3G |
| SCFD1 | CD2 | CCPG1 | CXCR6 | FIBIN | GBP4 |
| TMEM59L | LHX2 | NREP | F2RL2 | CDC37L1-DT | LINC00114 |
| ARMCX3 | PARP3 | CHST10 | RASGRP1 | NOP53-AS1 | FASLG |
| CTDSP1 | LAP3 | ZNF234 | CXCR6 | SNORD53B | KIR2DL4 |
| JHY | MIR34AHG | HSPH1 | HACD3 | IGFL1 | LILRA1 |
| NCALD | CXCR6 | ARC | MPL | LINC02257 | CCR3 |
| SNORD14E | ACP3 | CNRIP1 | TBC1D10C | SCRG1 | BFSP2 |
| PPP1R14A | CALHM6 | MOXD1 | CXCR6 | TMEM151B | LINC02273 |
| LAYN | CIITA | FBXO10 | GPR25 | GABRB2 | SCN3A |
| DACT3 | RASGRP1 | CHST11 | ITGAL | NALCN | ABCD2 |
| DACT3 | TIGIT | PNPLA8 | CD2 | OVOL3 | IL17A |
| LCA5 | THEMIS | MTERF3 | PBK | CDK6-AS1 | CCL25 |
| GAST | CXCR5 | CALHM2 | CALHM6 | CAMK2B | TEX101 |
| LINC00840 | IFNG | TNFRSF19 | LILRB5 | IGFL1 | NTF3 |
| KIRREL3 | CLNK | MEIS3 | CIITA | NOG | CBLN2 |
| SPRY4-AS1 | IFNG | JPH2 | ZNF596 | H2BC7 | GPR25 |
| GDPD3 | DOCK5 | KIAA1586 | DNASE1L3 | ERFL | NCR3 |
| TM4SF18 | ICOS | DPEP2 | GPR25 | NGF | HDC |
| ZNF841 | CXCR6 | SOX17 | ICOS | PCDHB5 | LGALS17A |
| CALB2 | ITGAL | CCDC136 | THEMIS | CORO6 | CLECL1 |
| GRIK5 | TNFSF14 | TMEM204 | CD3D | NOTCH2NLR | IL17A |
| GRP | SLCO3A1 | ZCCHC7 | CD3D | APBB1 | TBC1D10C |
| GRP | CCDC113 | NEXN | GBP5 | CHML | KLRB1 |
| GRP | SHANK3 | SLC2A13 | DNASE1L3 | CREM | CCL22 |
| GRP | STEAP2 | CYYR1 | KLRB1 | S1PR3 | GBP5 |
| GRP | GPRIN3 | HSPA12B | CXCR6 | GOLGA2 | CHMP7 |
| PRELP | GBP4 | DNAJC24 | SIRPG | GPC1 | GBP4 |
| SNCG | CALHM6 | ZBTB46 | MMP25 | GRP | ADGRG7 |
| THBS2 | CNOT1 | ISM1 | CD80 | GRP | RASGRP4 |
| KIFAP3 | ABCD3 | ISM1 | DRC3 | GRP | ETFBKMT |
| KIFAP3 | LARS2 | OLFML2A | IL2RB | GRP | RPS2P32 |
| TMEM59L | COL23A1 | TMEM81 | CXCR6 | GRP | TCAF2 |
| CHIC2 | MMP12 | PAIP2B | DNASE1L3 | HSPA1B | HLA-DPB1 |
| SCUBE2 | CCR7 | SMIM10L2B | CD1B | INHBA | IL2RB |
| ANKRD24 | DNASE1L3 | ZDHHC15 | ZNF831 | INHBB | GBP5 |
| IGFL1 | CYP26B1 | FAM124A | TNFSF14 | MEIS1 | TNFRSF9 |
| SMIM10L2B | RASGRP1 | IGFL1 | ZNF366 | MGP | CCL5 |
| PCP4L1 | RASGRP1 | NOG | ZNF667 | CCN3 | MEOX1 |
| NECAB1 | IL22RA2 | PCP4L1 | TNFSF18 | CCN3 | POU6F1 |
| LINC01655 | CRLF2 | MYOSLID | CHRM3-AS2 | CCN3 | PIK3R5 |
| PCP4L1 | TLR7 | LINC01655 | TCL1A | CCN3 | HHAT |

Note: G*_i_*>G*_j_* represents the post-surgery high relapse risk.
