# Supplementary material for: Two novel qualitative transcriptional signatures robustly applicable to non‐research‐oriented colorectal cancer samples with low‐quality RNA
Source: J Cell Mol Med. 2021 Mar 14;25(7):3622–33. doi: 10.1111/jcmm.16467 (PMC8034468; doi:10.1111/jcmm.16467)
Supplement: Supplementary file 10 — Table S5 [file JCMM-25-3622-s003.doc]

| Table S5. Multivariable Cox proportional hazards regression analyses  for the relapse risk signature | | | |
| --- | --- | --- | --- |
| Variable | HR | 95%CI | *p* |
| Age | 1.0101 | 0.9658-1.056 | 0.660 |
| Gender(famale vs male) | 0.4587 | 0.1535-1.371 | 0.163 |
| Stage(II vs III) | 0.8116 | 0.2858-2.305 | 0.695 |
| MSI | 0.8510 | 0.3999-1.811 | 0.675 |
| Relapse predictive signature (high vs low) | 2.6457 | 0.9100-7.692 | 0.074 |
