# Supplementary material for: Two novel qualitative transcriptional signatures robustly applicable to non‐research‐oriented colorectal cancer samples with low‐quality RNA
Source: J Cell Mol Med. 2021 Mar 14;25(7):3622–33. doi: 10.1111/jcmm.16467 (PMC8034468; doi:10.1111/jcmm.16467)
Supplement: Supplementary file 11 — Table S6 [file JCMM-25-3622-s001.docx]

| Table S6. KEGG pathways enriched with the 319 genes | |
| --- | --- |
| KEGG pathway | *p*_value |
| Cytokine-cytokine receptor interaction | 2.20E-16 |
| Chemokine signaling pathway | 2.20E-16 |
| Osteoclast differentiation | 2.20E-16 |
| Toll-like receptor signaling pathway | 2.20E-16 |
| Jak-STAT signaling pathway | 2.20E-16 |
| Natural killer cell mediated cytotoxicity | 2.20E-16 |
| T cell receptor signaling pathway | 2.20E-16 |
| Prolactin signaling pathway | 2.20E-16 |
| ErbB signaling pathway | 2.22E-16 |
| TNF signaling pathway | 2.22E-16 |
| PI3K-Akt signaling pathway | 6.55E-15 |
| NOD-like receptor signaling pathway | 1.02E-14 |
| MAPK signaling pathway | 1.19E-14 |
| Neurotrophin signaling pathway | 2.34E-14 |
| Insulin signaling pathway | 4.65E-14 |
| Apoptosis | 1.10E-13 |
| Rap1 signaling pathway | 3.56E-13 |
| Intestinal immune network for IgA production | 2.40E-12 |
| Fc epsilon RI signaling pathway | 1.06E-11 |
| Hematopoietic cell lineage | 1.09E-11 |
| Ras signaling pathway | 1.10E-11 |
| Estrogen signaling pathway | 3.89E-11 |
| Focal adhesion | 8.47E-11 |
| NF-kappa B signaling pathway | 2.14E-10 |
| FoxO signaling pathway | 3.94E-10 |
| HIF-1 signaling pathway | 8.79E-10 |
| B cell receptor signaling pathway | 1.32E-09 |
| RIG-I-like receptor signaling pathway | 6.23E-09 |
| VEGF signaling pathway | 4.17E-08 |
| Gap junction | 4.86E-08 |
| GnRH signaling pathway | 8.33E-08 |
| Leukocyte transendothelial migration | 1.92E-07 |
| Axon guidance | 6.58E-07 |
| Progesterone-mediated oocyte maturation | 9.14E-07 |
| Platelet activation | 9.67E-07 |
| Cholinergic synapse | 1.79E-06 |
| Adipocytokine signaling pathway | 1.81E-06 |
| Thyroid hormone signaling pathway | 4.40E-06 |
| Oxytocin signaling pathway | 5.84E-06 |
| Regulation of actin cytoskeleton | 7.91E-06 |
| Long-term depression | 1.00E-05 |
| Inflammatory mediator regulation of TRP channels | 2.75E-05 |
| Serotonergic synapse | 3.53E-05 |
| Retrograde endocannabinoid signaling | 3.95E-05 |
| Fc gamma R-mediated phagocytosis | 4.29E-05 |
| mTOR signaling pathway | 5.55E-05 |
| Cytosolic DNA-sensing pathway | 7.60E-05 |
| Circadian entrainment | 3.02E-04 |
| Melanogenesis | 5.00E-04 |
| Dorso-ventral axis formation | 5.09E-04 |
| Antigen processing and presentation | 7.77E-04 |
| Dopaminergic synapse | 1.84E-03 |
| p53 signaling pathway | 2.99E-03 |
| Cell adhesion molecules (CAMs) | 3.68E-03 |
| Endocytosis | 3.96E-03 |
| Adrenergic signaling in cardiomyocytes | 6.42E-03 |
| Long-term potentiation | 9.40E-03 |
| Glutamatergic synapse | 1.24E-02 |
