# Supplementary material for: Two novel qualitative transcriptional signatures robustly applicable to non‐research‐oriented colorectal cancer samples with low‐quality RNA
Source: J Cell Mol Med. 2021 Mar 14;25(7):3622–33. doi: 10.1111/jcmm.16467 (PMC8034468; doi:10.1111/jcmm.16467)
Supplement: Supplementary file 13 — Supplementary Material [file JCMM-25-3622-s008.docx]

**Supplementary Figure S1.** **The retention rates of stable and significant opposite gene pairs in the remaining 154 stage II FF CRCs.**

**Supplementary Figure S2.** **The retention rates of the early diagnosis signature for all analysed samples. The cyan points represented normal and IBD samples, and red points represented CRC samples.**

**Supplementary Figure S3. The retention rates of the predicting post-surgery relapse risk signature for all samples in validation datasets. Cyan represented** **samples that were predicted to be low-risk relapse, and red represented samples that were predicted to be high-risk relapse. Triangles represented actually post-surgery relapse, and points represented actually post-surgery non-relapse.**

**Supplementary Figure S4. A-C: Kaplan-Meier curves of DFI for stage III CRC patients, excluding an 85-year-old patient relapsed in about four mouths.**

**Supplementary Figure S5. Protein-protein interaction (PPI) links including the 18 DEGs (purple).**

**Supplementary Scripts. The R scripts of the study.**
